# Supplementary material for: Air-stable redox-active nanomagnets with lanthanide spins radical-bridged by a metal–metal bond
Source: Nat Commun. 2019 Feb 4;10:571. doi: 10.1038/s41467-019-08513-6 (PMC6362165; doi:10.1038/s41467-019-08513-6)
Supplement: Supplementary file 1 — Supplementary Information [file 41467_2019_8513_MOESM1_ESM.pdf]

Air-stable redox-active nanomagnets with lanthanide spins radical-bridged by a metal-metal bond

Liu et al.

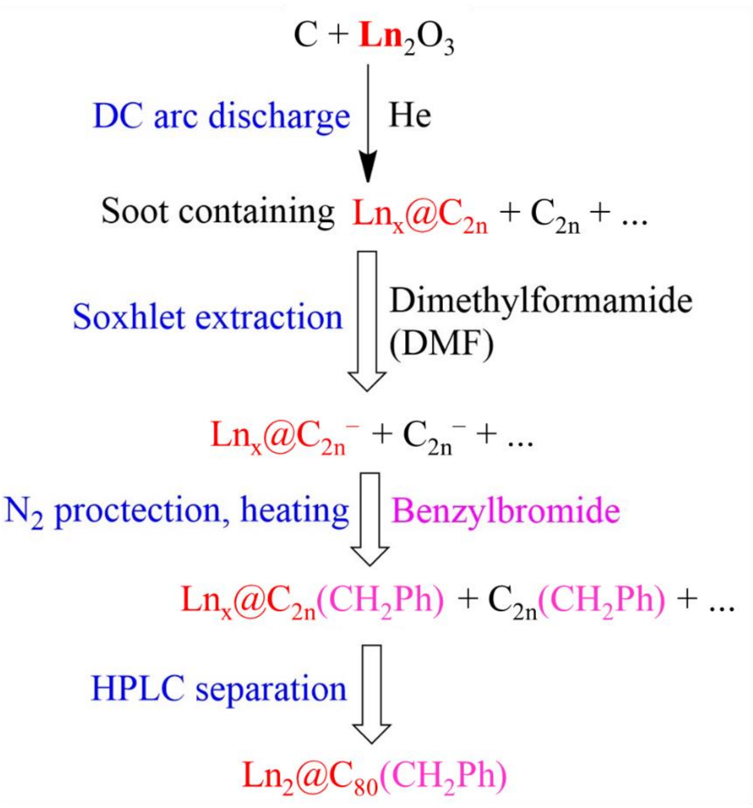

Supplementary Figure 1. The synthesis procedure of the $Ln_2@C_{80}(CH_2Ph)$.

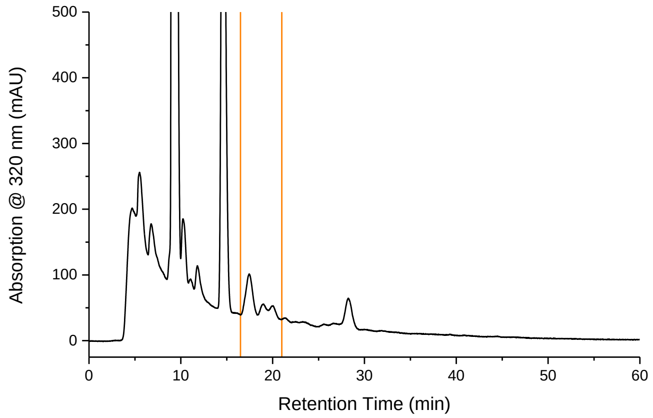

Supplementary Figure 2. The first separation step of {Gd_2} – 2 analytical Buckyprep columns. The fraction between the orange lines contains {Gd_2} and is separated further at the second step.

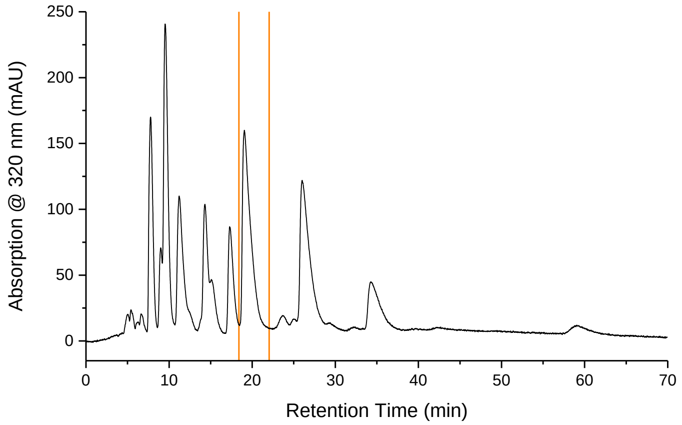

Supplementary Figure 3. The second separation step of {Gd_2} – 2 analytical BuckyPrep-D columns. The fraction between the orange lines contains {Gd_2} and is separated further at the third step

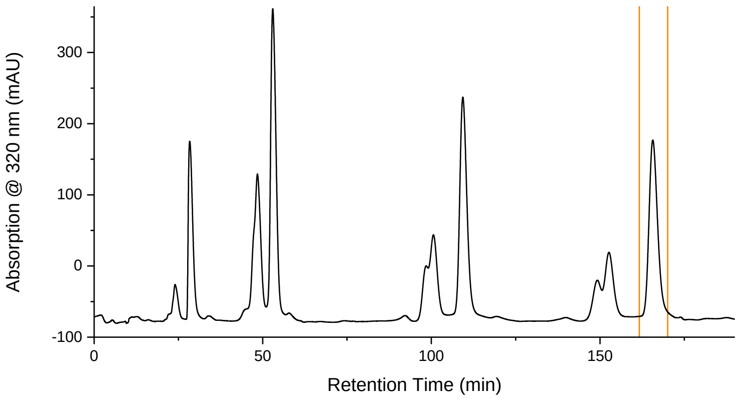

Supplementary Figure 4. The third separation step of {Gd_2} – recycling HPLC with semipreparative BuckyPrep column. The fraction between orange lines is pure {Gd_2}.

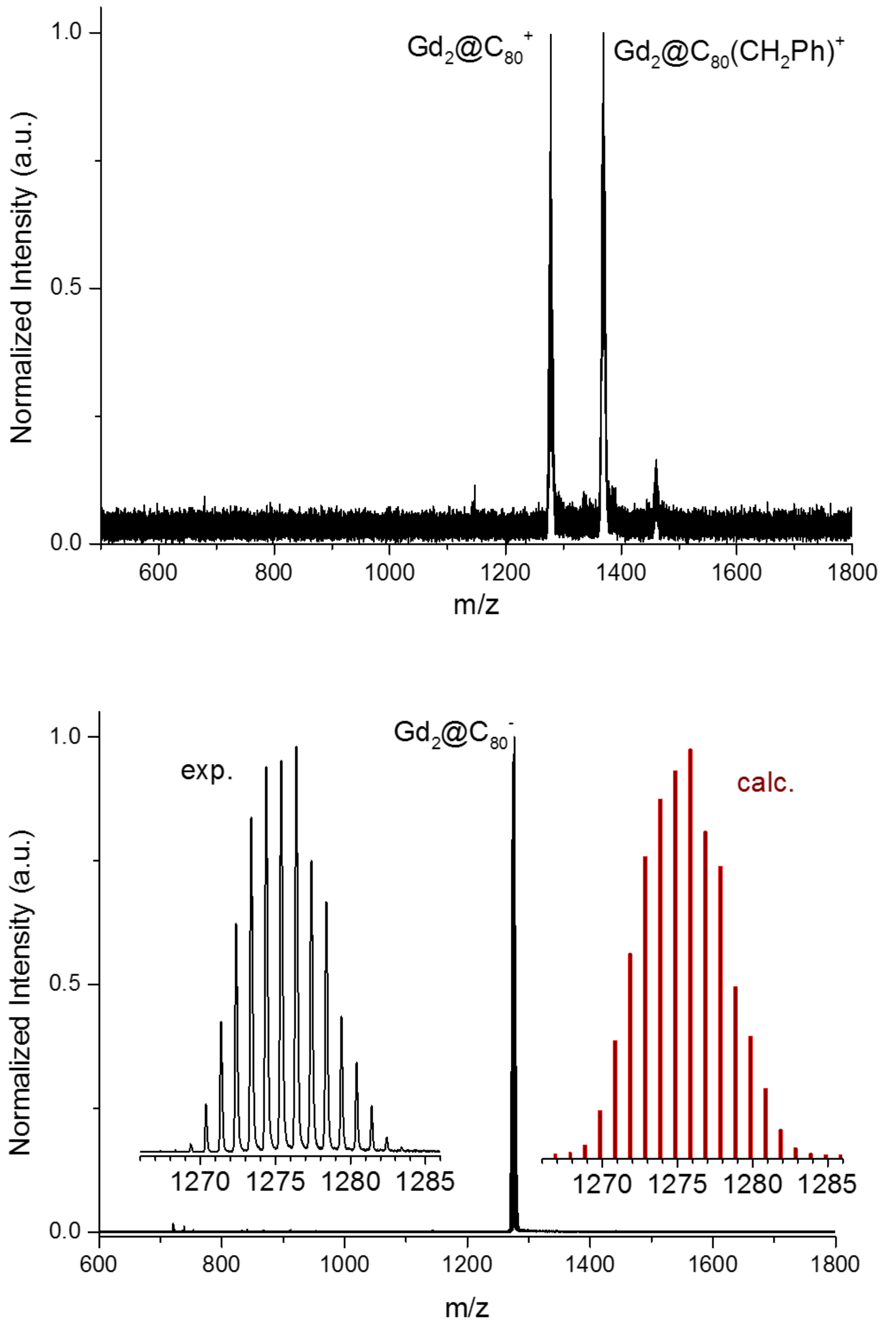

Supplementary Figure 5. Matrix-assisted laser desorption/ionization time-of-flight (MALDI-TOF) mass-spectra of $Gd_2@C_{80}(CH_2Ph)$, linear positive (top) and reflector negative (bottom) ionization modes. Resolution in linear mode is not high enough for analysis of isotopic distribution. In the reflector mode, strong fragmentation does not allow for detection of molecular peak, but spectral resolution is sufficient to prove correct isotopic distribution of the $Gd_2@C_{80}^-$ fragment.

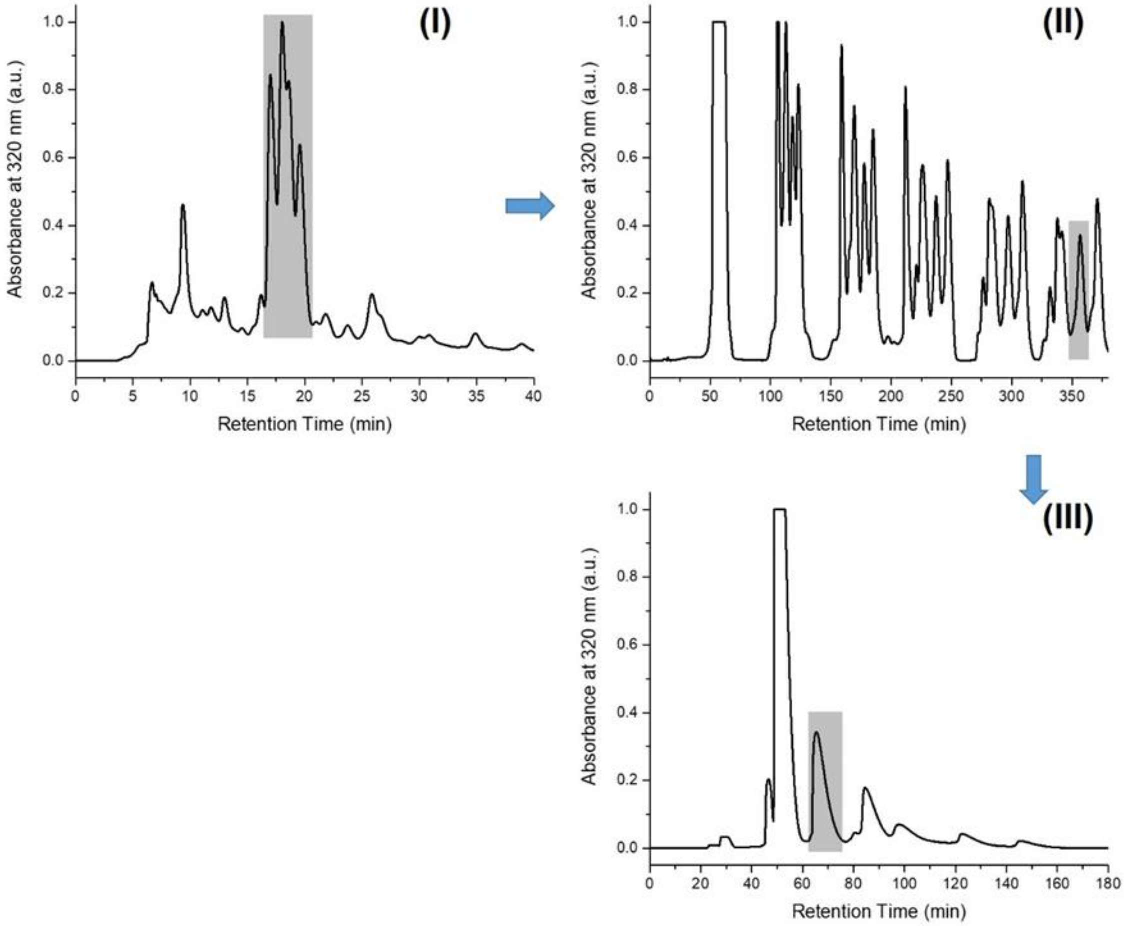

Supplementary Figure 6. Separation of $Tb_2@C_{80}(CH_2Ph)$. Three steps HPLC separation were required to obtain the pure compound. (I) HPLC profile of the mixture of benzyl-derivatized Tb-EMFs. The highlighted fraction contained $Tb_2@C_{80}(CH_2Ph)$ was collected for further separation. HPLC conditions: linear combination of two 4.6 × 250 mm Buckyprep columns; flow rate 1.6 mL/min; injection volume 800 μL; toluene as eluent; 40 °C. (II) Recycling HPLC separation of the fraction collected in the first step. The highlighted fraction was collected for further separation (10 × 250 mm Buckyprep column; flow rate 1.5 mL/min; injection volume 4.5 mL; toluene as eluent). (III) HPLC separation of the fraction collected in the second step. Pure $Tb_2@C_{80}(CH_2Ph)$ was obtained as the highlighted fraction. (10 × 250 mm Buckyprep-D column; flow rate 1.0 mL/min; injection volume 4.5 mL; toluene as eluent).

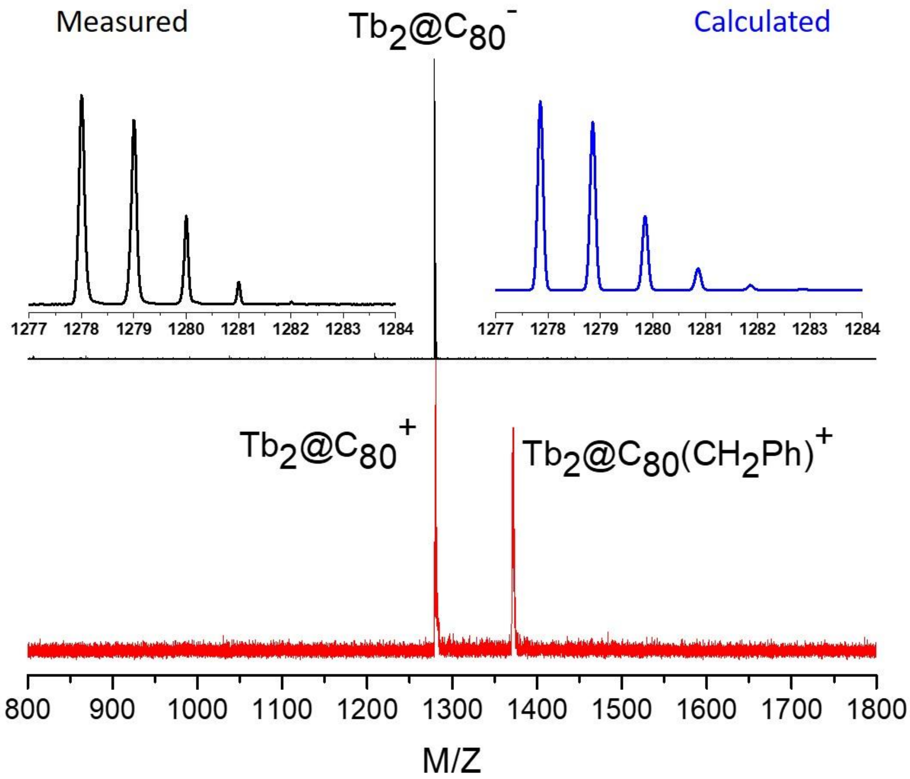

Supplementary Figure 7. Matrix-assisted laser desorption/ionization time-of-flight (MALDI TOF) mass-spectra of $Tb_2@C_{80}(CH_2Ph)$. Linear negative (top) and positive (bottom) ionization modes. Resolution in positive mode is not high enough for analysis of isotopic distribution. In the negative ion mode, strong fragmentation does not allow for detection of molecular peak, but spectral resolution is sufficient to prove correct isotopic distribution of the $Tb_2@C_{80}^-$ fragment.

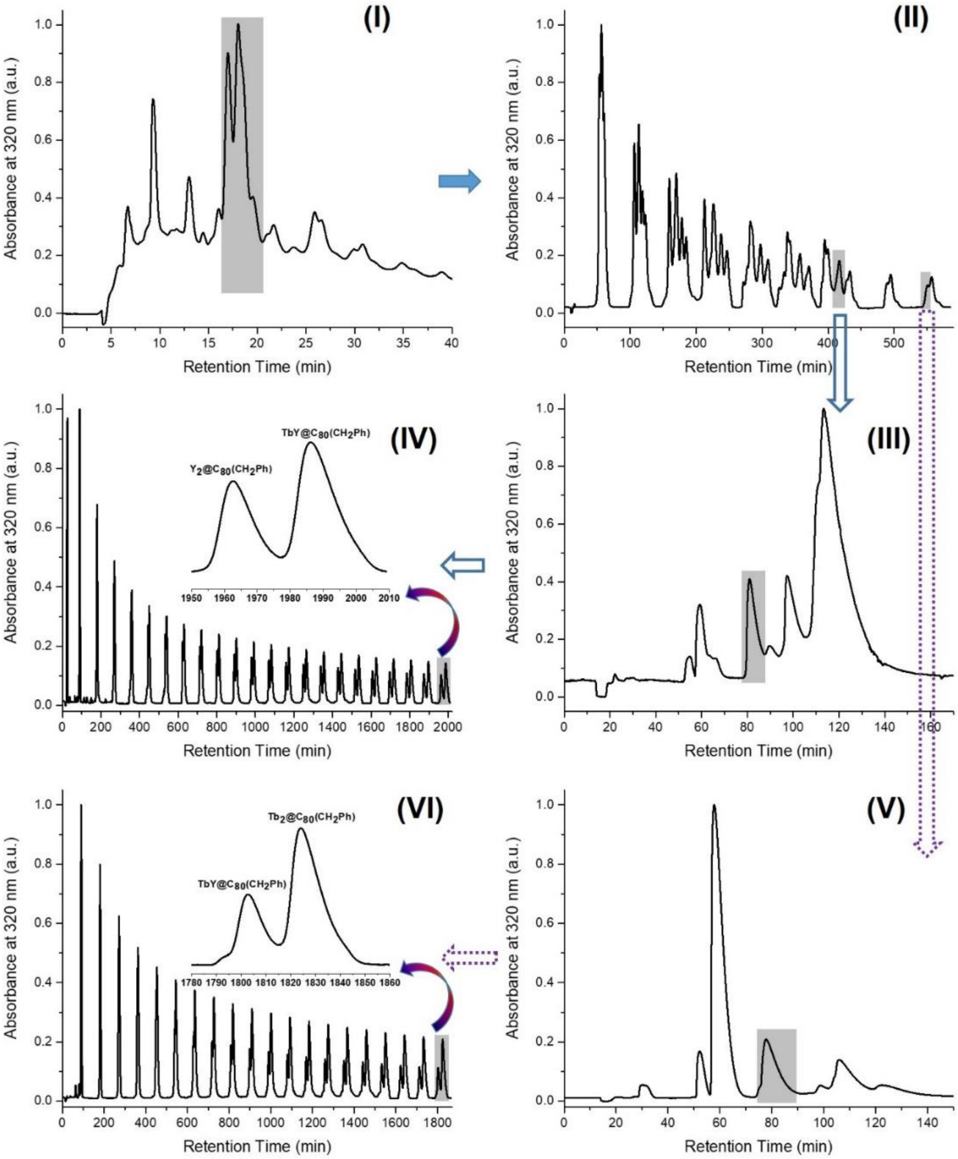

Supplementary Figure 8. Separation of $Tb_xY_{2-x}@C_{80}(CH_2Ph)$. Four HPLC separation steps with toluene as eluent are required to obtain pure compounds. (I) HPLC profile of the mixture of benzyl-derivatized Tb-Y-EMFs (two 4.6 × 250 mm BuckyPrep columns; 1.6 mL min^{-1}). The highlighted fraction contains $Tb_xY_{2-x}@C_{80}(CH_2Ph)$. (II) Recycling HPLC of the fraction collected in the first step (10 × 250 mm Buckyprep column; 1.5 mL min^{-1}). The two highlighted fractions were collected for further separation. (III) HPLC of the first highlighted fraction collected in the second step (10 × 250 mm Buckyprep-D column; 1.0 mL min^{-1}). Highlighted fraction contains $Y_2@C_{80}(CH_2Ph)$ and $TbY@C_{80}(CH_2Ph)$. (IV) Recycling HPLC of the fraction collected in the third step (10 × 250 mm Buckyprep column; 1.0 mL min^{-1}). Pure $Y_2@C_{80}(CH_2Ph)$ and $TbY@C_{80}(CH_2Ph)$ are obtained. (V) HPLC of the second highlighted fraction collected in the second step (10 × 250 mm Buckyprep-D column; 1.0 mL min^{-1}). Highlighted fraction contains $Tb_2@C_{80}(CH_2Ph)$ and $TbY@C_{80}(CH_2Ph)$. (VI) Recycling HPLC of the fraction collected in the last step (10 × 250 mm Buckyprep column; 1.0 mL min^{-1}). Pure $Tb_2@C_{80}(CH_2Ph)$ and $TbY@C_{80}(CH_2Ph)$ are obtained.

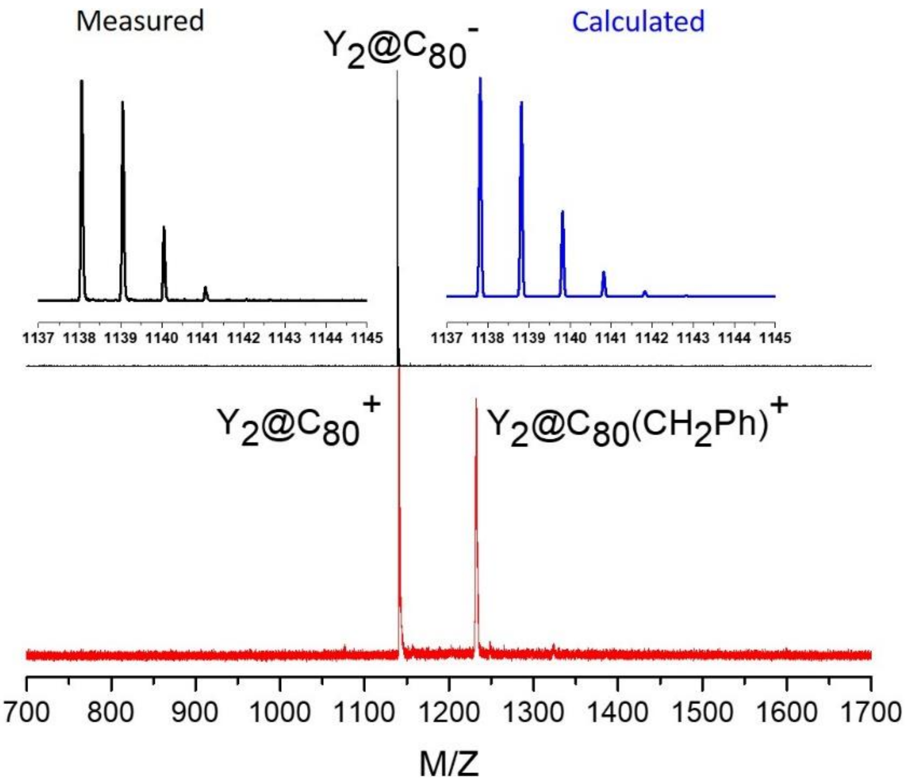

Supplementary Figure 9. Matrix-assisted laser desorption/ionization time-of-flight (MALDI TOF) mass-spectra of $Y_2@C_{80}(CH_2Ph)$. Linear negative (top) and positive (bottom) ionization modes. Resolution in positive mode is not high enough for analysis of isotopic distribution. In the negative ion mode, strong fragmentation does not allow for detection of molecular peak, but spectral resolution is sufficient to prove correct isotopic distribution of the $Y_2@C_{80}^-$ fragment.

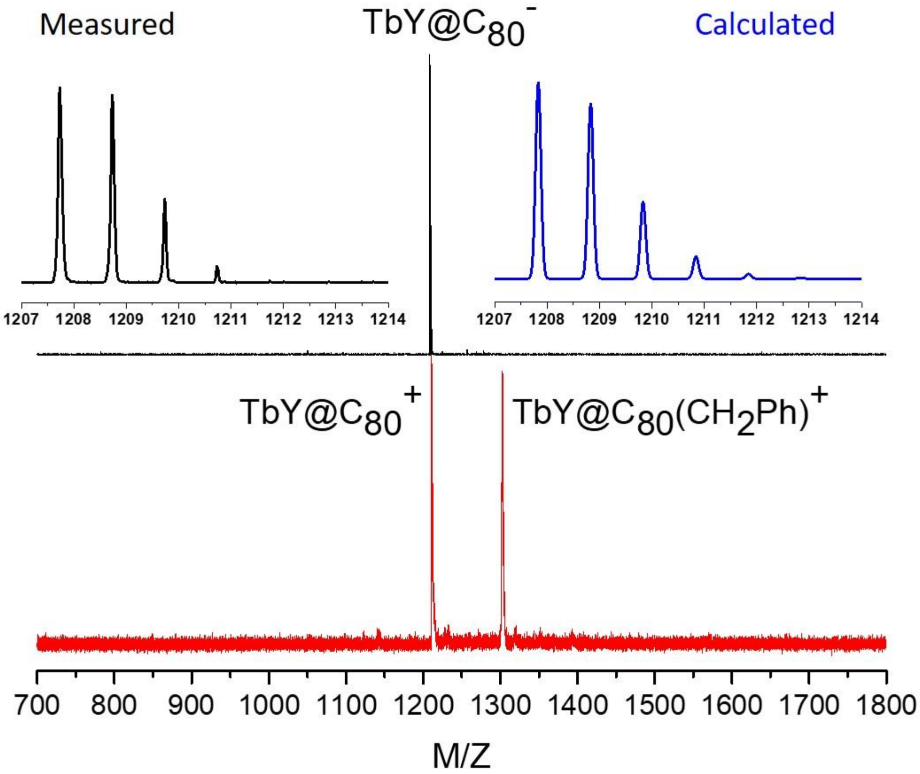

Supplementary Figure 10. Matrix-assisted laser desorption/ionization time-of-flight (MALDI TOF) mass-spectra of TbY@C_{80}(CH_2Ph). Linear negative (top) and positive (bottom) ionization modes. Resolution in positive mode is not high enough for analysis of isotopic distribution. In the negative ion mode, strong fragmentation does not allow for detection of molecular peak, but spectral resolution is sufficient to prove correct isotopic distribution of the TbY@C_{80}^- fragment.

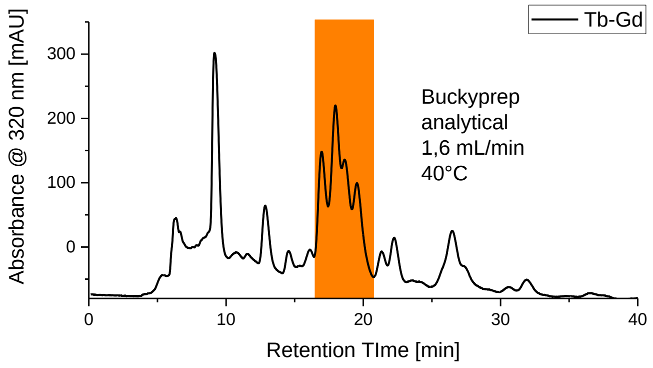

Supplementary Figure 11. First step separation of {TbGd} – 2 analytical Buckyprep columns. The fraction marked with orange contains {TbGd} and is further separated at the second step

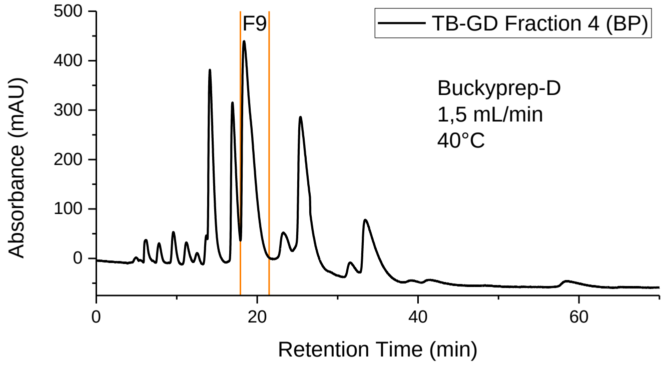

Supplementary Figure 12. Second step separation of {TbGd} – 2 analytical Buclyprep-D columns. Fraction F9 contains {TbGd} and is further separated at the third step

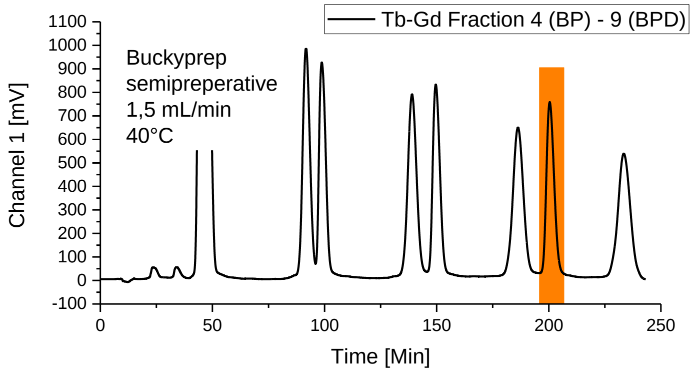

Supplementary Figure 13. Third step separation of {TbGd} – recycling HPLC with semipreparative BuckyPrep column. The fraction marked with orange is a mixture of {Gd_2}, {TbGd}, and {Tb_2}. Longer runs of this fraction with recycling HPLC did not afford further separation into individual compounds.

Mass-spectra and composition of the {TbGd} sample

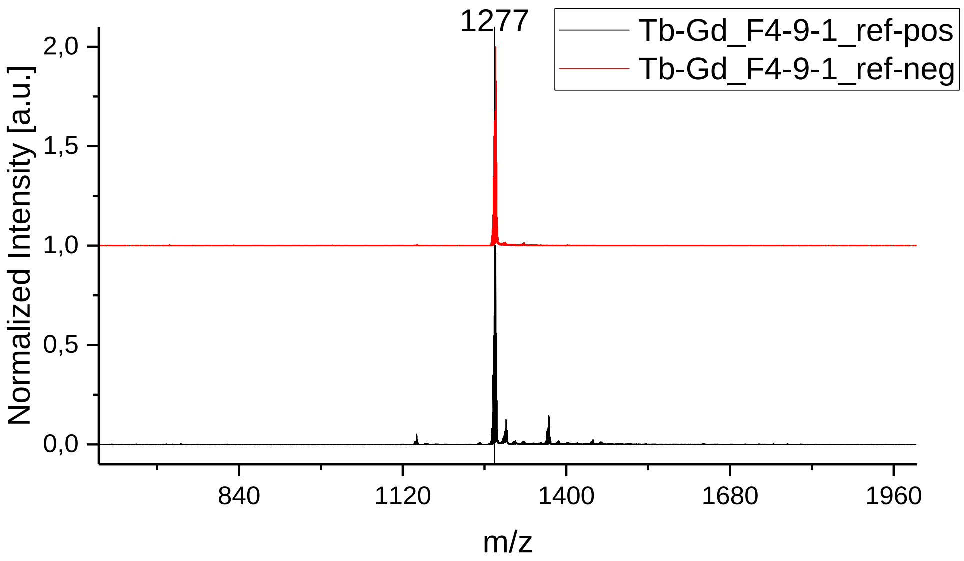

Supplementary Figure 14. MALDI-TOF mass-spectra of the purified "{TbGd}" sample

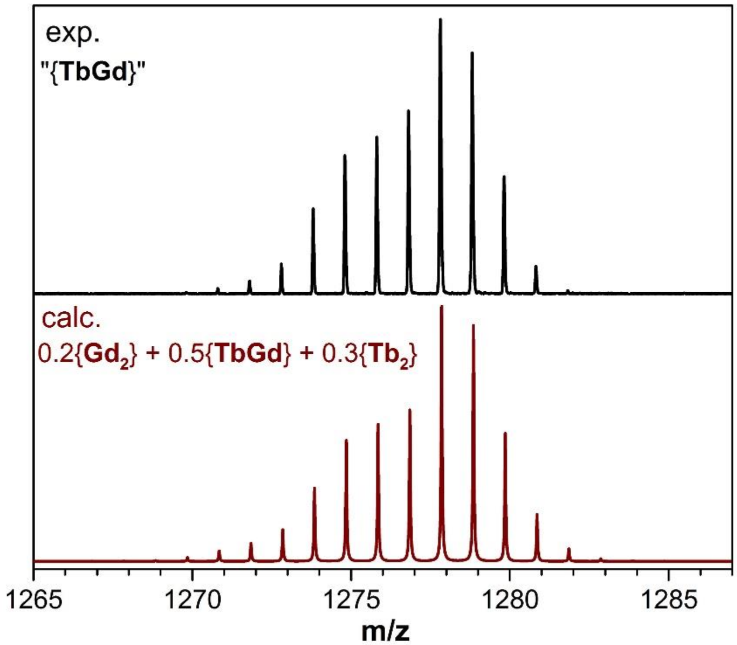

Supplementary Figure 15. MALDI-TOF mass-spectrum of the {TbGd} sample showing experimental isotopic distribution compared to the simulated one for a mixture of 20% {Gd_2}, 30% {Tb_2} and 50% {TbGd}.

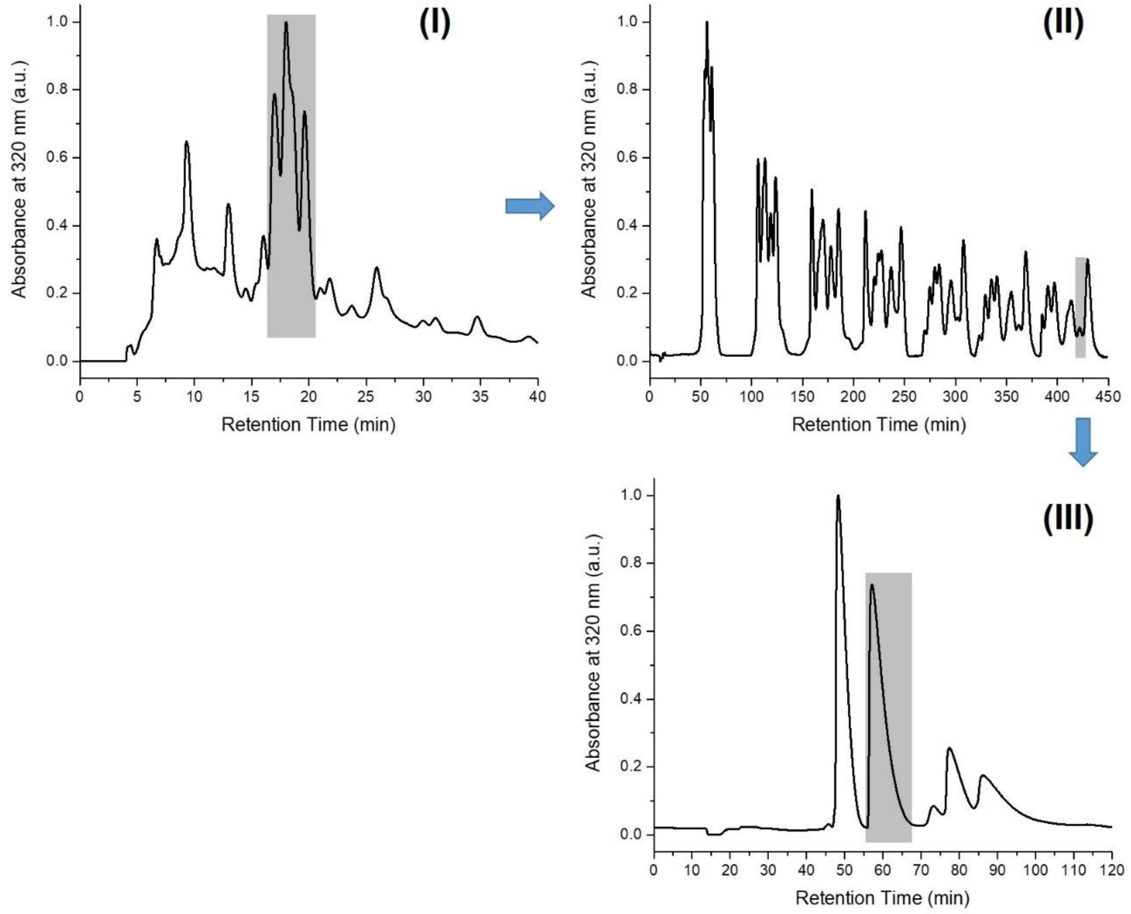

Supplementary Figure 16. Separation of $Ho_2@C_{80}(CH_2Ph)$. Three steps HPLC separation were required to obtain the pure compound (I) HPLC profile of the mixture of benzyl-derivatized Ho-EMFs. The highlighted fraction contained $Ho_2@C_{80}(CH_2Ph)$ was collected for further separation. HPLC conditions: linear combination of two 4.6 × 250 mm Buckyprep columns; flow rate 1.6 mL/min; injection volume 800 μL; toluene as eluent; 40 °C. (II) Recycling HPLC separation of the fraction collected in the first step. The highlighted fraction was collected for further separation (10 × 250 mm Buckyprep column; flow rate 1.5 mL/min; injection volume 4.5 mL; toluene as eluent). (III) HPLC separation of the fraction collected in the second step. Pure $Ho_2@C_{80}(CH_2Ph)$ was obtained as the highlighted fraction. (10 × 250 mm Buckyprep-D column; flow rate 1.0 mL/min; injection volume 4.5 mL; toluene as eluent).

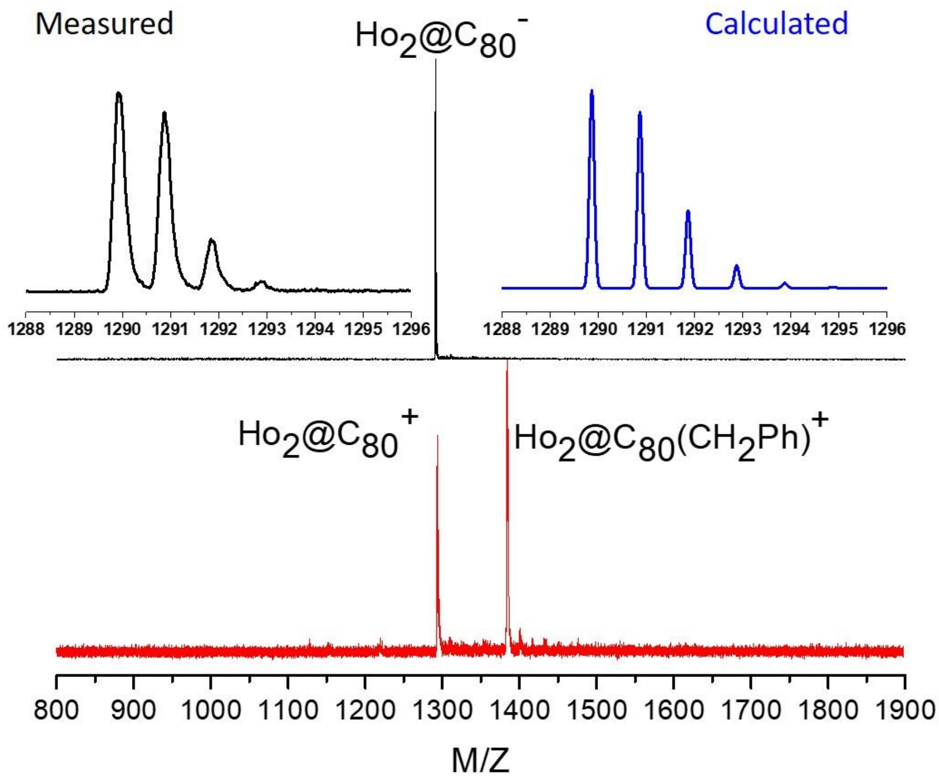

Supplementary Figure 17. Matrix-assisted laser desorption/ionization time-of-flight (MALDI TOF) mass-spectra of $Ho_2@C_{80}(CH_2Ph)$. Linear negative (top) and positive (bottom) ionization modes. Resolution in positive mode is not high enough for analysis of isotopic distribution. In the negative ion mode, strong fragmentation does not allow for detection of molecular peak, but spectral resolution is sufficient to prove correct isotopic distribution of the $Ho_2@C_{80}^-$ fragment.

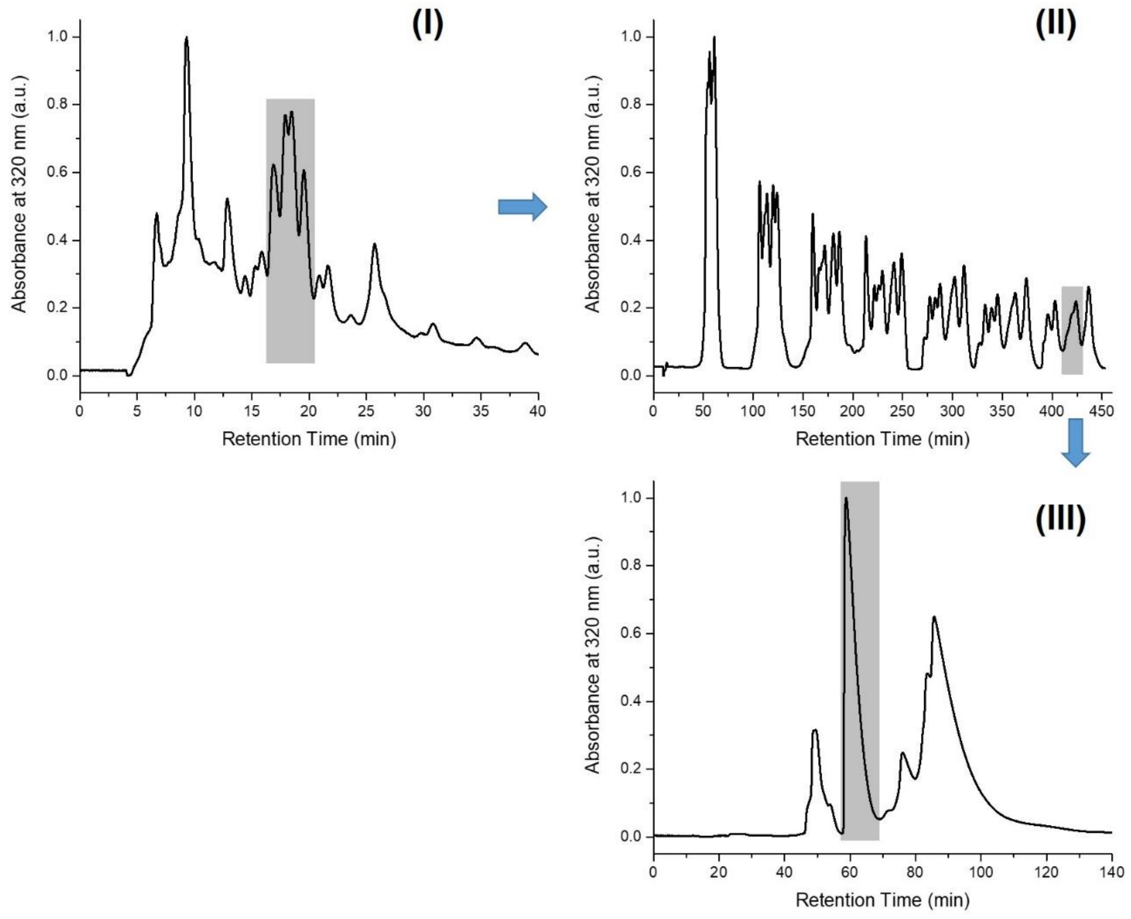

Supplementary Figure 18. Separation of $Er_2@C_{80}(CH_2Ph)$. Three steps HPLC separation were required to obtain the pure compound (I) HPLC profile of the mixture of benzyl-derivatized Er-EMFs. The highlighted fraction contained $Er_2@C_{80}(CH_2Ph)$ was collected for further separation. HPLC conditions: linear combination of two 4.6 × 250 mm Buckyprep columns; flow rate 1.6 mL/min; injection volume 800 μL; toluene as eluent; 40 °C. (II) Recycling HPLC separation of the fraction collected in the first step. The highlighted fraction was collected for further separation (10 × 250 mm Buckyprep column; flow rate 1.5 mL/min; injection volume 4.5 mL; toluene as eluent). (III) HPLC separation of the fraction collected in the second step. Pure $Er_2@C_{80}(CH_2Ph)$ was obtained as the highlighted fraction. (10 × 250 mm Buckyprep-D column; flow rate 1.0 mL/min; injection volume 4.5 mL; toluene as eluent).

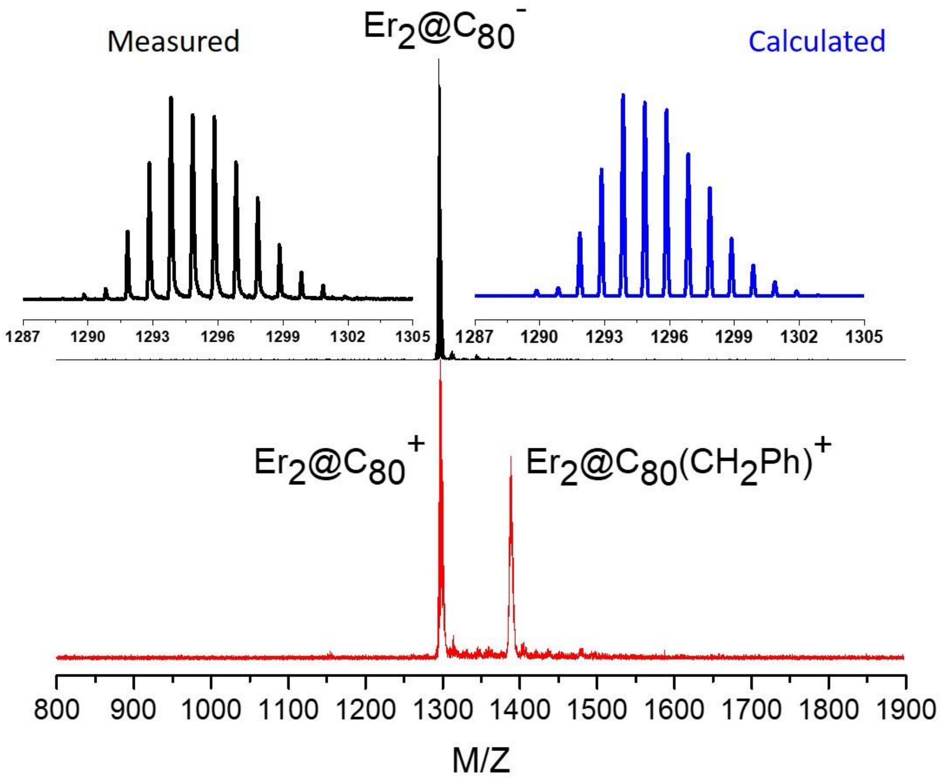

Supplementary Figure 19. Matrix-assisted laser desorption/ionization time-of-flight (MALDI TOF) mass-spectra of $Er_2@C_{80}(CH_2Ph)$. Linear negative (top) and positive (bottom) ionization modes. Resolution in positive mode is not high enough for analysis of isotopic distribution. In the negative ion mode, strong fragmentation does not allow for detection of molecular peak, but spectral resolution is sufficient to prove correct isotopic distribution of the $Er_2@C_{80}^-$ fragment.

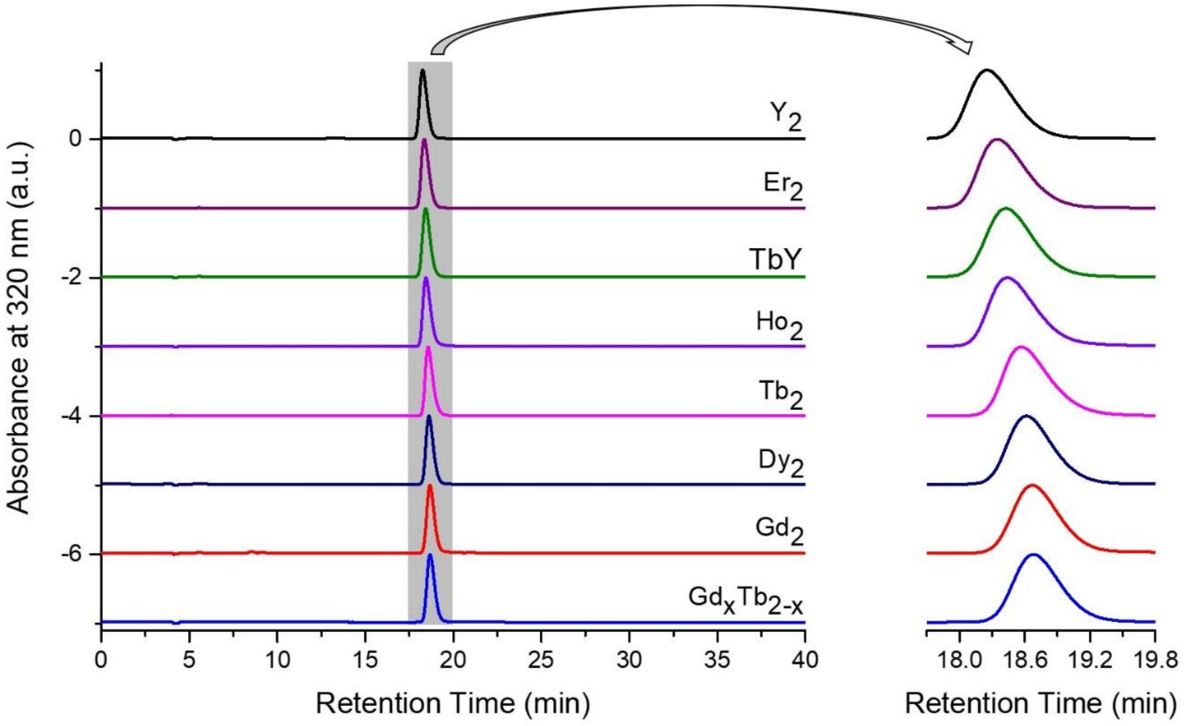

Supplementary Figure 20. HPLC profiles of the purified $M_2@C_{80}(CH_2Ph)$. HPLC conditions: linear combination of two 4.6 × 250 mm Buckyprep columns; flow rate 1.6 mL/min; injection volume 800 μL; toluene as eluent; 40 °C. The identical retention times for $Tb_xGd_{2-x}@C_{80}(CH_2Ph)$ indicates that the separation of these compounds by HPLC is an impossible mission.

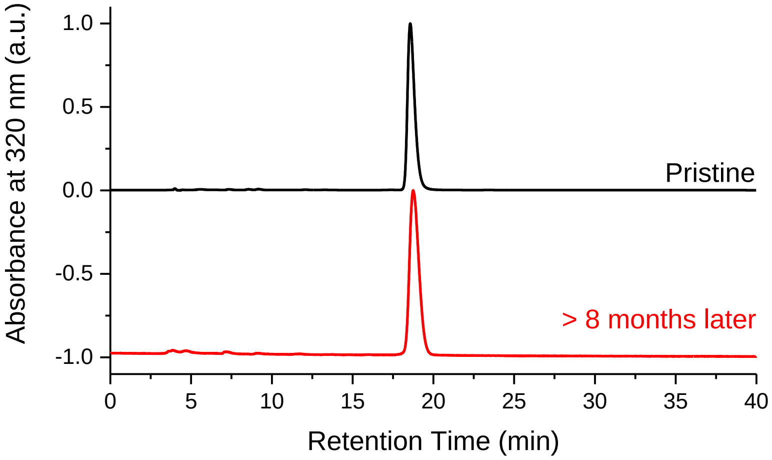

Supplementary Figure 21. Air stability: HPLC trace of the freshly synthesized {Tb_2} and 8 months after the synthesis (during this time period the sample was studied in air by SQUID magnetometry and NMR spectroscopy and stored in solution).

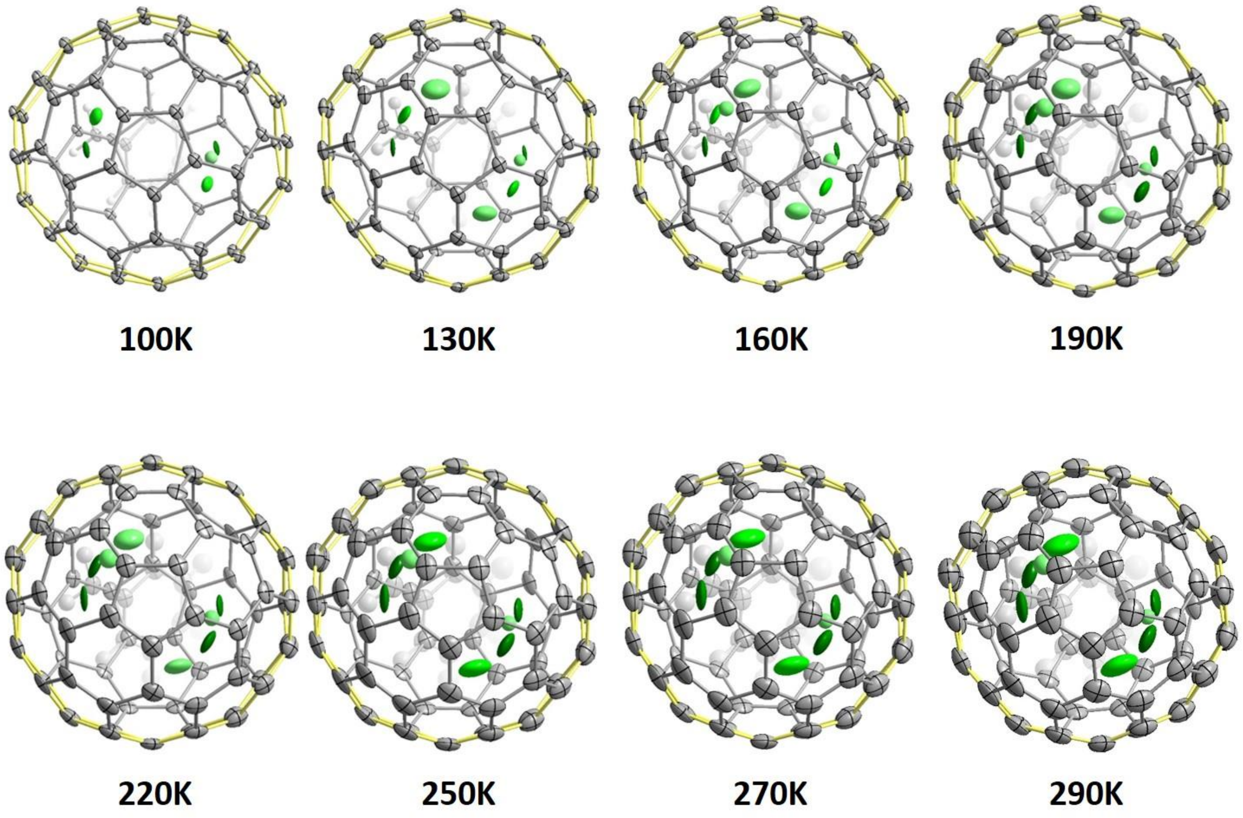

Supplementary Figure 22. Molecular structure of $Dy_2@C_{80}(CH_2Ph)$ determined with single-crystal X-ray diffraction at temperatures from 100K to 290K. The thermal ellipsoids were shown with 50%. The hexagon belt of the C_{80} fullerene cage where the Dy_2 locates is highlighted with yellow bonds. Color code: grey for carbon, green for Dy, the deeper the green color, the higher occupancy of the Dy site.

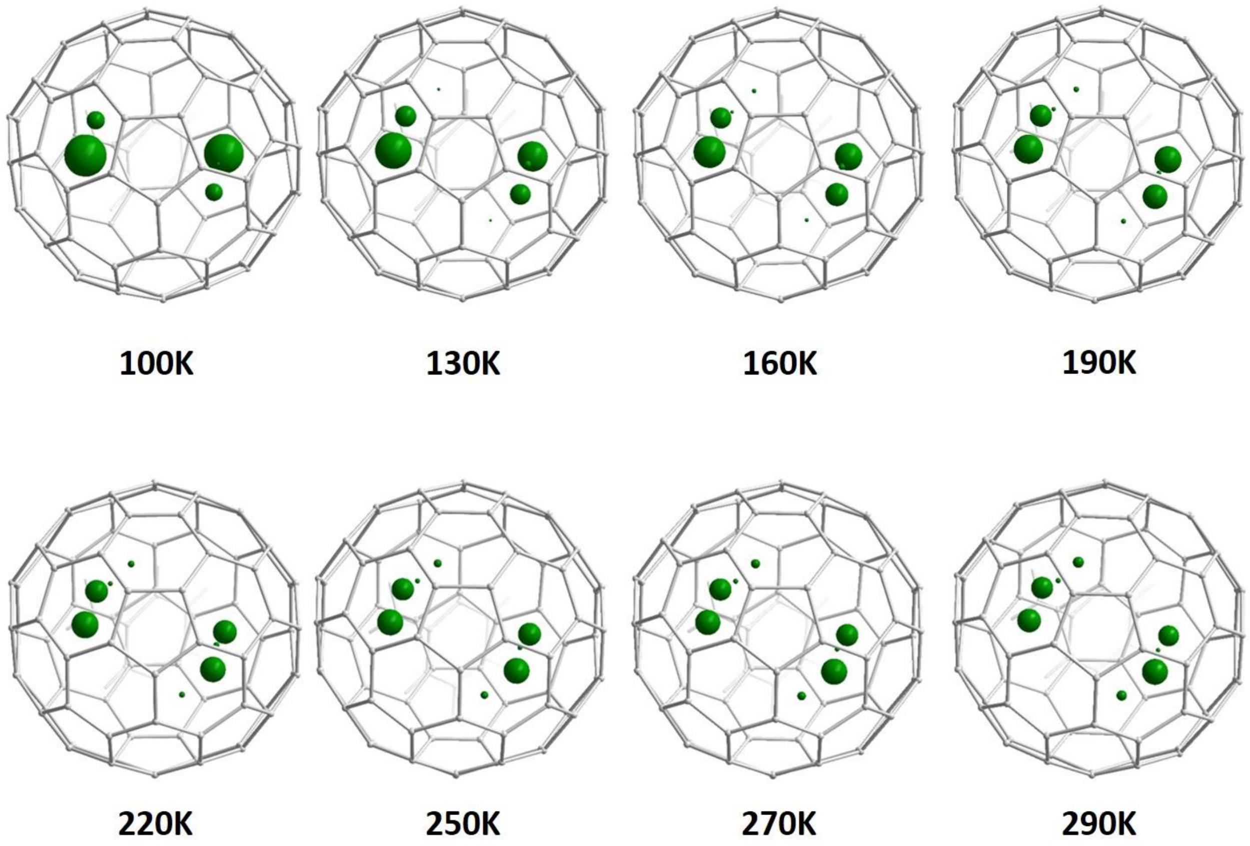

Supplementary Figure 23. Molecular structure of $Dy_2@C_{80}(CH_2Ph)$ determined with single-crystal X-ray diffraction at temperatures from 100K to 290K. The Dy sites are shown as spheres whose radii are scaling proportional to the site occupancy (the bigger the sphere, the higher the occupancy).

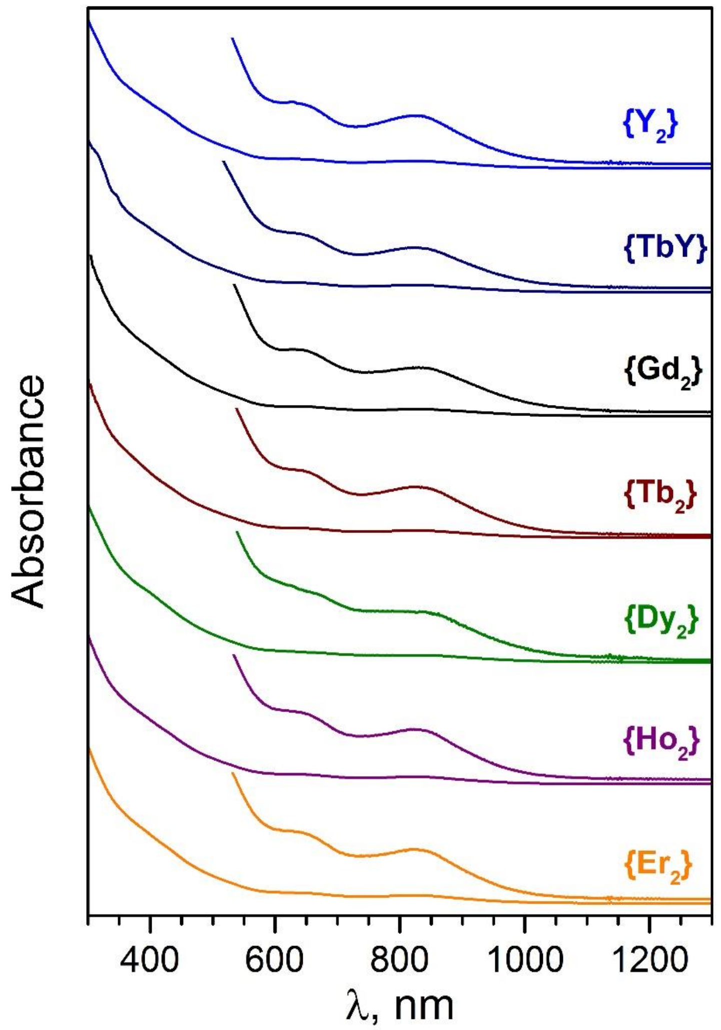

Supplementary Figure 24. UV-vis-NIR absorption spectra of {Ln_2} compounds (room temperature, toluene solution).

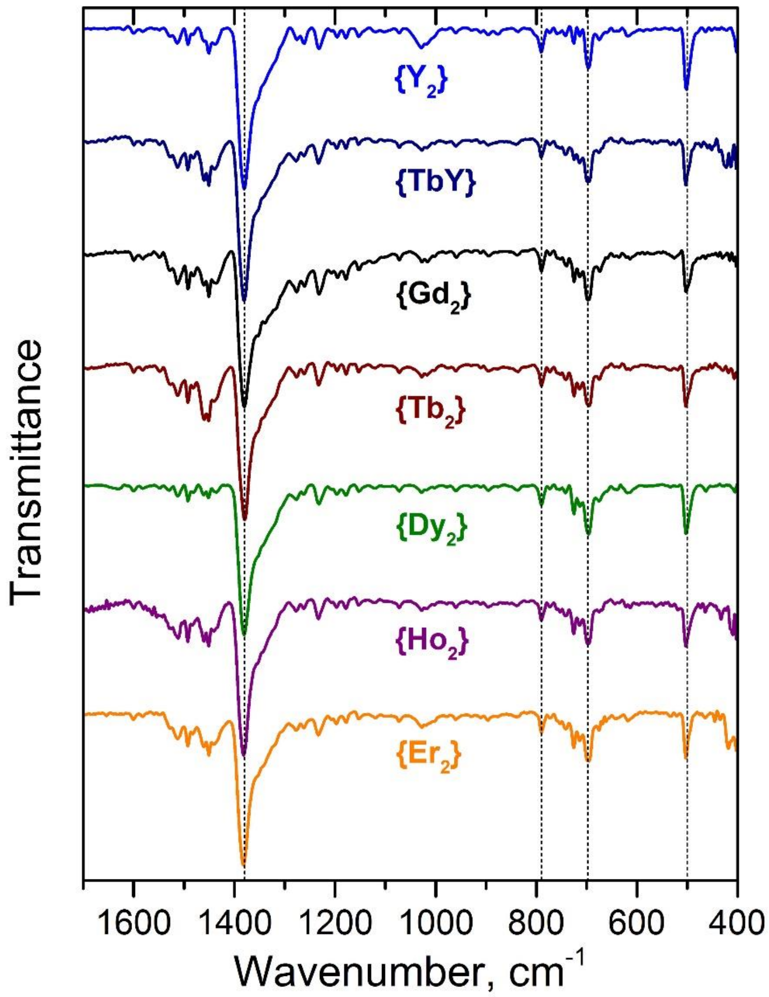

Supplementary Figure 25. FT-IR spectra of {Ln_2} compounds drop-casted on KBr substrates, room temperature.

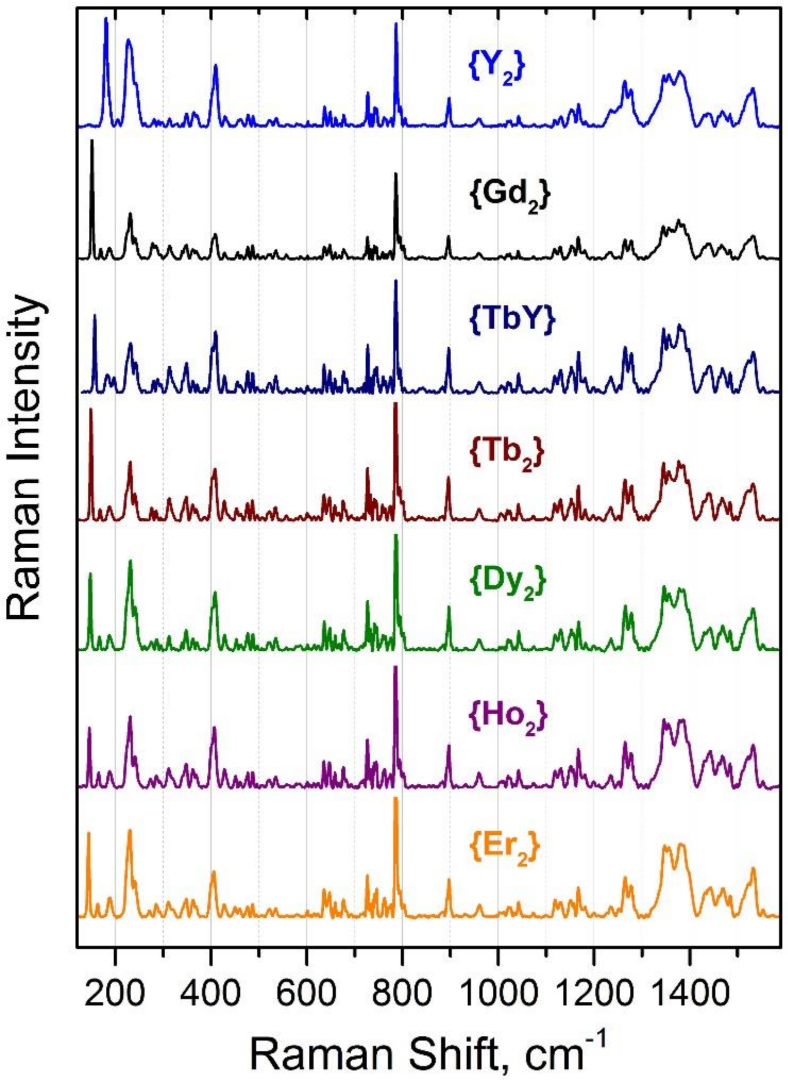

Supplementary Figure 26. Raman spectra of {Ln_2} compounds drop-casted on KBr substrates (the same samples as used for IR measurement). Spectra were recorded at 77 K using excitation line λ = 656 nm of a dye laser.

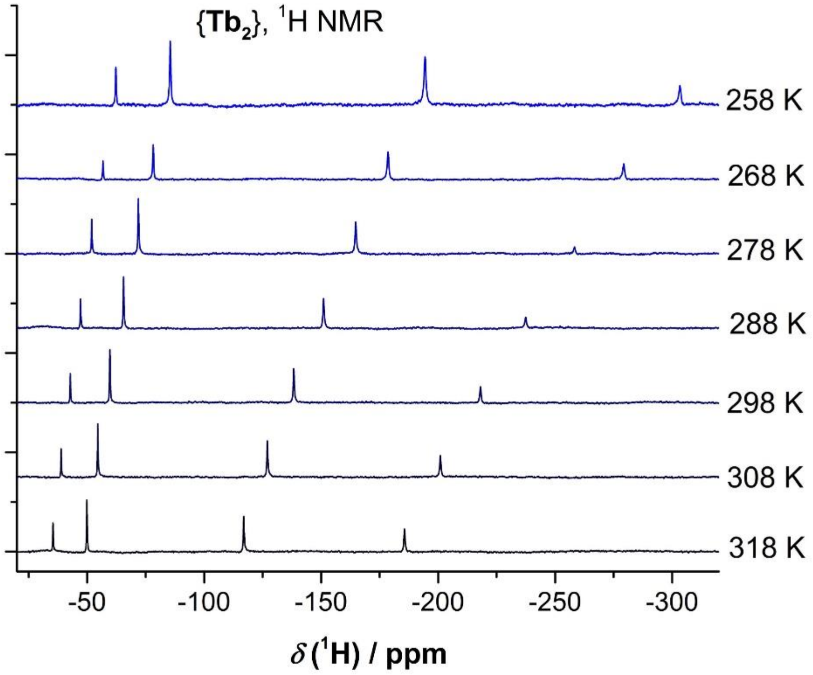

Supplementary Figure 27. ^{1}H VT-NMR spectra of {Tb_2}

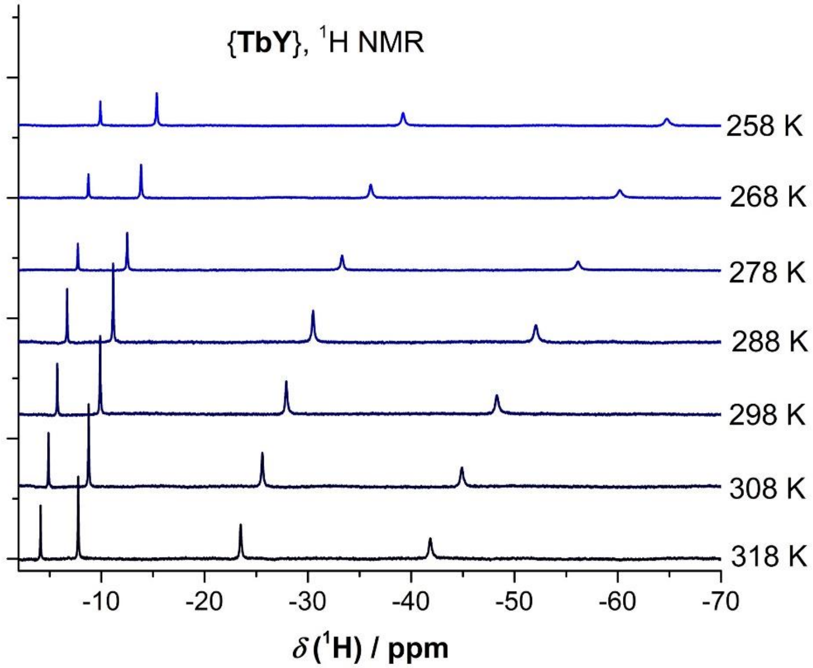

Supplementary Figure 28. ^{1}H VT-NMR spectra of {TbY}

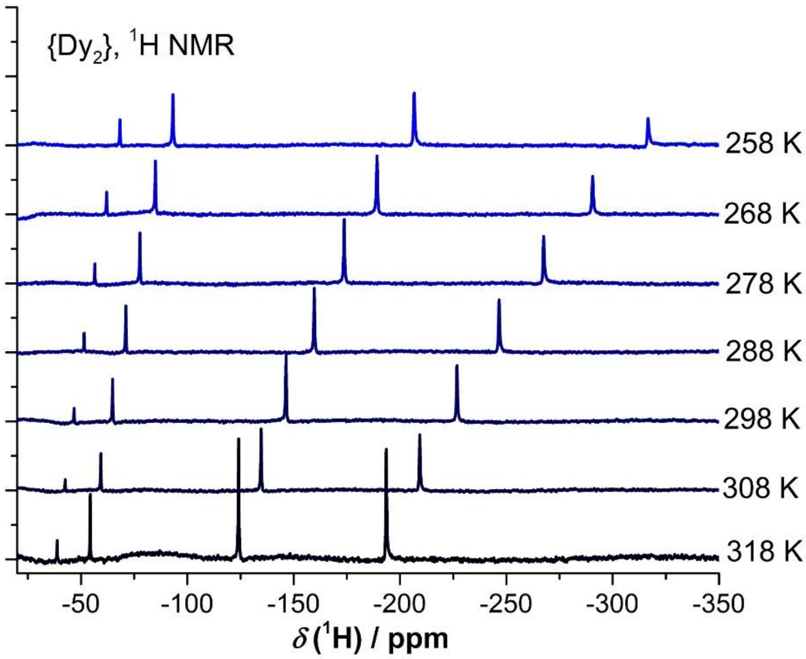

Supplementary Figure 29. ^{1}H VT-NMR spectra of {Dy_2}

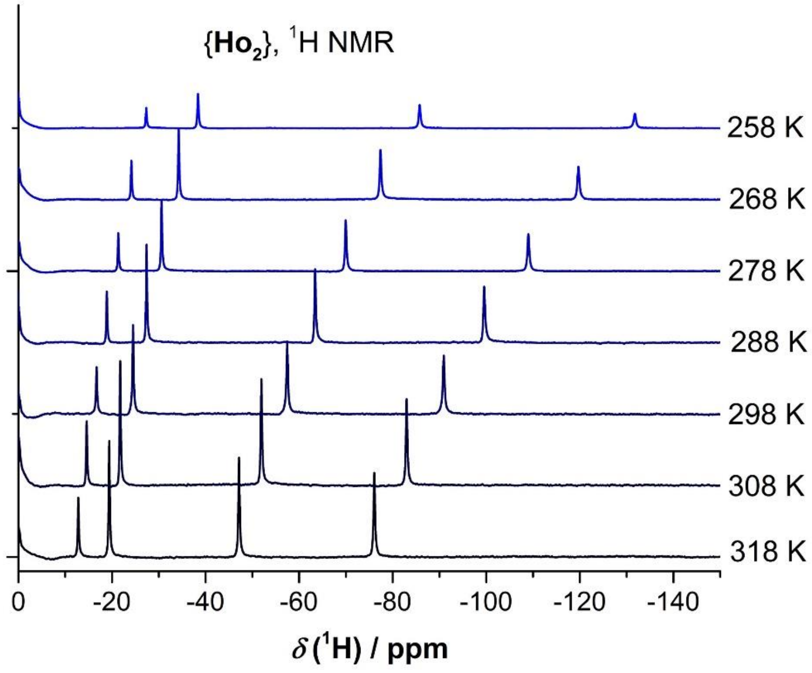

Supplementary Figure 30. ^{1}H VT-NMR spectra of {Ho_2}

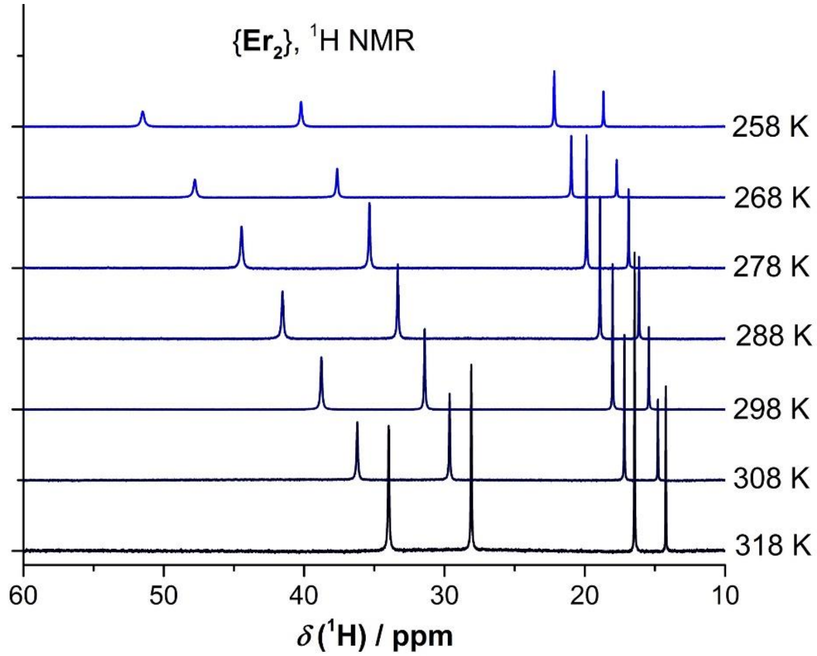

Supplementary Figure 31. ^{1}H VT-NMR spectra of $\{Er_2\}$.

^{13}C NMR spectrum of {Ho_2}

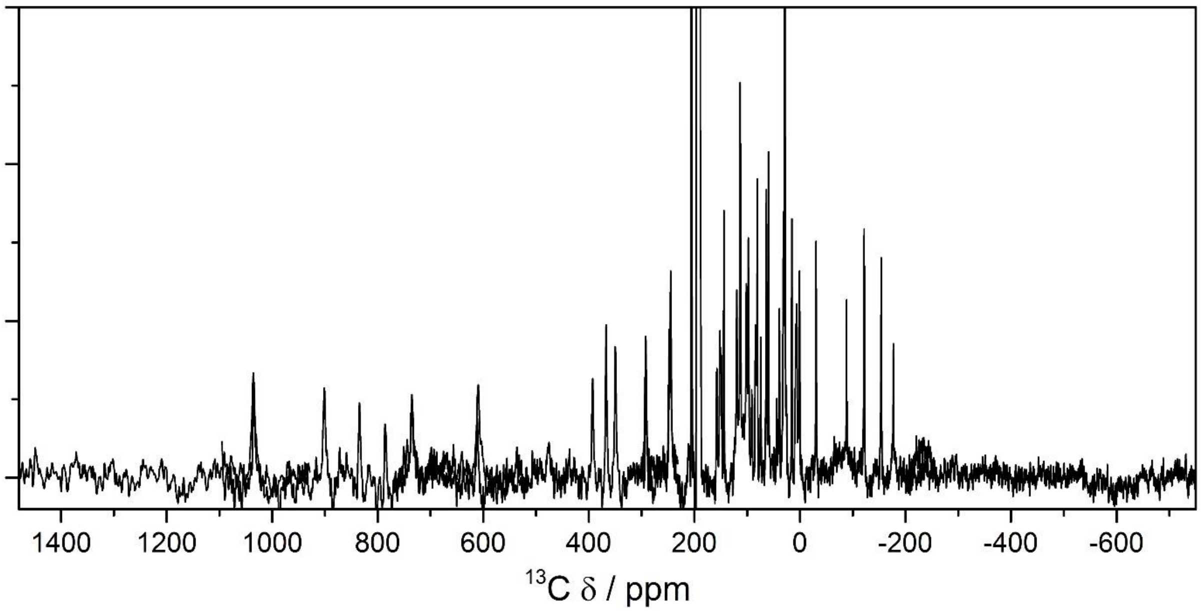

Supplementary Figure 32. ^{13}C NMR spectrum of {Ho_2} measured at 308 K.

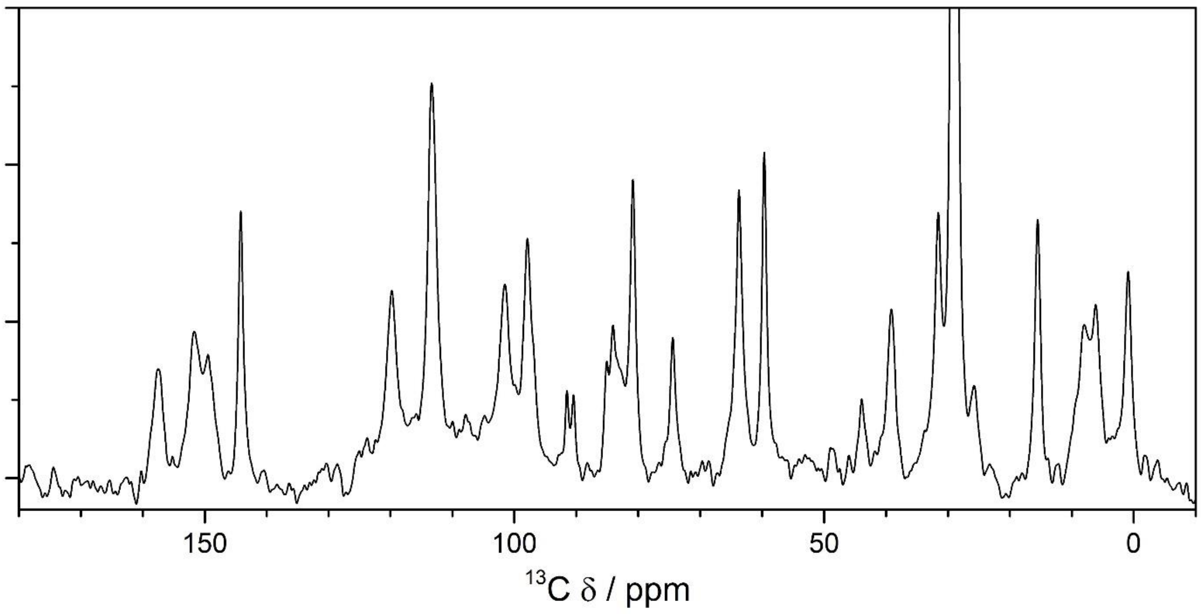

Supplementary Figure 33.^{13}C NMR spectrum of {Ho_2} at 308 K, enhancement of the −15 – 180 ppm range.

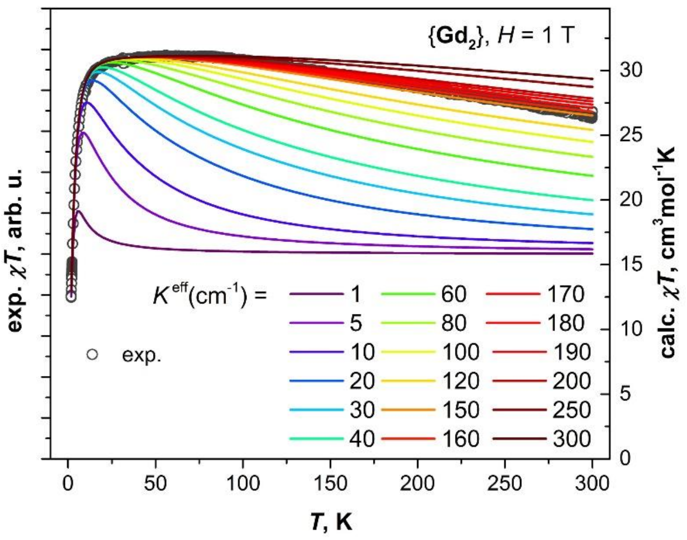

Supplementary Figure 34. Experimental χT curve of {Gd_2} measured in the field of 1 T (dots) and compared to the calculated curves with different values of the exchange parameter K^{eff}. Calculated curves are close to the experimental data for K^{eff} near 160 cm^{-1}.

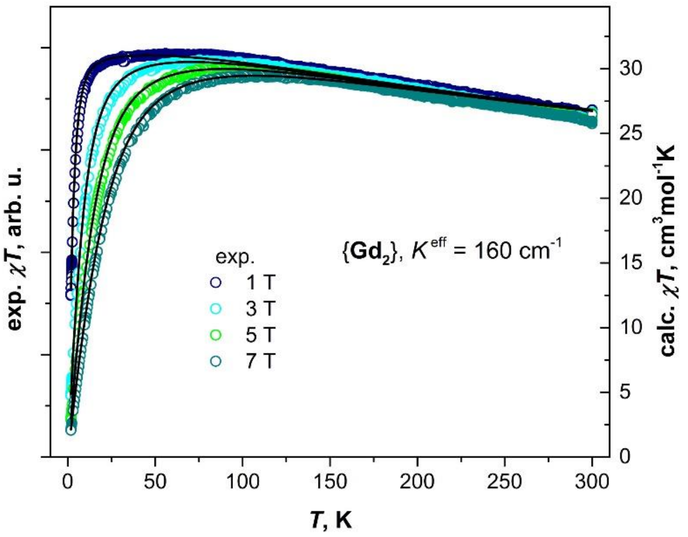

Supplementary Figure 35. Experimental χT of {Gd_2} measured in the fields of 1, 3, 5, and 7 T (dots) compared to the calculated curves with the exchange parameter K^{eff} = 160 cm^{-1} (black lines).

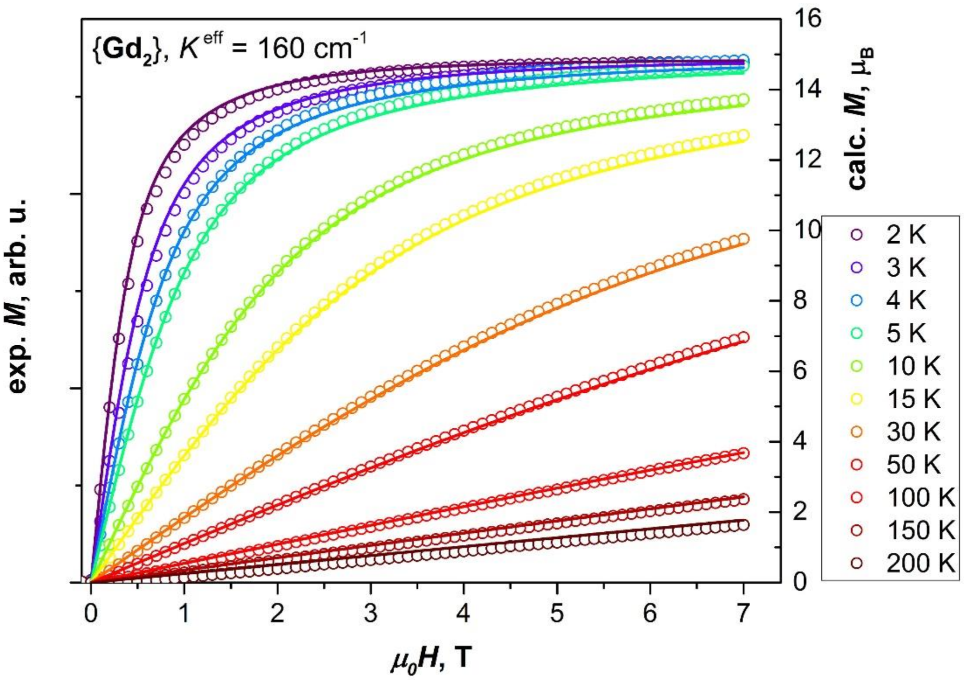

Supplementary Figure 36. Magnetization curves of {Gd_2} (dots) compared to the results of calculations for K^{eff} = 160 cm^{-1} (lines).

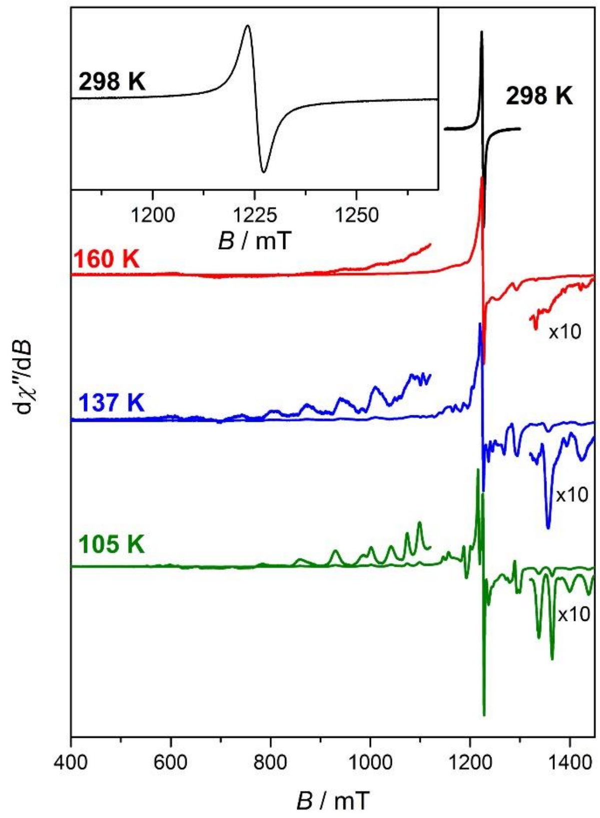

Supplementary Figure 37. Q-band EPR spectra of {Gd_2} in toluene (298 K is liquid solution, 160 – 105 K frozen solution)

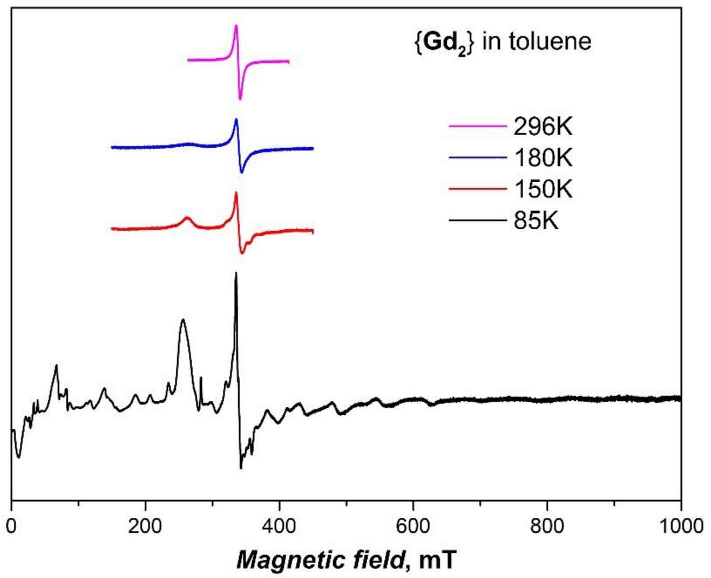

Supplementary Figure 38. X-band EPR spectra of {Gd_2} in toluene

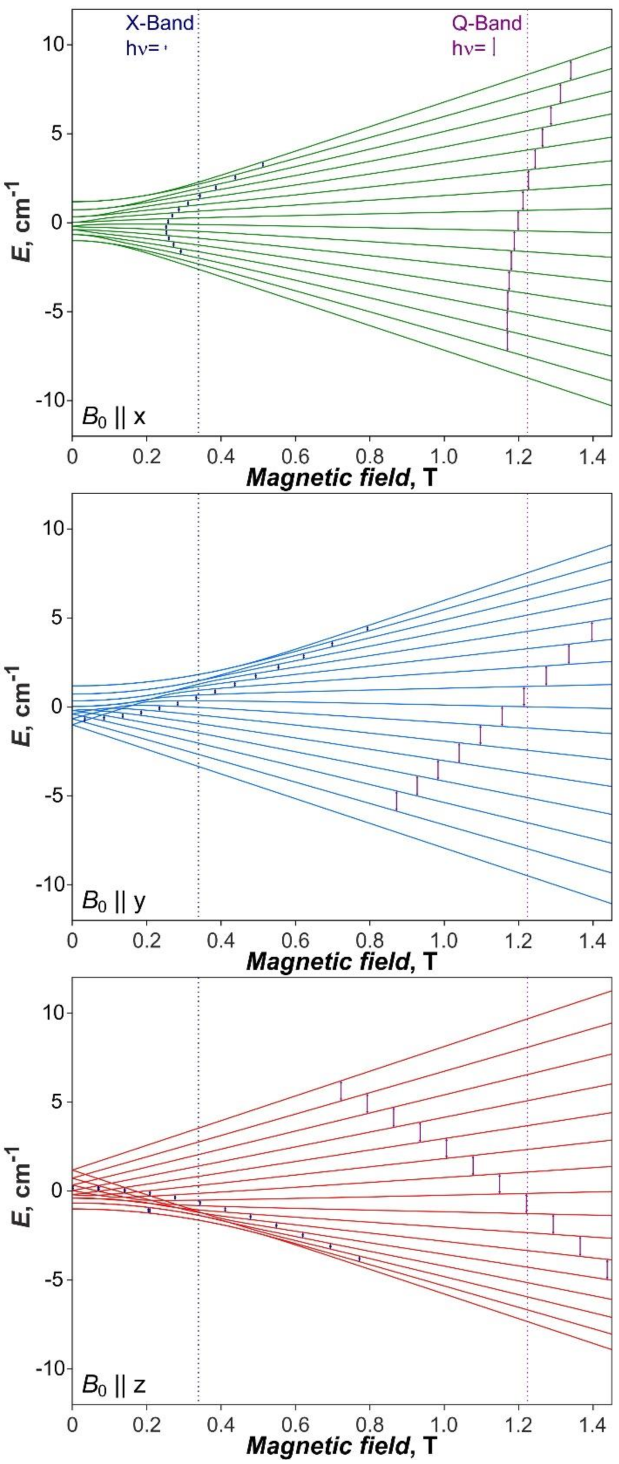

Supplementary Figure 39. Zeeman energy diagrams for {Gd_2} described as a weakly anisotropic giant spin S = 15/2 (Spin Hamiltonian Eq. 5) for the magnetic field applied in three principal directions of the ZFI tensor. Also shown are allowed transitions in X-band and Q-band EPR spectra.

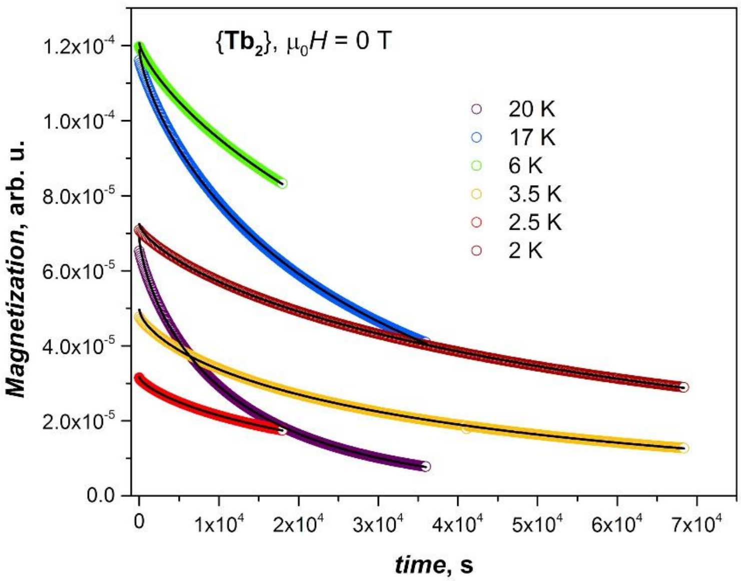

Supplementary Figure 40. Relaxation of magnetization in {Tb_2} measured by first magnetizing the sample in the field of 5 Tesla, then sweeping the field fast to 0 Tesla and measuring the decay (colored dots). The curves are fitted with stretched exponential (black solid lines); see Ref. [2] for a discussion of fitting procedure. Note that the curves were measured for several different samples with slightly different sample amounts. Only selected temperatures are shown.

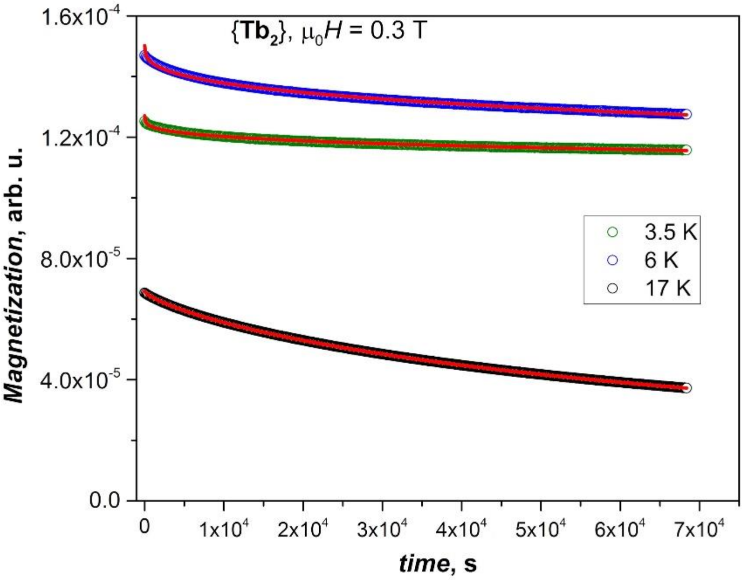

Supplementary Figure 41. Relaxation of magnetization in {Tb_2} measured by first magnetizing the sample at 5 Tesla, then sweeping the field fast to 0.3 Tesla and measuring the decay (colored dots). The curves are fitted with stretched exponential (red solid lines).

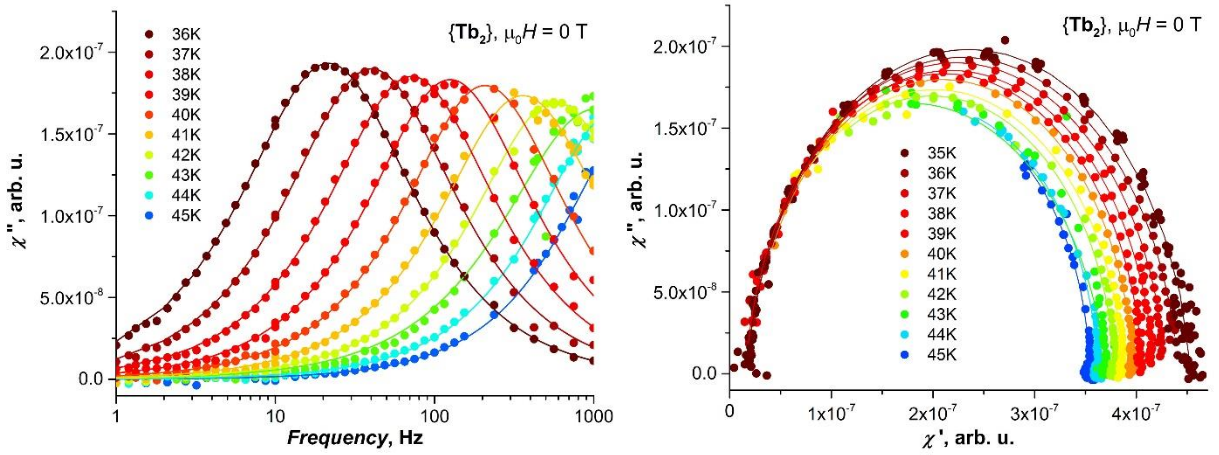

Supplementary Figure 42. Temperature dependence of χ'' of {Tb_2} measured in zero field (left) and corresponding Cole-Cole plots (right). Dots are experimental data, lines are results of the fit with generalized Debye model.

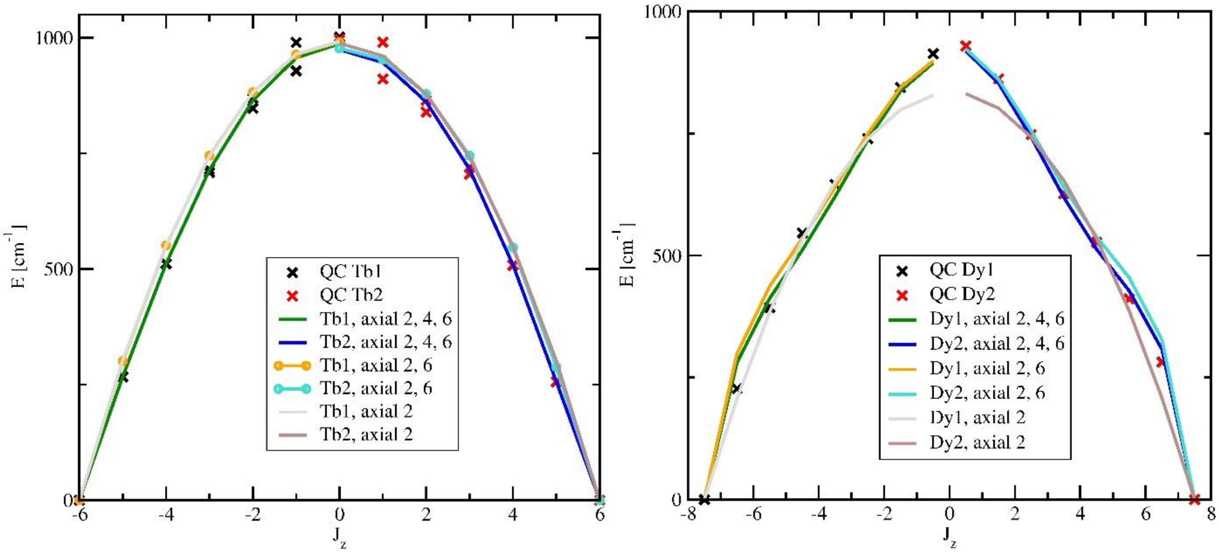

Supplementary Figure 43. Contribution of the second, fourth and sixth order axial terms for Tb^{3+} (left) and Dy^{3+} ions (right) at sites 1 and 2. The symbols represent the *ab initio* energy levels while the curves are expectation values of the LF Hamiltonian, Eq. (4), obtained by considering only the axial terms given in the legends. The data for the Dy ion is taken from Ref. [1]

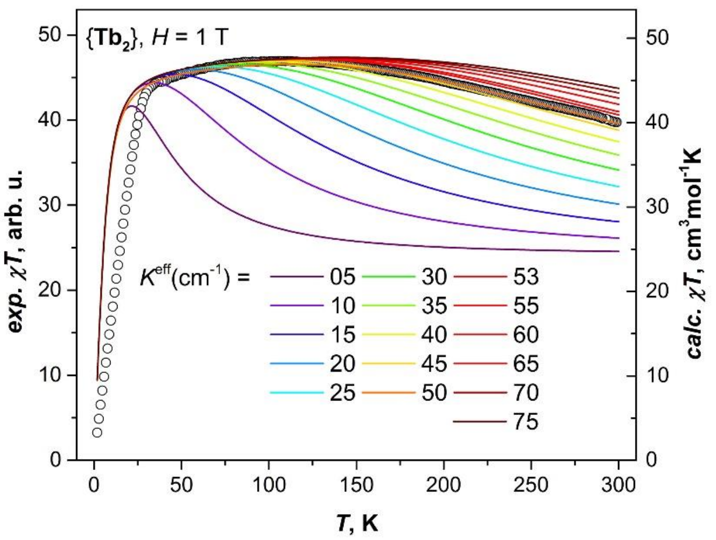

Supplementary Figure 44. Experimental χT curve of {Tb$_2$} measured in the field of 1 T (dots) and compared to the calculated curves with different values of the exchange parameter K^{eff}. Calculated curves are close to the experimental data for K^{eff} in the range from 48 to 53 cm^{-1}. Absolute experimental χT values cannot be determined sufficiently accurate, and hence the experimental curves are scaled to reproduce the calculated data. Note that the experimental χT values measured below the blocking temperature of magnetization (29 K) do not correspond to the equilibrium values.

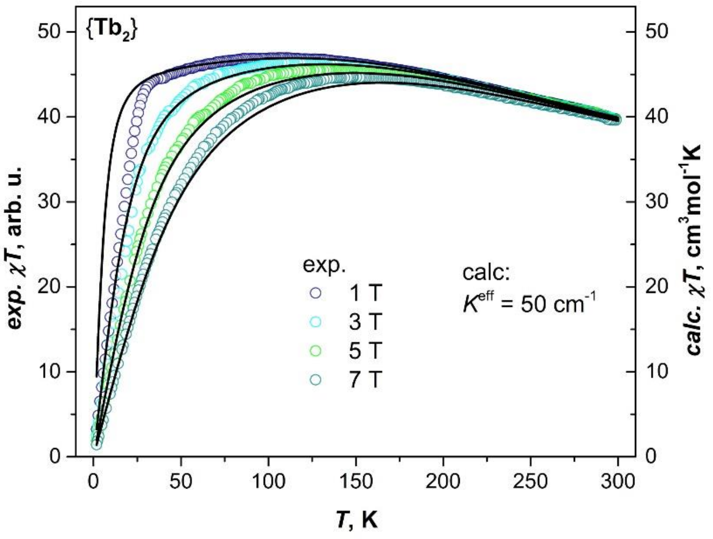

Supplementary Figure 45. Experimental χT of {Tb$_2$} measured in the fields of 1, 3, 5, and 7 T (dots) compared to the calculated curves with the exchange parameter K^{eff} = 50 cm^{-1} (black lines). Experimental χT values are scaled to reproduce the calculated data. Note that the experimental χT values measured below the blocking temperature of magnetization (29 K) do not correspond to the equilibrium values, especially in low fields.

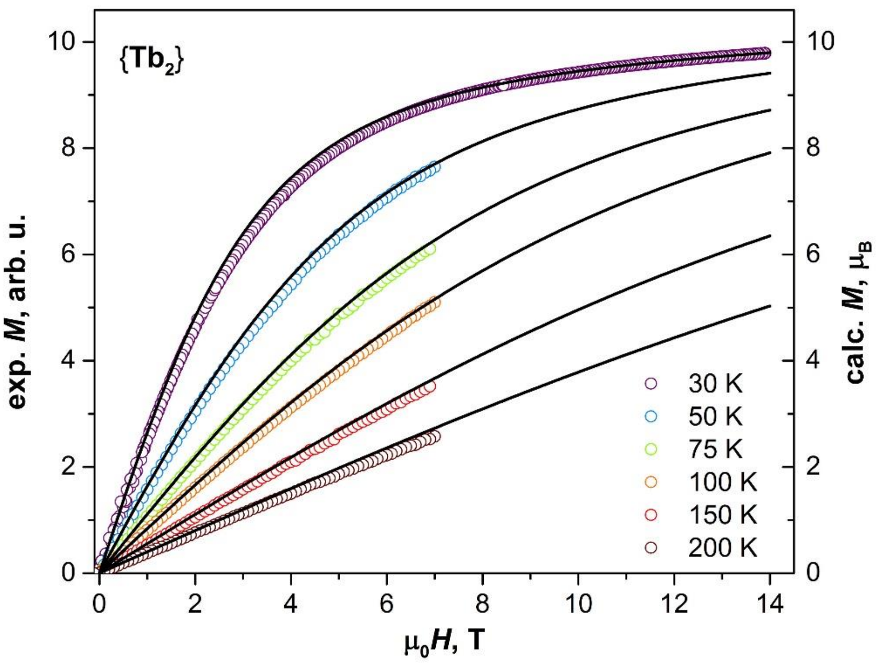

Supplementary Figure 46. Magnetization curves of $\{Tb_2\}$ measured above blocking temperature and compared to the results of calculations for K^{eff} = 50 cm^{-1} (black lines).

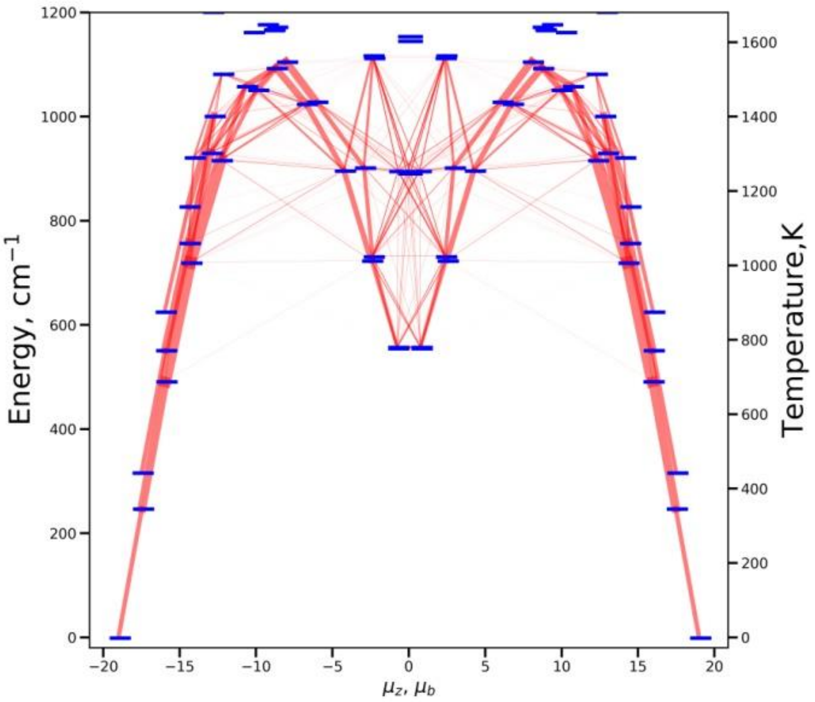

Supplementary Figure 47. The spectrum (low-energy part only) of the effective spin Hamiltonian (Supplementary Equation 1) for {Tb_2} with K^{eff} of 55 cm^{-1}. Transition probabilities for the low-energy range are visualized as lines of different thickness (thicker lines correspond to higher probabilities). The *x*-axis is the projection of magnetic moment upon the main anisotropy axis.

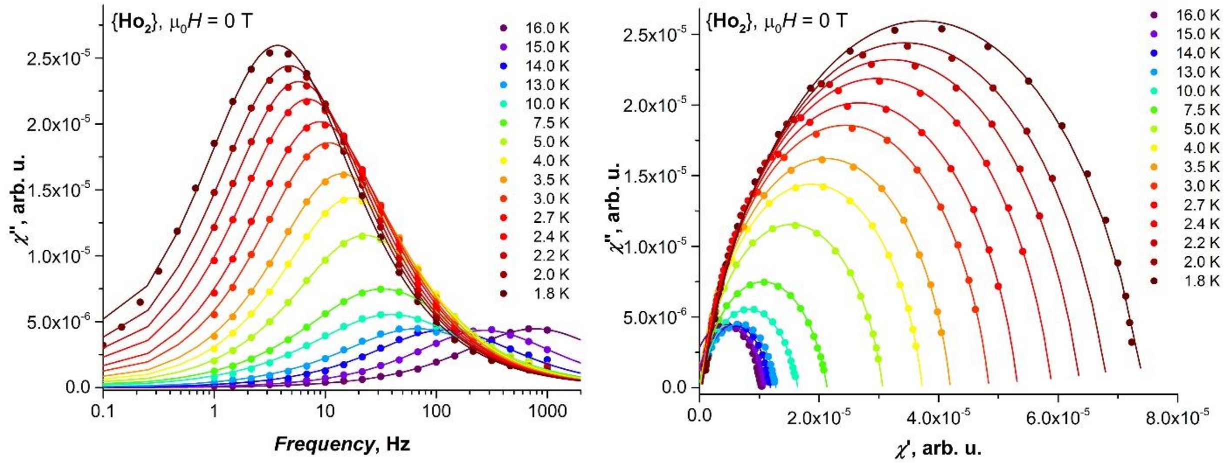

Supplementary Figure 48. Temperature dependence of χ'' of $\{Ho_2\}$ measured in zero field (left) and corresponding Cole-Cole plots (right). Measurements are performed with MPMS-XL system between 1.9 and 16 K. Dots are experimental data, lines are results of the fit with generalized Debye model.

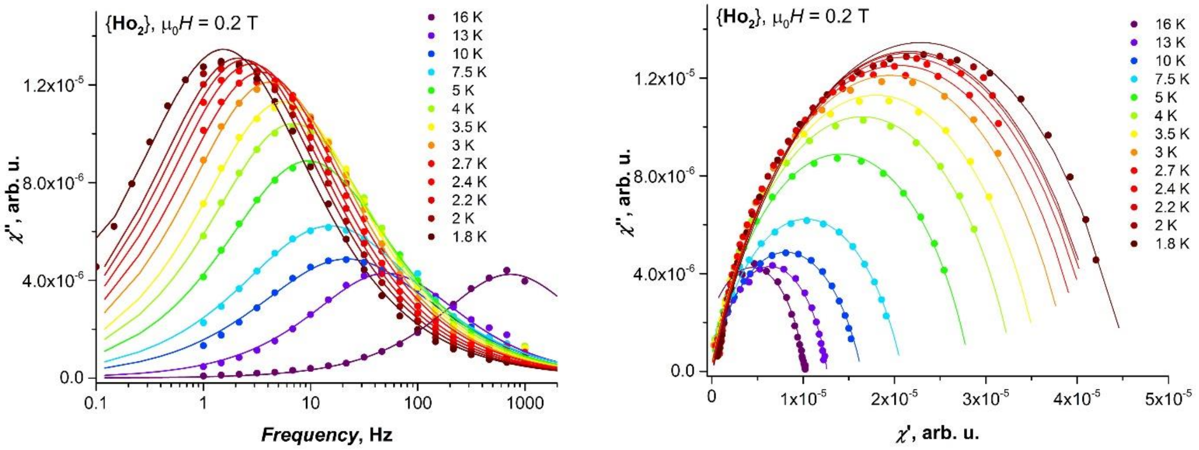

Supplementary Figure 49. Temperature dependence of χ'' of $\{Ho_2\}$ measured in the field of 0.2 T (left) and corresponding Cole-Cole plots (right). Measurements are performed with MPMS-XL system between 1.9 and 16 K. Dots are experimental data, lines are results of the fit with generalized Debye model.

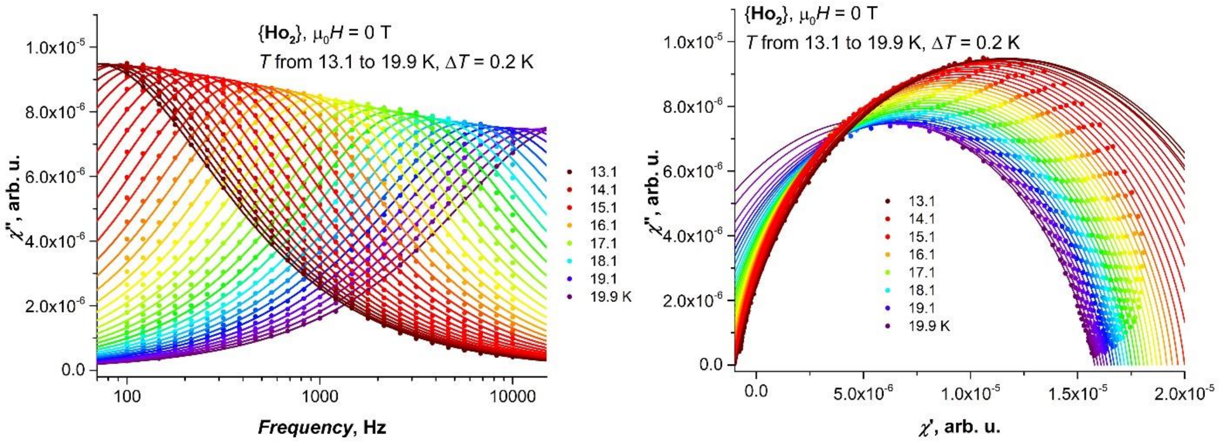

Supplementary Figure 50. Temperature dependence of χ'' of {Ho_2} measured in zero field (left) and corresponding Cole-Cole plots (right). Measurements are performed with PPMS system between 13.1 and 19.9 K. Dots are experimental data, lines are results of the fit with generalized Debye model.

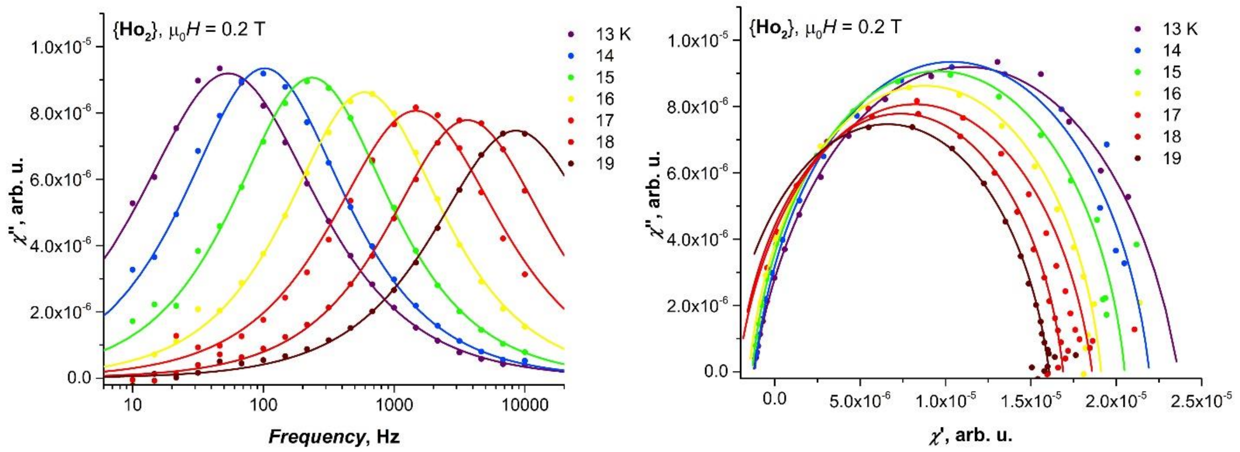

Supplementary Figure 51. Temperature dependence of χ'' of {Ho_2} measured in the field of 0.2 T (left) and corresponding Cole-Cole plots (right). Measurements are performed with PPMS system between 13 and 19 K. Dots are experimental data, lines are results of the fit with generalized Debye model.

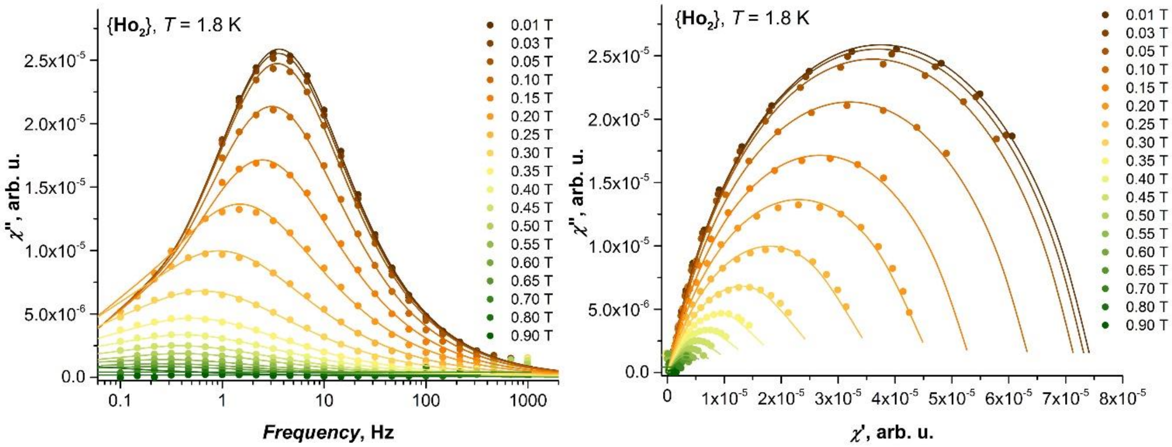

Supplementary Figure 52. Field dependence of χ'' of $\{Ho_2\}$ measured at 1.8 K (left) and corresponding Cole-Cole plots (right). Measurements are performed with MPMS system. Dots are experimental data, lines are results of the fit with generalized Debye model.

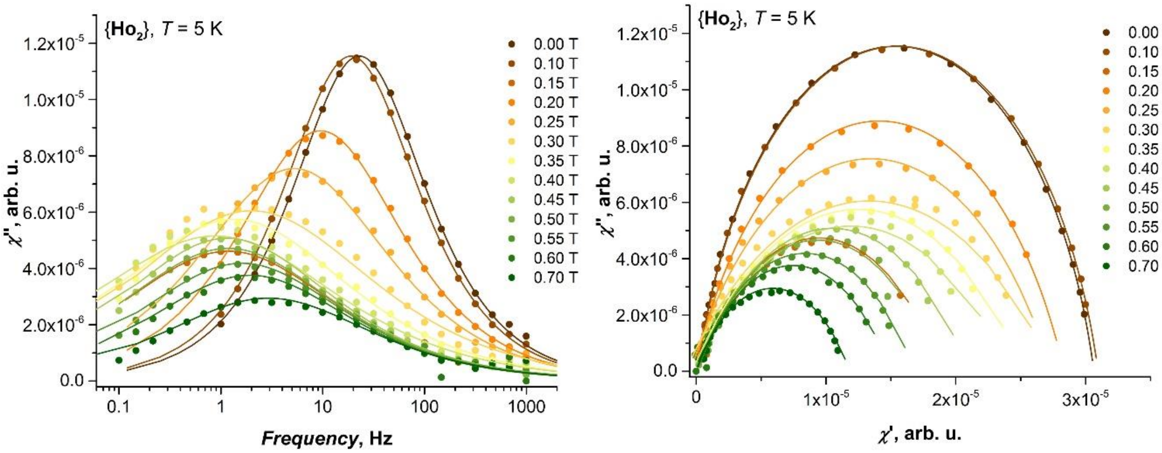

Supplementary Figure 53. Field dependence of χ'' of $\{Ho_2\}$ measured at 5 K (left) and corresponding Cole-Cole plots (right). Measurements are performed with MPMS system. Dots are experimental data, lines are results of the fit with generalized Debye model.

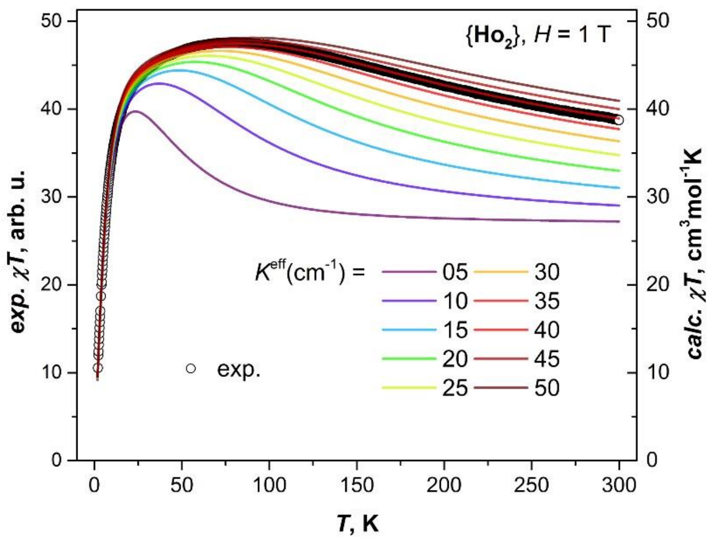

Supplementary Figure 54. Experimental χT curve of {Ho_2} measured in the field of 1 T (dots) and compared to the calculated curves with different values of the exchange parameter K^{eff}. Calculated curves are close to the experimental data for K^{eff} of 40 cm^{-1}. Absolute experimental χT values are not accurate enough and are scaled to reproduce the calculated data.

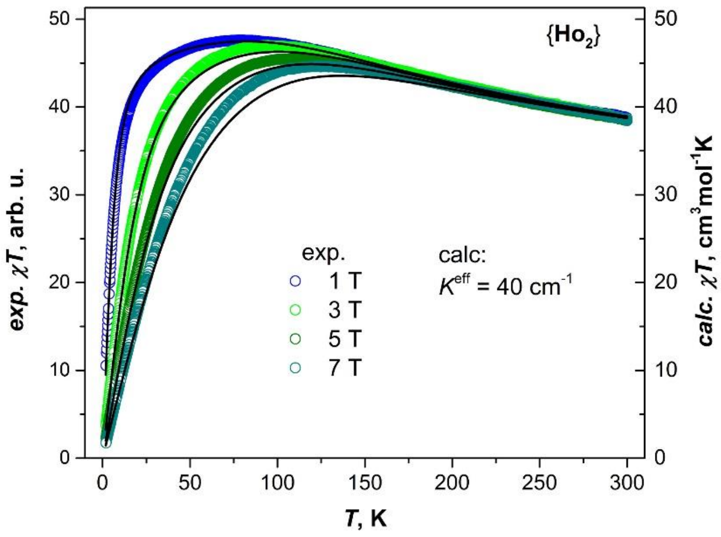

Supplementary Figure 55. Experimental χT of {Ho_2} measured in the fields of 1, 3, 5, and 7 T (dots) compared to the calculated curves with the exchange parameter K^{eff} = 40 cm^{-1} (black lines).

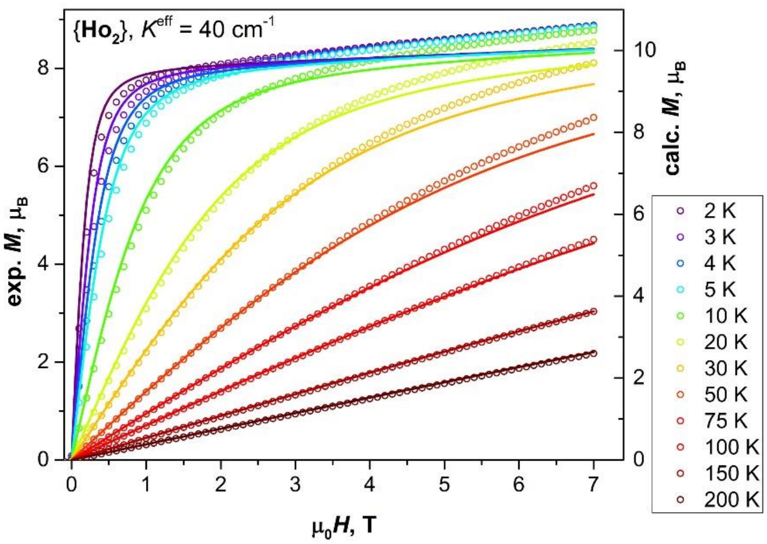

Supplementary Figure 56. Magnetization curves of {Ho_2} (dots) compared to the results of calculations for K^{eff} = 40 cm^{-1} (lines). Whereas the curve calculated for 2 K shows saturation already after 2 T, the experimental curve show continuous growth of magnetization in the whole accessible field range. The deviation of the shape of experimental and calculated curves is seen at all temperatures. We suggest that this is caused by the limited applicability of the spin Hamiltonian (Supplementary Equation 1) for {Ho_2} with two non-collinear Ho spins.

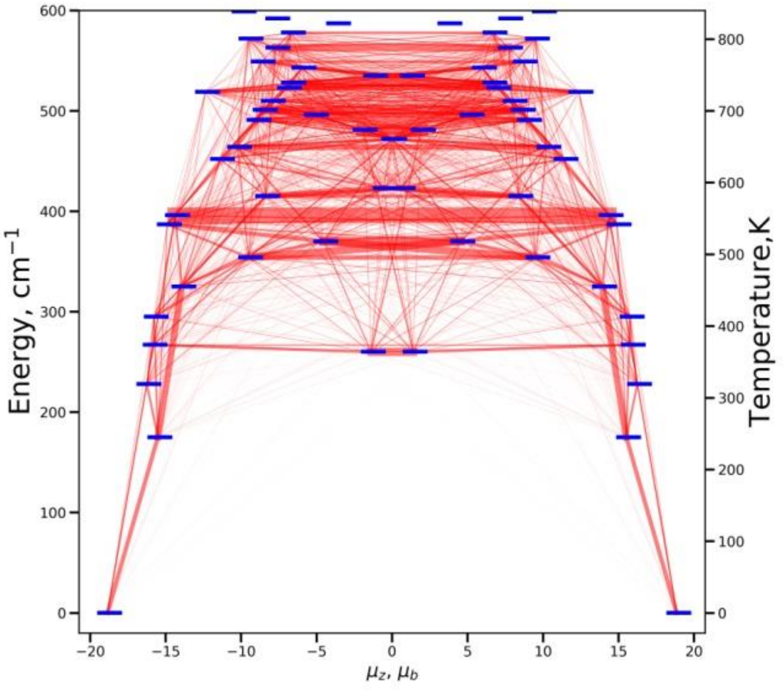

Supplementary Figure 57. The spectrum (low-energy part only) of the effective spin Hamiltonian (Supplementary Equation 1) for {Ho_2} with K^{eff} of 40 cm^{-1}. Transition probabilities for the low-energy range are visualized as lines of different thickness (thicker lines correspond to higher probabilities). The *x* axis is the projection of magnetic moment upon the main anisotropy axis.

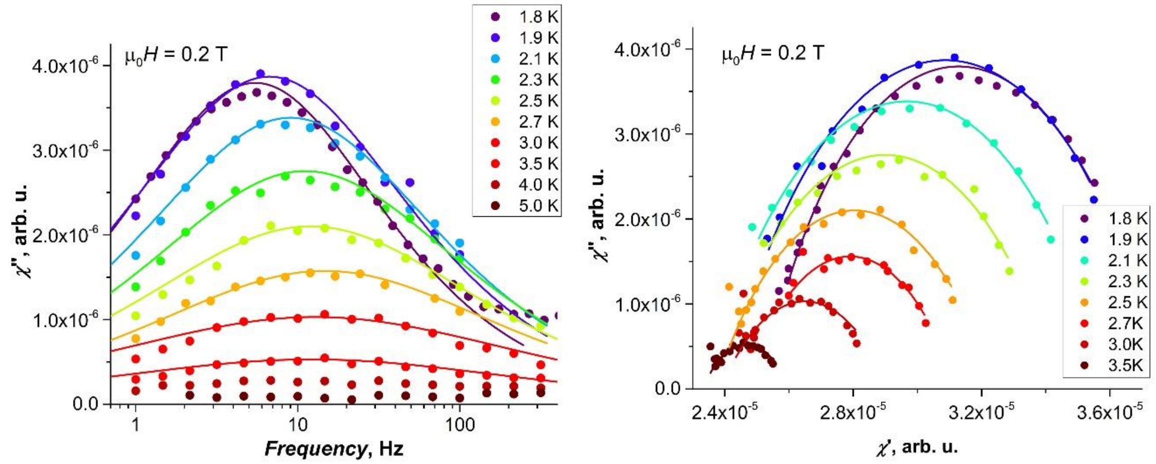

Supplementary Figure 58. AC measurements of {Er_2}. Temperature dependence of χ” in the field of 0.2 T (left) and Cole-Cole plots (right). Dots are experimental data, lines are results of the fit with generalized Debye model.

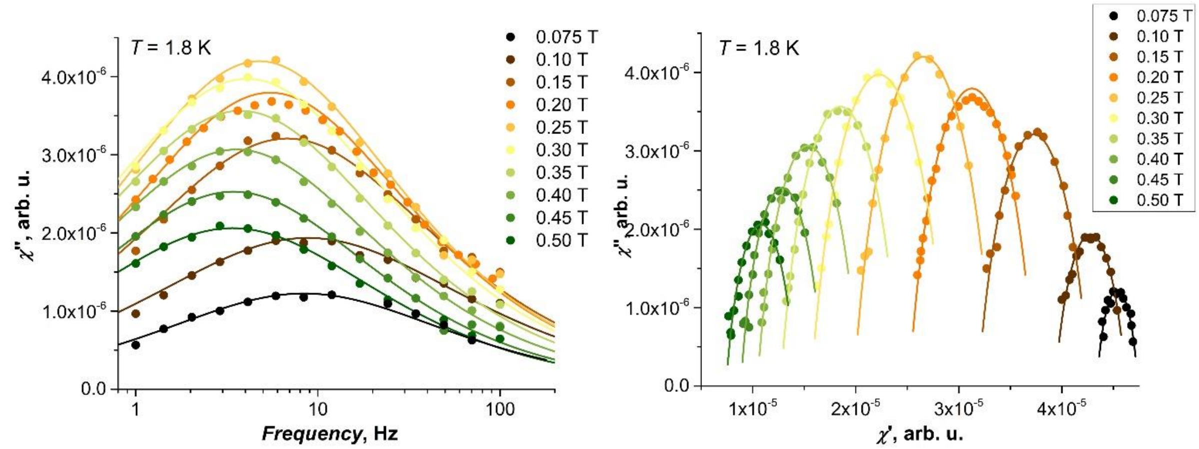

Supplementary Figure 59. AC measurements of {Er_2}. Field dependence of χ” at 1.8 K (left) and Cole-Cole plots (right).

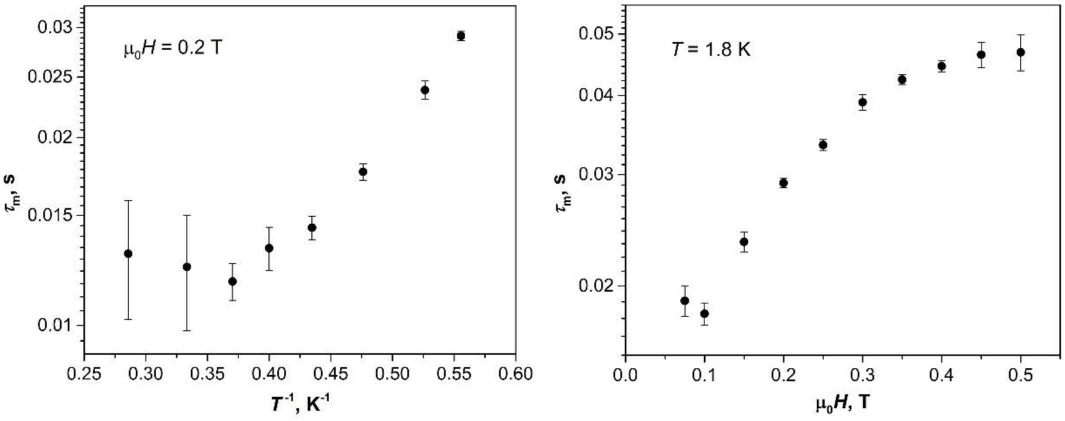

Supplementary Figure 60. Relaxation times of {Er_2}: temperature dependence in the field of 0.2 T (left) and field dependence at 1.8 K (right).

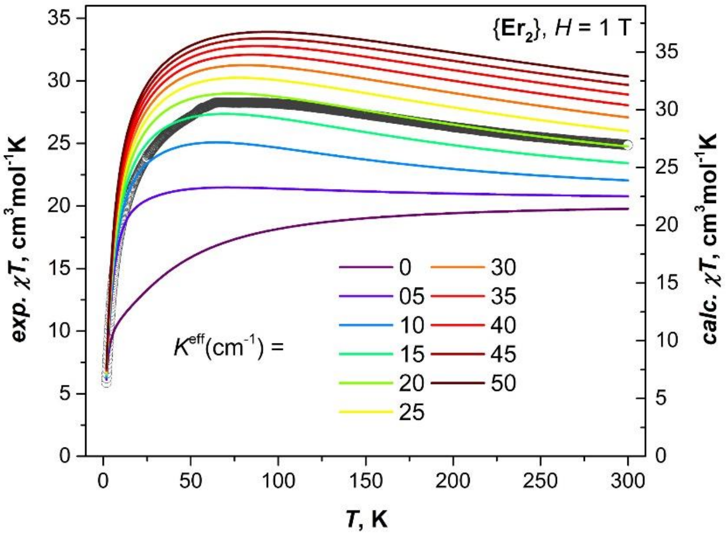

Supplementary Figure 61. Experimental χT curve of {Er_2} measured in the field of 1 T (dots) and compared to the calculated curves with different values of the exchange parameter K^{eff}. Calculated curves are close to the experimental data for K^{eff} of 20 cm^{-1}.

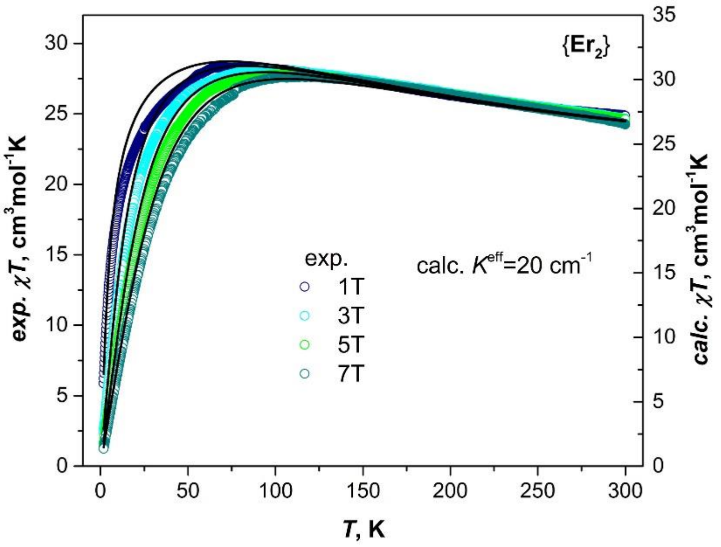

Supplementary Figure 62. Experimental χT of {Er_2} measured in the fields of 1, 3, 5, and 7 T (dots) compared to the calculated curves with the exchange parameter K^{eff} = 20 cm^{-1} (black lines).

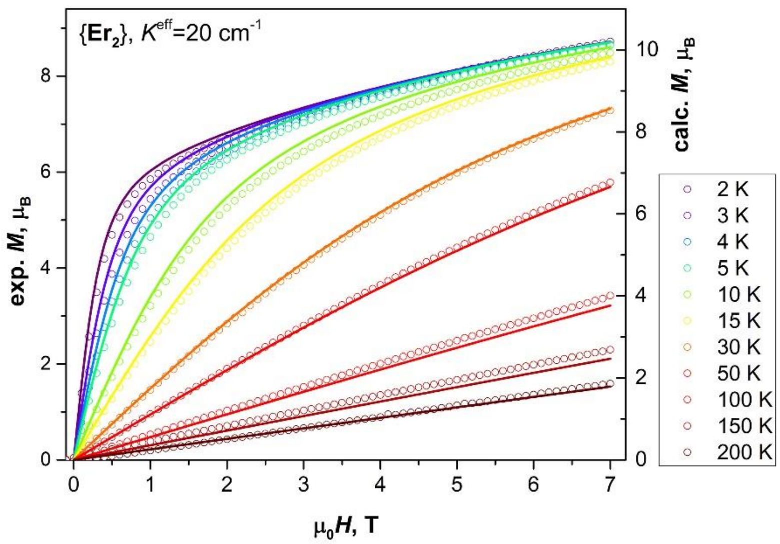

Supplementary Figure 63. Magnetization curves of $\{Er_2\}$ (dots) compared to the results of calculations for K^{eff} = 20 cm^{-1} (lines). The deviation of the shape of experimental and calculated curves is seen at all temperatures. We suggest that this is caused by the limited applicability of the spin Hamiltonian (Supplementary Equation 1) for $\{Er_2\}$ with isotropic exchange for two easy-plane Er spins.

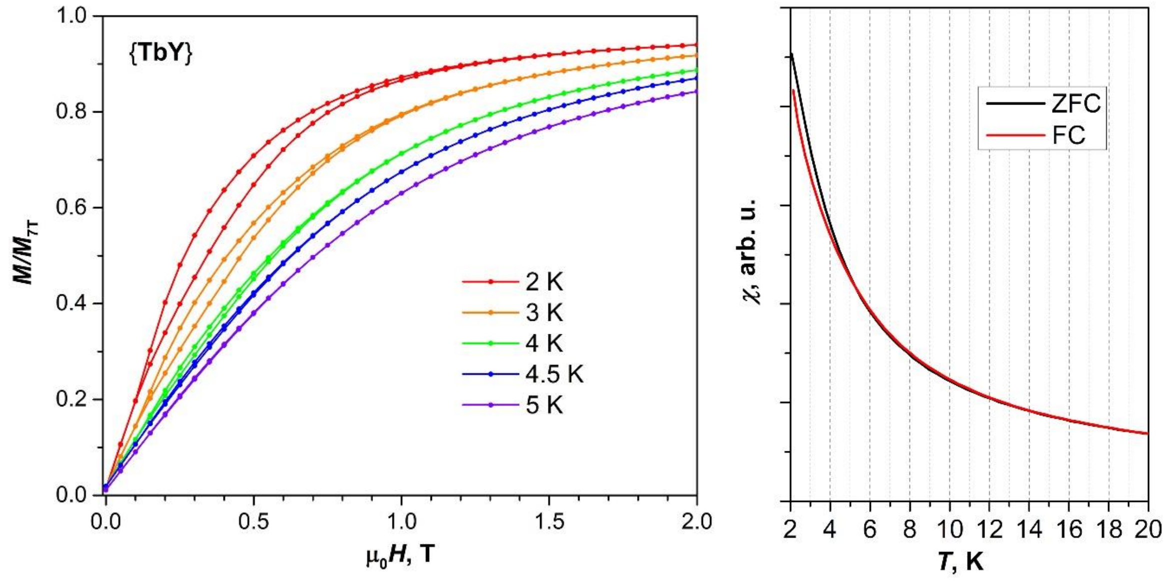

Supplementary Figure 64. Left: low-temperature magnetization curves of {TbY} showing magnetic hysteresis below 5 K. The curves are normalized by the magnetization value of the sample measured at 7 T at 2 K. Right: Comparison between χ_{FC} and χ_{ZFC} showing divergence of the two curves below 5 K.

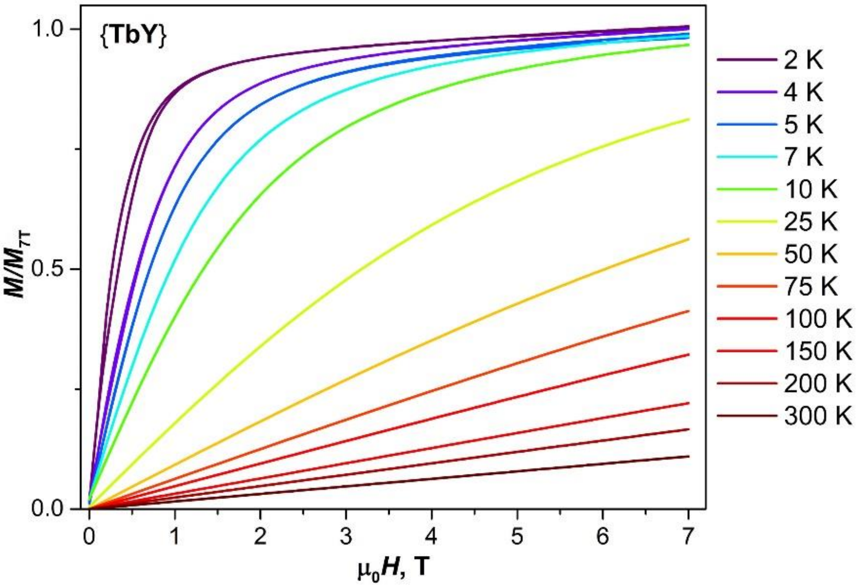

Supplementary Figure 65. Magnetization curves of {TbY} measured in the range of 2 – 300 K. The curves are normalized by the magnetization value of the sample measured at 7 T at 2 K.

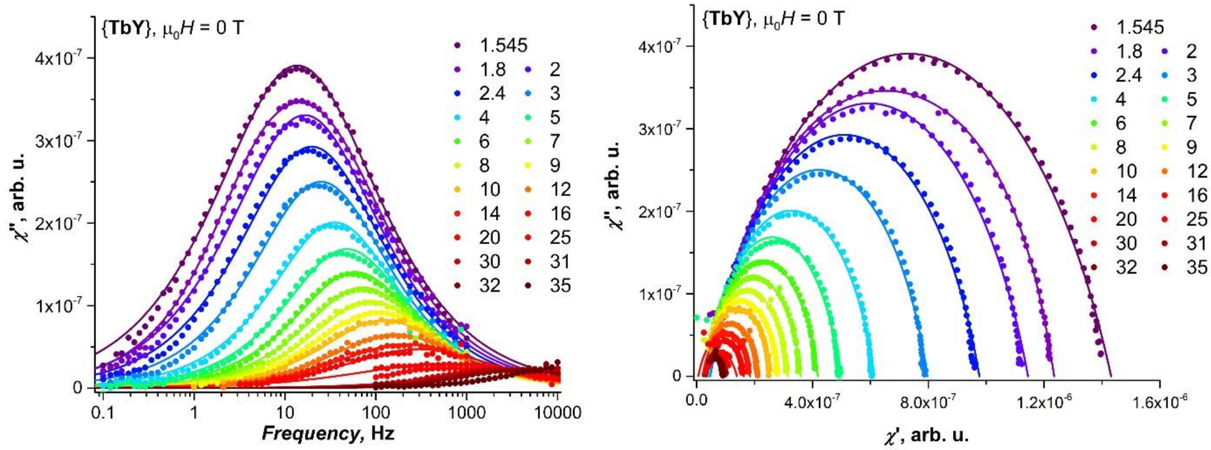

Supplementary Figure 66. Temperature dependence of χ” in zero field (left) and corresponding Cole-Cole plots (right). Dots are experimental data, lines are results of the fit with generalized Debye model.

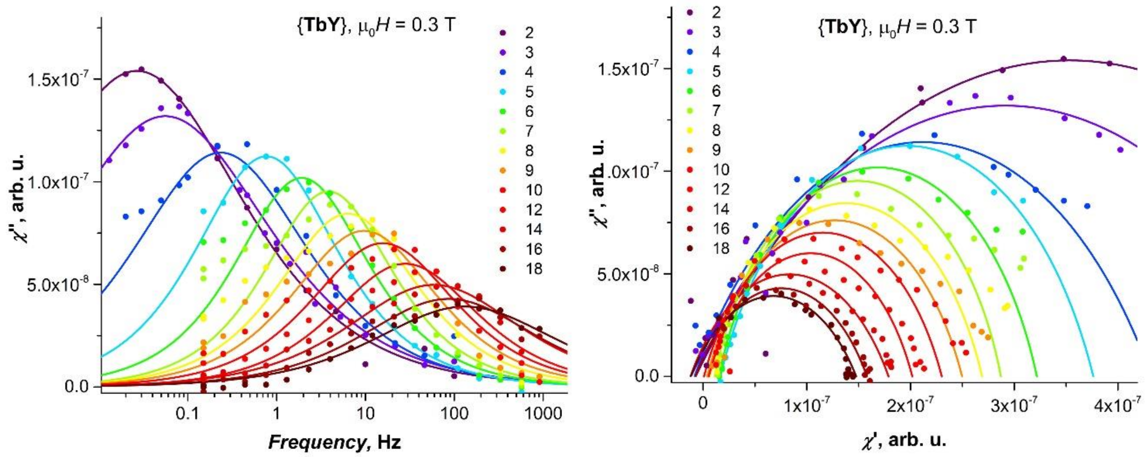

Supplementary Figure 67. Temperature dependence of χ” in the field of 0.3 T (left) and corresponding Cole-Cole plots (right). Dots are experimental data, lines are results of the fit with generalized Debye model.

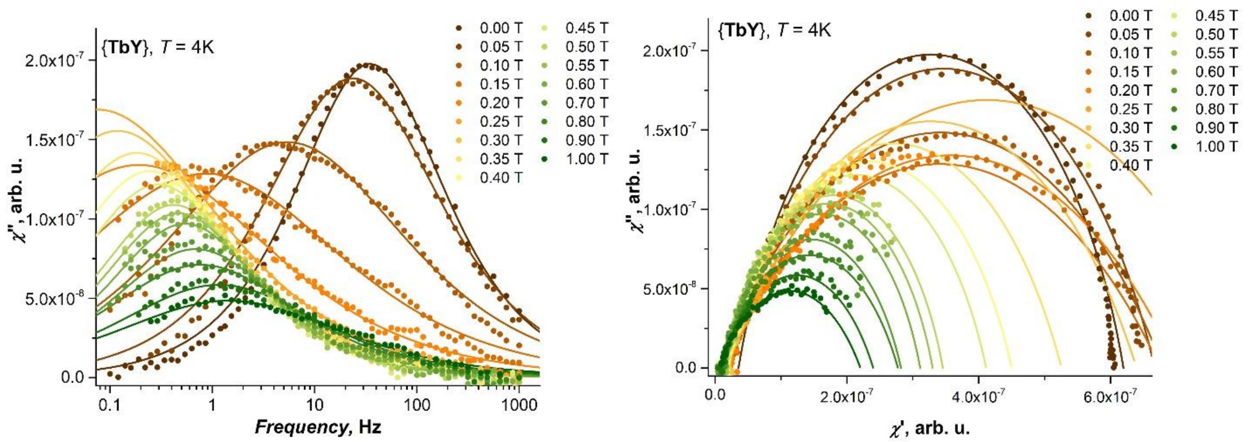

Supplementary Figure 68. Field dependence of χ” at 1.8 K (left) and Cole-Cole plots (right).

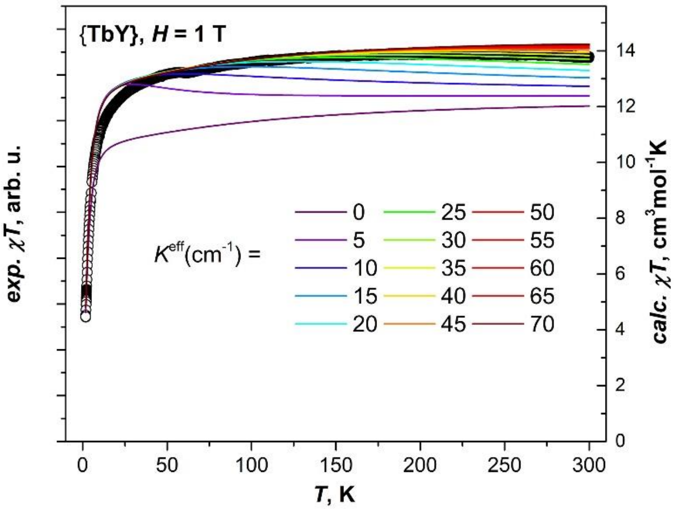

Supplementary Figure 69. Experimental χT curve of {TbY} measured in the field of 1 T (dots) and compared to the calculated curves with different values of the exchange parameter K^{eff}. Calculated curves are close to the experimental data for K^{eff} in the range from 30 to 40 cm^{-1}, however the low-temperature part is not reproduced well. Absolute experimental χT values are not known and are scaled here to reproduce the calculated data. The optimal K^{eff} value is smaller than for {Tb_2}, presumably because of the asymmetry of the unpaired electron distribution between Tb and Y.

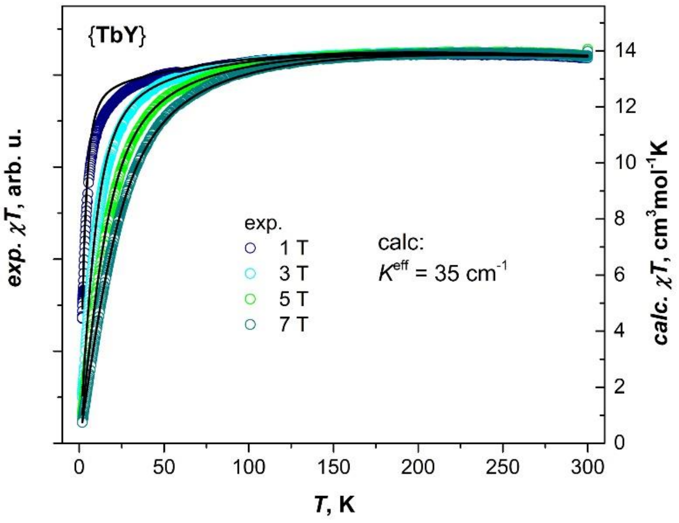

Supplementary Figure 70. Experimental χT of {TbY} measured in the fields of 1, 3, 5, and 7 T (dots) compared to the calculated curves with the exchange parameter K^{eff} = 35 cm^{-1} (black lines). Note that high-field curves are in better agreement with calculations than the curve measured at 1 T.

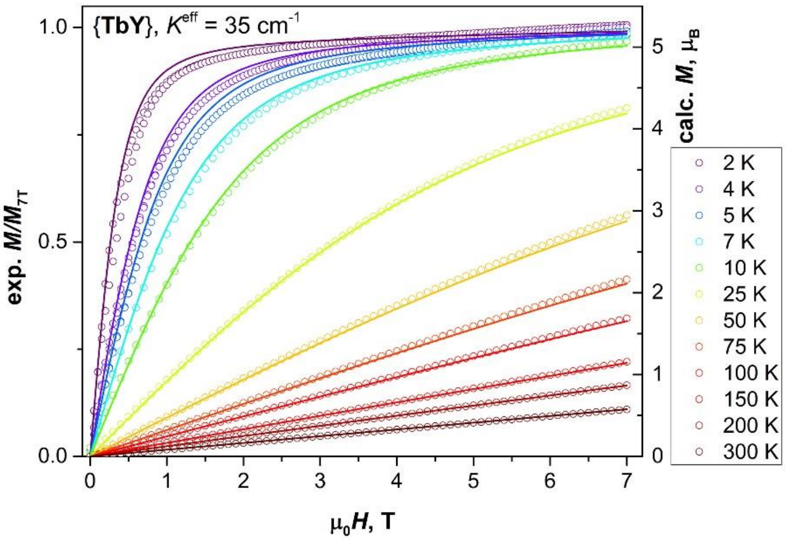

Supplementary Figure 71. Magnetization curves of {TbY} (dots) compared to the results of calculations for K^{eff} = 35 cm^{-1} (lines). Experimental points are normalized by the magnetization measured at 2 K and 7 T. Low-temperature curves show a noticeable deviation from the calculated ones. In particular, the calculated curve saturate at high fields, whereas experimental ones show continuous growth of magnetization in the whole accessible field range. We suggest that this is caused by the limited applicability of the spin Hamiltonian in Supplementary Equation 3 for {TbY} with one Tb spin and asymmetrically distributed unpaired electron. The kinetic term in the Hamiltonian (which is neglected in current simulations) may play a more important role here.

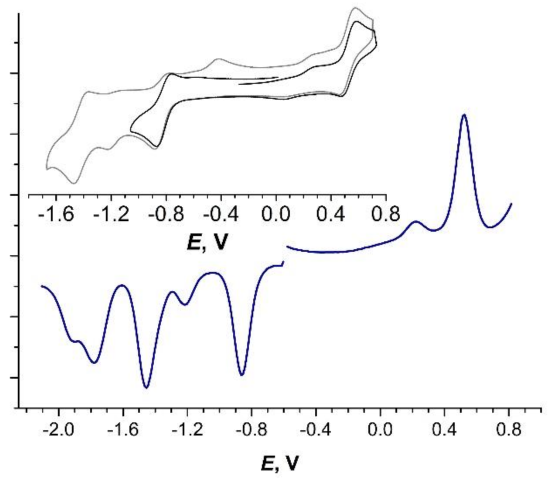

Supplementary Figure 72. Square-wave voltammetry (dark blue) and cyclic voltammetry of {Gd_2}

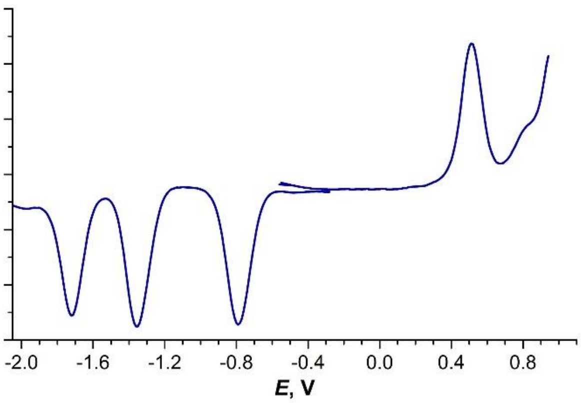

Supplementary Figure 73. Square-wave voltammetry (dark blue) of {Tb_2}

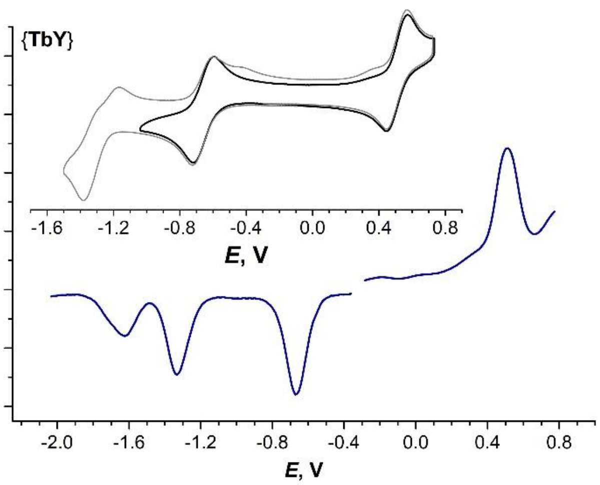

Supplementary Figure 74. Square-wave voltammetry (dark blue) and cyclic voltammetry of {TbY}

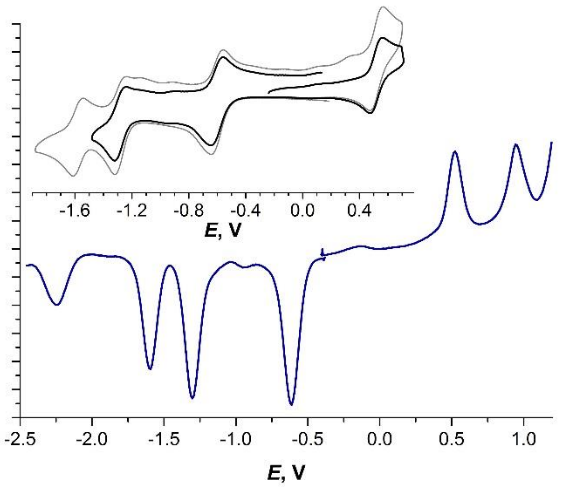

Supplementary Figure 75. Square-wave voltammetry (dark blue) and cyclic voltammetry of {Dy_2}

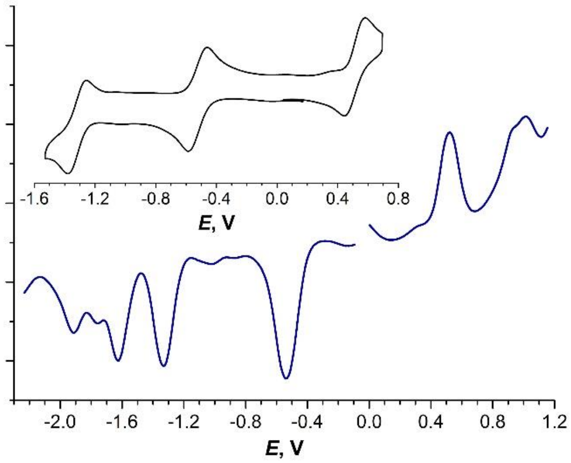

Supplementary Figure 76. Square-wave voltammetry (dark blue) and cyclic voltammetry of {Ho_2}

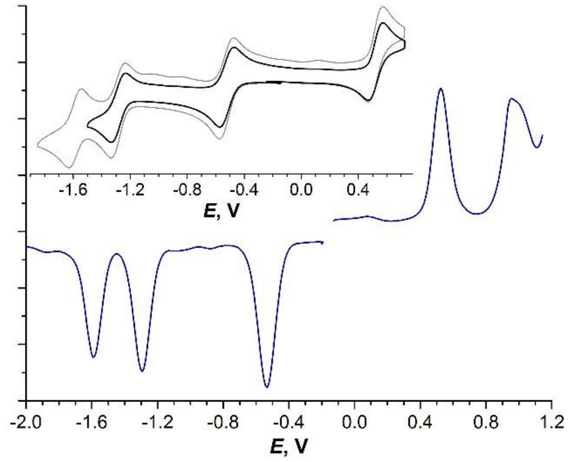

Supplementary Figure 77. Square-wave voltammetry (dark blue) and cyclic voltammetry of {Y_2}

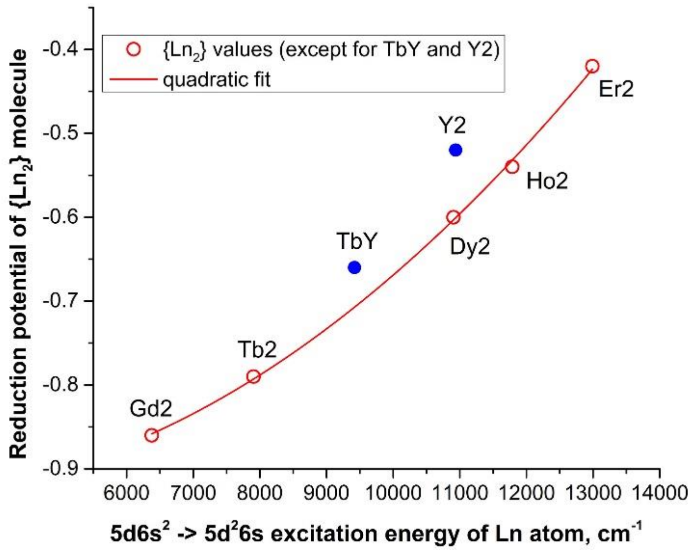

Supplementary Figure 78. Reduction potential of $\{Ln_2\}$ versus the $4f^n5d^16s^2 \rightarrow 4f^n5d^26s^1$ excitation energies of corresponding lanthanide atoms.

Supplementary Table 1. Crystal data and data collection parameters.

Crystal	$Dy_2@I_h(7)$-$C_{80}(CH_2Ph)$ $\cdot 0.67(C_7H_8)$	$Dy_2@I_h(7)$-$C_{80}(CH_2Ph)$ $\cdot 0.67(C_7H_8)$	$Dy_2@I_h(7)$-$C_{80}(CH_2Ph)$ $\cdot 0.67(C_7H_8)$	$Dy_2@I_h(7)$-$C_{80}(CH_2Ph)$ $\cdot 0.67(C_7H_8)$
Formula	$C_{91.66}$ $H_{12.33}$ Dy_2	$C_{91.66}$ $H_{12.33}$ Dy_2	$C_{91.66}$ $H_{12.33}$ Dy_2	$C_{91.66}$ $H_{12.33}$ Dy_2
Formula weight	1438.27	1438.27	1438.27	1438.27
Color, habit	Black, block	Black, block	Black, block	Black, block
Crystal system	triclinic	triclinic	triclinic	triclinic
Space group	$P\bar{1}$	$P\bar{1}$	$P\bar{1}$	$P\bar{1}$
a, Å	10.890(2)	10.910(2)	10.920(2)	10.930(2)
b, Å	11.040(2)	11.040(2)	11.050(2)	11.060(2)
c, Å	19.300(4)	19.320(4)	19.320(4)	19.320(4)
α, deg	85.91(3)	85.97(3)	86.05(3)	86.08(3)
β, deg	88.89(3)	88.94(3)	89.03(3)	89.08(3)
γ, deg	78.06(3)	78.05(3)	78.05(3)	78.05(3)
Volume, Å^3	2264.3(8)	2270.9(8)	2275.3(8)	2279.6(8)
Z	2	2	2	2
T, K	100	130	160	190
Radiation (λ, Å)	Synchrotron Radiation (0.89429)	Synchrotron Radiation (0.89429)	Synchrotron Radiation (0.89429)	Synchrotron Radiation (0.89429)
Unique data (R_{int})	9024 (0.055)	8999 (0.0574)	9002 (0.0584)	8849 (0.0626)
Parameters	857	912	917	917
Restraints	770	788	776	782
Observed data ($I > 2\sigma(I)$)	7591	7165	6877	6440
$R_1{}^a$ (observed data)	0.0792	0.0771	0.0804	0.0834
$wR_2{}^b$ (all data)	0.2570	0.2253	0.2413	0.2517
CCDC NO.	1519744	1851777	1851780	1851783

^{a}For data with $I > 2\sigma(I)$, $R_1 = \frac{\sum||F_o|-|F_c||}{\sum|F_o|}$. ^{b}For all data, $wR_2 = \sqrt{\frac{\sum[w(F_o^2-F_c^2)^2]}{\sum[w(F_o^2)^2]}}$.

Supplementary Table 1 (continued). Crystal data and data collection parameters

Crystal	$Dy_2@I_h(7)$-$C_{80}(CH_2Ph)$ $\cdot 0.67(C_7H_8)$	$Dy_2@I_h(7)$-$C_{80}(CH_2Ph)$ $\cdot 0.67(C_7H_8)$	$Dy_2@I_h(7)$-$C_{80}(CH_2Ph)$ $\cdot 0.67(C_7H_8)$	$Dy_2@I_h(7)$-$C_{80}(CH_2Ph)$ $\cdot 0.67(C_7H_8)$
Formula	$C_{91.66}$ $H_{12.33}$ Dy_2	$C_{91.66}$ $H_{12.33}$ Dy_2	$C_{91.66}$ $H_{12.33}$ Dy_2	$C_{91.66}$ $H_{12.33}$ Dy_2
Formula weight	1438.27	1438.27	1438.27	1438.27
Color, habit	Black, block	Black, block	Black, block	Black, block
Crystal system	triclinic	triclinic	triclinic	triclinic
Space group	$P\bar{1}$	$P\bar{1}$	$P\bar{1}$	$P\bar{1}$
a, Å	10.950(2)	10.960(2)	10.960(2)	10.970(2)
b, Å	11.060(2)	11.070(2)	11.080(2)	11.080(2)
c, Å	19.330(4)	19.340(4)	19.350(4)	19.350(4)
α, deg	86.14(3)	86.17(3)	86.18(3)	86.20(3)
β, deg	89.12(3)	89.16(3)	89.17(3)	89.19(3)
γ, deg	78.06(3)	78.06(3)	78.06(3)	78.06(3)
Volume, Å^3	2285.2(8)	2290.6(8)	2293.9(8)	2296.0(8)
Z	2	2	2	2
T, K	220	250	270	290
Radiation (λ, Å)	Synchrotron Radiation (0.89429)	Synchrotron Radiation (0.89429)	Synchrotron Radiation (0.89429)	Synchrotron Radiation (0.89429)
Unique data (R_{int})	8541 (0.0633)	8864 (0.0702)	8714 (0.0706)	8217 (0.0688)
Parameters	917	917	917	917
Restraints	788	782	782	782
Observed data ($I > 2\sigma(I)$)	6311	5888	5666	5296
$R_1{}^a$ (observed data)	0.0992	0.1057	0.1186	0.1300
$wR_2{}^b$ (all data)	0.2924	0.3398	0.3709	0.3990
CCDC NO.	1851781	1851779	1851778	1851782

^{a}For data with $I > 2\sigma(I)$, $R_1 = \frac{\sum||F_o|-|F_c||}{\sum|F_o|}$. ^{b}For all data, $wR_2 = \sqrt{\frac{\sum[w(F_o^2-F_c^2)^2]}{\sum[w(F_o^2)^2]}}$.

Supplementary Table 2. Metal site occupancy in {Dy_2} as a function of temperatures

Dy sites	100K	130K	160K	190K	220K	250K	270K	290K
Dy1A	0.6983(17)	0.6028(17)	0.5253(17)	0.4781(18)	0.441(2)	0.422(2)	0.409(2)	0.384(3)
Dy2A	0.657(2)	0.497(2)	0.453(2)	0.439(2)	0.388(2)	0.376(2)	0.360(2)	0.339(3)
Dy1B	0.3016(17)	0.3491(18)	0.346(2)	0.361(2)	0.372(3)	0.367(3)	0.361(3)	0.355(3)
Dy2B	0.2910(18)	0.3424(17)	0.3798(17)	0.4048(19)	0.417(2)	0.422(2)	0.425(3)	0.421(3)
Dy1C	-	0.0481(19)	0.0732(19)	0.0909(19)	0.109(2)	0.131(2)	0.149(2)	0.178(2)
Dy2C	0.052(2)	0.0416(18)	0.0645(17)	0.0806(18)	0.097(2)	0.121(2)	0.140(2)	0.167(2)
Dy1D	-	-	0.056(2)	0.076(2)	0.097(2)	0.080(2)	0.081(3)	0.083(3)
Dy2D	-	0.119(2)	0.103(2)	0.070(2)	0.078(3)	0.080(2)	0.076(2)	0.074(2)

Supplementary Table 3. Cartesian DFT-optimized molecule structure of {Tb_2}, Å

96				C	12.85833654	14.45878480	10.91310324
				C	12.19866534	14.04299956	9.71367878
C	12.32929061	9.58470405	13.65031467	C	12.34452503	15.57531650	13.05695846
C	12.34624539	10.47782857	14.79336496	C	12.30242490	15.55120913	11.62656014
C	11.13305511	10.26253475	15.55519808	C	10.50520772	15.78175814	14.72082950
C	11.11122927	8.84486495	13.69294116	C	11.13212113	16.20513564	13.52070880
C	10.37444906	9.25374887	14.86357693	C	8.34649124	14.83218372	15.55518826
C	10.41384674	8.46895968	12.49169015	C	9.08239619	15.81871589	14.79028179
C	11.03891736	8.78614382	11.25116539	C	6.47770892	13.32620510	14.87489032
C	12.91588764	9.98374194	12.40485473	C	7.09247794	14.61208793	14.86073173
C	12.26823530	9.53826866	11.20781234	C	5.74554643	11.53435745	13.29196755
C	13.59994564	11.22906513	12.36520635	C	5.70735076	11.51038496	11.85807290
C	12.93603598	11.74469805	14.74002453	C	5.76622613	12.91658262	13.71606841
C	13.88560062	12.08672579	13.60314608	C	5.70932559	12.87468407	11.39230005
C	12.35880649	12.78422633	15.50151848	C	5.73962239	13.74506986	12.53880556
C	10.53022970	11.29045275	16.34725236	C	6.96514375	12.20777668	9.38538139
C	11.17422055	12.57506590	16.30875367	C	8.10591272	12.41125199	8.50059062
C	9.07708816	11.28400181	16.47966090	C	6.36287386	13.24704003	10.18286234
C	8.95573774	9.29220697	14.87182290	C	10.22599227	13.69582443	8.49326768
C	8.34176734	10.33473098	15.66612995	C	10.98855814	14.66024572	9.26643059
C	8.99065742	8.43165094	12.53578377	C	8.77523147	13.71269990	8.51995133
C	8.26553023	8.83826168	13.71356412	C	11.06906078	16.17270764	11.19632291
C	8.20070080	8.79304836	11.38757908	C	10.34297474	16.56924337	12.37107693
C	10.24974893	9.15387206	10.10959146	C	10.38056890	15.70265857	10.04640365
C	8.83011636	9.20503775	10.18093708	C	8.29502856	16.18890482	13.65323531
C	12.23341441	10.36737805	10.04384357	C	7.06171773	15.44342098	13.69697933
C	10.97945359	10.14022719	9.34829422	C	6.41924199	14.99724894	12.49876991
C	13.52518516	12.05468061	11.23491546	C	8.91802623	16.52255442	12.40991140
C	12.82530577	11.65374612	10.05047099	C	6.96919745	14.53175512	10.11857113
C	13.63249725	13.49772053	13.06117858	C	8.18671945	14.72858632	9.35163323
C	13.54367675	13.45207250	11.66256597	C	8.95617131	15.74178932	10.04708012
C	12.35988370	14.15176092	15.04031669	C	6.99662910	15.40248521	11.25421583
C	12.96465988	14.51696247	13.78820242	C	8.22980090	16.14759736	11.21081292
C	10.42241986	13.82021055	16.32128685	C	15.36576536	11.89939508	14.14763827
C	11.16369926	14.77079822	15.50350467	C	16.45509758	12.20584845	13.15296090
C	8.30189354	12.54314159	16.50483430	C	16.99031961	13.49855853	13.05190517
C	8.97813332	13.83960984	16.38203554	C	18.00420042	13.78325050	12.13522107
C	7.13093595	10.95516151	15.21268952	C	18.49808710	12.77639039	11.30249917
C	7.11678723	12.31247890	15.68365026	C	17.97526942	11.48425440	11.39452454
C	7.03209103	9.44752014	13.29155192	C	16.96139085	11.20347473	12.31254280
C	6.99033755	9.42046183	11.85599953	Tb	9.39789063	12.46499701	14.42576983
C	6.46169086	10.53969465	14.00977306	Tb	9.39890300	12.46511450	10.48441762
C	6.38542916	10.48809364	11.13620355	H	15.46244464	12.54927669	15.03086329
C	8.18239711	10.22940179	9.39905195	H	15.44616122	10.85803962	14.49529475
C	6.99762239	10.86512123	9.89178937	H	16.61064920	14.29005962	13.70320029
C	10.33826911	11.15182295	8.56401922	H	18.41163147	14.79440278	12.07375513
C	8.88214823	11.15351801	8.52610151	H	19.29234654	12.99702786	10.58644342
C	12.19167993	12.67532180	9.27686736	H	16.55944735	10.18921579	12.38257954
C	10.98751858	12.44282491	8.51165395	H	18.36027474	10.69023778	10.75170847

Supplementary Table 4. Cartesian DFT-optimized molecule structure of $\{Dy_2\}$, Å

96			
C	12.32934302	9.58464014	13.65014868
C	12.34659639	10.47779193	14.79316747
C	11.13316671	10.26237178	15.55465269
C	11.11138787	8.84462724	13.69279692
C	10.37463701	9.25333984	14.86332672
C	10.41383971	8.46892556	12.49167168
C	11.03902522	8.78608272	11.25131181
C	12.91608456	9.98362540	12.40479977
C	12.26832239	9.53813848	11.20789326
C	13.60006977	11.22895464	12.36527939
C	12.93606490	11.74452227	14.73987488
C	13.88588950	12.08667275	13.60308998
C	12.35905758	12.78435791	15.50088056
C	10.53005809	11.29074433	16.34516005
C	11.17372747	12.57515760	16.30658359
C	9.07726541	11.28431345	16.47831014
C	8.95604930	9.29201521	14.87131687
C	8.34231477	10.33540727	15.66482718
C	8.99051420	8.43136935	12.53578427
C	8.26543333	8.83806977	13.71342684
C	8.20058873	8.79280911	11.38767087
C	10.24987777	9.15357347	10.10978794
C	8.83027981	9.20475559	10.18127438
C	12.23372109	10.36716886	10.04393650
C	10.97963797	10.14000287	9.34880941
C	13.52535487	12.05472615	11.23499784
C	12.82550332	11.65351113	10.05063072
C	13.63254373	13.49770752	13.06109284
C	13.54374548	13.45200315	11.66268760
C	12.36036291	14.15211805	15.04025463
C	12.96505363	14.51720410	13.78824094
C	10.42233730	13.81982752	16.31914752
C	11.16398026	14.77083861	15.50296236
C	8.30219750	12.54317341	16.50371519
C	8.97825872	13.83933198	16.38015238
C	7.13149779	10.95551961	15.21155009
C	7.11773516	12.31268586	15.68229039
C	7.03206807	9.44733354	13.29144078
C	6.99027578	9.42027905	11.85609955
C	6.46136189	10.53945615	14.00957544
C	6.38502454	10.48779547	11.13635300
C	8.18277071	10.22987285	9.40010594
C	6.99784322	10.86523829	9.89257276
C	10.33814325	11.15214861	8.56615971
C	8.88230461	11.15385402	8.52763673
C	12.19184435	12.67544033	9.27755853
C	10.98715879	12.44293397	8.51388015
C	12.85879137	14.45905610	10.91311450
C	12.19916906	14.04325301	9.71382487
C	12.34440828	15.57524847	13.05682287
C	12.30243184	15.55116166	11.62669981
C	10.50521932	15.78195848	14.72055300
C	11.13215823	16.20517481	13.52058457
C	8.34652171	14.83207844	15.55424850
C	9.08231857	15.81886092	14.78998119
C	6.47775714	13.32652548	14.87428353
C	7.09217124	14.61254276	14.86046390
C	5.74558063	11.53444480	13.29188839
C	5.70733992	11.51046577	11.85816099
C	5.76606962	12.91664846	13.71594483
C	5.70910155	12.87472530	11.39242236
C	5.73932904	13.74506191	12.53882971
C	6.96594350	12.20786559	9.38659705
C	8.10626440	12.41121942	8.50189169
C	6.36290018	13.24725839	10.18342094
C	10.22594188	13.69540074	8.49547745
C	10.98870892	14.66016351	9.26718283
C	8.77544298	13.71245915	8.52175699
C	11.06916053	16.17283256	11.19645393
C	10.34297031	16.56927638	12.37107433
C	10.38056908	15.70284165	10.04673862
C	8.29492384	16.18916287	13.65311840
C	7.06141638	15.44378836	13.69687188
C	6.41911437	14.99743021	12.49875486
C	8.91793821	16.52273541	12.40990277
C	6.96902297	14.53202048	10.11891253
C	8.18686606	14.72836776	9.35267228
C	8.95617154	15.74191909	10.04739225
C	6.99640525	15.40275159	11.25435010
C	8.22967970	16.14785282	11.21089096
C	15.36576103	11.89938056	14.14763270
C	16.45514792	12.20581847	13.15299543
C	16.99029465	13.49846442	13.05195526
C	18.00426564	13.78326514	12.13513705
C	18.49811289	12.77645943	11.30246181
C	17.97529923	11.48425931	11.39446036
C	16.96130358	11.20355578	12.31263868
Dy	9.39464248	12.46376818	14.43669057
Dy	9.39609012	12.46415098	10.47358549
H	15.46236101	12.54922390	15.03094601
H	15.44608571	10.85804563	14.49539868
H	16.61061217	14.29008832	13.70322357
H	18.41162411	14.79440564	12.07369516
H	19.29227823	12.99703536	10.58643268
H	16.55933466	10.18922168	12.38252755
H	18.36028728	10.69024765	10.75170511

Supplementary Table 5. Cartesian DFT-optimized molecule structure of $\{Ho_2\}$, Å

96				C	12.85885880	14.45911558	10.91324489
				C	12.19943264	14.04342211	9.71407352
C	12.32935897	9.58481932	13.65001529	C	12.34447154	15.57520875	13.05688187
C	12.34640970	10.47780227	14.79288752	C	12.30243047	15.55120621	11.62669412
C	11.13318774	10.26246182	15.55377409	C	10.50542778	15.78187768	14.72037727
C	11.11139951	8.84443953	13.69271806	C	11.13221748	16.20537251	13.52050623
C	10.37476687	9.25318529	14.86296443	C	8.34688974	14.83151729	15.55301907
C	10.41388579	8.46866972	12.49174172	C	9.08256020	15.81864917	14.78944048
C	11.03911460	8.78583761	11.25144837	C	6.47817164	13.32653664	14.87368308
C	12.91606289	9.98361635	12.40487094	C	7.09231557	14.61243250	14.85981192
C	12.26839670	9.53815272	11.20807367	C	5.74561002	11.53445522	13.29187048
C	13.60017581	11.22903896	12.36529327	C	5.70730093	11.51048107	11.85802181
C	12.93636963	11.74476050	14.73971517	C	5.76595178	12.91667096	13.71573187
C	13.88573056	12.08667111	13.60305979	C	5.70889795	12.87479650	11.39254311
C	12.35940949	12.78448656	15.50037676	C	5.73922671	13.74521179	12.53876663
C	10.52963571	11.29118647	16.34323368	C	6.96670490	12.20821705	9.38779216
C	11.17346354	12.57511876	16.30454575	C	8.10626727	12.41129416	8.50216430
C	9.07731182	11.28444175	16.47779386	C	6.36321897	13.24737834	10.18393110
C	8.95620874	9.29201971	14.87092370	C	10.22569687	13.69514653	8.49714424
C	8.34289740	10.33577642	15.66364711	C	10.98868337	14.66010155	9.26814015
C	8.99061627	8.43119487	12.53579336	C	8.77560575	13.71217987	8.52303716
C	8.26557806	8.83793883	13.71333297	C	11.06922360	16.17304196	11.19657896
C	8.20076806	8.79281276	11.38781585	C	10.34305028	16.56953217	12.37110499
C	10.25002903	9.15335995	10.11014430	C	10.38068197	15.70276188	10.04709889
C	8.83054327	9.20491859	10.18170383	C	8.29504132	16.18918965	13.65295767
C	12.23361590	10.36719982	10.04435763	C	7.06153151	15.44383298	13.69666646
C	10.97961202	10.14011547	9.34973032	C	6.41905620	14.99736217	12.49874108
C	13.52523853	12.05459795	11.23521276	C	8.91802758	16.52289634	12.40989936
C	12.82544919	11.65366717	10.05090573	C	6.96901499	14.53209261	10.11945602
C	13.63241544	13.49762272	13.06121459	C	8.18716987	14.72798020	9.35381546
C	13.54342211	13.45202505	11.66274283	C	8.95628910	15.74179143	10.04800264
C	12.36094208	14.15236124	15.04026112	C	6.99637267	15.40294032	11.25446873
C	12.96514569	14.51732988	13.78823709	C	8.22971223	16.14802093	11.21107274
C	10.42213946	13.81946676	16.31719749	C	15.36577831	11.89943567	14.14745361
C	11.16417438	14.77099892	15.50229715	C	16.45501044	12.20584315	13.15297574
C	8.30221126	12.54313250	16.50363206	C	16.99027311	13.49850141	13.05185715
C	8.97852116	13.83881438	16.37859970	C	18.00419968	13.78322263	12.13522545
C	7.13227191	10.95597587	15.21042304	C	18.49815097	12.77638737	11.30247334
C	7.11842887	12.31295282	15.68115605	C	17.97525377	11.48431429	11.39458829
C	7.03220676	9.44745563	13.29146208	C	16.96137233	11.20346213	12.31256511
C	6.99047498	9.42045044	11.85602061	Ho	9.38954538	12.46205487	14.44688888
C	6.46137849	10.53943208	14.00907765	Ho	9.39287952	12.46295020	10.46299080
C	6.38498986	10.48780765	11.13671931	H	15.46246465	12.54933210	15.03072772
C	8.18337307	10.23033465	9.40128514	H	15.44621556	10.85807002	14.49515275
C	6.99856887	10.86571143	9.89358188	H	16.61063586	14.29007081	13.70319316
C	10.33790670	11.15260325	8.56792825	H	18.41161600	14.79436435	12.07381814
C	8.88239586	11.15408824	8.52831224	H	19.29233919	12.99699366	10.58651823
C	12.19196321	12.67546342	9.27827123	H	16.55953486	10.18918296	12.38262739
C	10.98682191	12.44294787	8.51565006	H	18.36025765	10.69021489	10.75171219

Supplementary Table 6. Cartesian DFT-optimized molecule structure of {Er_2}, Å

96				C	12.19986615	14.04368996	9.71442089
C	12.32945447	9.58482630	13.64986302	C	12.34464418	15.57535296	13.05682705
C	12.34634674	10.47798358	14.79248747	C	12.30260502	15.55136994	11.62684784
C	11.13312218	10.26253581	15.55281939	C	10.50573484	15.78193138	14.72005006
C	11.11161044	8.84400602	13.69256705	C	11.13243324	16.20568573	13.52041266
C	10.37498928	9.25284479	14.86256878	C	8.34725624	14.83090646	15.55174703
C	10.41401204	8.46825011	12.49181011	C	9.08285405	15.81831250	14.78889498
C	11.03935640	8.78542538	11.25168623	C	6.47846627	13.32664478	14.87308236
C	12.91631914	9.98355463	12.40493318	C	7.09235919	14.61251800	14.85917938
C	12.26857593	9.53802412	11.20832792	C	5.74552614	11.53447799	13.29172057
C	13.60032953	11.22899757	12.36532563	C	5.70711757	11.51052964	11.85803616
C	12.93657255	11.74486668	14.73946202	C	5.76590026	12.91672270	13.71559182
C	13.88586688	12.08668440	13.60297874	C	5.70867167	12.87489057	11.39260389
C	12.35984595	12.78466868	15.49958707	C	5.73902962	13.74533622	12.53875282
C	10.52921461	11.29168670	16.34096712	C	6.96757878	12.20866529	9.38885844
C	11.17293597	12.57507241	16.30179284	C	8.10613718	12.41133683	8.50208348
C	9.07735117	11.28454159	16.47741044	C	6.36344597	13.24764403	10.18450822
C	8.95653361	9.29197538	14.87048868	C	10.22545756	13.69484340	8.49933112
C	8.34355183	10.33627374	15.66246166	C	10.98874706	14.66010595	9.26918412
C	8.99072408	8.43088039	12.53583344	C	8.77576360	13.71197548	8.52437034
C	8.26568576	8.83779691	13.71327228	C	11.06943972	16.17336951	11.19677657
C	8.20090381	8.79276784	11.38793335	C	10.34321416	16.56988194	12.37116223
C	10.25028688	9.15303602	10.11060081	C	10.38084777	15.70279966	10.04763290
C	8.83091848	9.20497754	10.18218440	C	8.29515181	16.18935129	13.65270566
C	12.23359003	10.36724893	10.04484914	C	7.06145136	15.44408208	13.69637416
C	10.97954536	10.14019662	9.35067701	C	6.41886343	14.99754864	12.49872833
C	13.52526861	12.05458297	11.23547950	C	8.91813072	16.52325126	12.40992752
C	12.82544782	11.65371704	10.05131475	C	6.96897522	14.53233436	10.12005035
C	13.63242551	13.49773055	13.06122286	C	8.18751823	14.72751565	9.35509129
C	13.54330522	13.45209691	11.66297348	C	8.95648086	15.74160007	10.04868627
C	12.36169376	14.15282942	15.04026516	C	6.99617293	15.40334729	11.25473691
C	12.96551082	14.51762481	13.78835848	C	8.22970819	16.14834263	11.21137194
C	10.42190096	13.81895088	16.31460044	C	15.36588300	11.89944732	14.14734627
C	11.16455127	14.77119445	15.50158629	C	16.45499716	12.20590103	13.15278990
C	8.30211364	12.54307915	16.50382848	C	16.99021052	13.49855041	13.05174165
C	8.97878599	13.83822094	16.37684478	C	18.00424229	13.78325760	12.13524907
C	7.13319432	10.95652713	15.20910154	C	18.49825412	12.77634327	11.30245685
C	7.11908755	12.31326234	15.68028572	C	17.97532607	11.48431673	11.39457117
C	7.03230166	9.44741316	13.29139206	C	16.96144295	11.20336417	12.31251083
C	6.99059538	9.42045062	11.85605057	Er	9.38226424	12.45932065	14.45501611
C	6.46147739	10.53946294	14.00856689	Er	9.38767611	12.46082536	10.45420467
C	6.38503738	10.48785115	11.13709421	H	15.46264902	12.54949245	15.03048614
C	8.18411728	10.23096087	9.40247439	H	15.44640850	10.85807350	14.49485327
C	6.99949553	10.86631811	9.89484572	H	16.61058881	14.29007355	13.70311725
C	10.33768802	11.15305457	8.56989136	H	18.41175304	14.79431036	12.07402457
C	8.88241364	11.15425684	8.52867026	H	19.29254088	12.99694141	10.58669616
C	12.19213095	12.67556067	9.27920082	H	16.55987879	10.18907072	12.38284962
C	10.98639307	12.44296821	8.51796124	H	18.36033426	10.69017902	10.75175113
C	12.85904918	14.45931211	10.91334715				

Supplementary Table 7, CAS compositions

System<br>$\{LnY\}^{-}$	CAS problems composition<br>X(Y)*	Number of states in RASSI-SO	ANISO[9]<br>KD-mapping
**Tb**	CAS(8,7)<br>7(**7**), 140(**5**), 500(**3**), 490(**1**)	250	6 KD
**Ho**	CAS(10,7)<br>35(**5**)	35	8 KD
**Er**	CAS(11,7)<br>35(**4**), 112(**2**)	147	8 KD

***X(Y), X-number roots for the system with the spin Y**

Supplementary Table 8. *CASSCF/SO-RASSI calculations summary for the {**TbY**}⁻ molecule. Energies and state compositions of pseudo Kramers doublets and singlet states of the Tb1 and Tb2 ions in single-ion frame.*

pKD	Tb1		Tb2	
	E, cm^{-1}	Composition, %	E, cm^{-1}	Composition, %
1	0.0<br>0.0	$100\|\pm6\rangle$<br>$100\|\pm6\rangle$	0.00<br>0.02	$100\|\pm6\rangle$<br>$100\|\pm6\rangle$
2	266.7<br>266.9	$100\|\pm5\rangle$<br>$100\|\pm5\rangle$	256.1<br>256.3	$100\|\pm5\rangle$<br>$100\|\pm5\rangle$
3	511.0<br>511.7	$99\|\pm4\rangle$<br>$99\|\pm4\rangle$	507.8<br>509.2	$99\|\pm4\rangle$<br>$100\|\pm4\rangle$
4	708.8<br>715.4	$99\|\pm3\rangle$<br>$99\|\pm3\rangle$	705.2<br>719.8	$99\|\pm3\rangle$<br>$98\|\pm3\rangle$
5	847.6<br>869.1	$91\|\pm2\rangle + 8\|0\rangle$<br>$98\|\pm2\rangle$	839.3<br>865.1	$87\|\pm2\rangle + 13\|0\rangle$<br>$98\|\pm2\rangle$
6	928.6<br>990.1	$97\|\pm1\rangle$<br>$94\|\pm1\rangle + 4\|0\rangle$	911.1<br>990.5	$96\|\pm1\rangle$<br>$91\|\pm1\rangle + 6\|0\rangle$
S	1001.3	$87\|0\rangle + 8\|\pm2\rangle + 5\|\pm1\rangle$	996.0	$80\|0\rangle + 12\|\pm2\rangle + 8\|\pm1\rangle$

The contribution for the "$|\pm X\rangle$" state means a sum of contributions for $|+X\rangle$ and $|-X\rangle$ states

Supplementary Table 9. *CASSCF/SO-RASSI calculations summary for the {**HoY**}⁻ molecule. Energies and state compositions of pseudo Kramers doublets and singlet states of the Ho1 and Ho2 ions in single-ion frame.*

pKD	Ho1		Ho2	
	E, cm^{-1}	Composition, %	E, cm^{-1}	Composition, %
1	0.0<br>0.1	$64\|\pm8\rangle + 14\|\pm7\rangle + 10\|\pm6\rangle$<br>$64\|\pm8\rangle + 14\|\pm7\rangle + 10\|\pm6\rangle$	0.0<br>0.6	$63\|\pm8\rangle + 19\|\pm6\rangle + 9\|\pm4\rangle$<br>$64\|\pm8\rangle + 19\|\pm6\rangle + 8\|\pm4\rangle$
2	165.6<br>172.6	$31\|\pm3\rangle + 20\|\pm2\rangle + 18\|\pm5\rangle$<br>$34\|\pm3\rangle + 13\|\pm2\rangle + 18\|\pm5\rangle$	130.1<br>138.1	$29\|\pm5\rangle + 29\|\pm3\rangle + 18\|\pm4\rangle$<br>$27\|\pm5\rangle + 23\|\pm4\rangle + 22\|\pm3\rangle$
3	262.7<br>268.0	$40\|\pm7\rangle + 28\|\pm6\rangle + 20\|\pm4\rangle$<br>$42\|\pm7\rangle + 32\|\pm6\rangle + 20\|\pm4\rangle$	245.2<br>246.9	$63\|\pm7\rangle + 19\|\pm6\rangle + 6\|\pm3\rangle$<br>$65\|\pm7\rangle + 16\|\pm6\rangle + 5\|\pm3\rangle$
4	305.6<br>342.0	$40\|\pm5\rangle + 22\|\pm2\rangle + 20\|\pm1\rangle$<br>$58\|\pm5\rangle + 12\|\pm2\rangle + 12\|\pm4\rangle$	292.9<br>323.6	$33\|\pm2\rangle + 29\|\pm5\rangle + 19\|\pm4\rangle$<br>$36\|\pm2\rangle + 25\|\pm4\rangle + 22\|\pm5\rangle$
5	378.8<br>399.8	$27\|\pm7\rangle + 18\|\pm8\rangle + 14\|\pm5\rangle$<br>$28\|\pm7\rangle + 20\|\pm8\rangle + 17\|\pm6\rangle$	363.0<br>377.1	$38\|\pm4\rangle + 19\|\pm7\rangle + 17\|\pm3\rangle$<br>$55\|\pm1\rangle + 15\|\pm3\rangle + 12\|\pm4\rangle$
	407.4<br>448.6	$40\|\pm1\rangle + 20\|0\rangle + 11\|\pm6\rangle$<br>$32\|\pm6\rangle + 31\|\pm4\rangle + 16\|\pm1\rangle$	405.3<br>428.2	$25\|\pm6\rangle + 24\|\pm7\rangle + 20\|\pm1\rangle$<br>$25\|0\rangle + 19\|\pm6\rangle + 14\|\pm1\rangle$
	450.8<br>486.6	$29\|\pm4\rangle + 24\|0\rangle + 20\|\pm3\rangle$<br>$42\|\pm3\rangle + 17\|\pm4\rangle + 15\|0\rangle$	436.0<br>451.0	$30\|\pm6\rangle + 20\|\pm5\rangle + 14\|\pm3\rangle$<br>$45\|\pm3\rangle + 22\|\pm5\rangle + 14\|\pm1\rangle$
	555.3<br>581.2	$39\|\pm3\rangle + 23\|\pm1\rangle + 16\|\pm2\rangle$<br>$29\|\pm2\rangle + 29\|0\rangle + 17\|\pm1\rangle$	526.5<br>534.2	$42\|\pm2\rangle + 17\|0\rangle + 13\|\pm4\rangle$<br>$36\|0\rangle + 22\|\pm2\rangle + 13\|\pm1\rangle$
	603.4	$47\|\pm2\rangle + 44\|\pm1\rangle + 4\|\pm6\rangle$	561.9	$61\|\pm1\rangle + 10\|\pm3\rangle + 9\|\pm2\rangle$

The contribution for the "$|\pm X\rangle$" state means a sum of contributions for $|+X\rangle$ and $|-X\rangle$ states

Supplementary Table 10. *CASSCF/SO-RASSI calculations summary for the {**ErY**}⁻ molecule. Energies and state compositions of Kramers doublets of the Er1 and Er2 ions in single-ion frame.*

KD	Er1		Er2	
	E, cm^{-1}	Composition, %	E, cm^{-1}	Composition, %
1	0.0	$61\|\pm0.5\rangle + 19\|\pm1.5\rangle$	0.0	$38\|\pm0.5\rangle + 27\|\pm1.5\rangle + 16\|\pm2.5\rangle + 10\|\pm4.5\rangle$
2	96.4	$52\|\pm1.5\rangle + 15\|\pm2.5\rangle + 10\|\pm4.5\rangle$	85.4	$30\|\pm1.5\rangle + 26\|\pm0.5\rangle + 17\|\pm2.5\rangle + 16\|\pm3.5\rangle$
3	159.3	$26\|\pm5.5\rangle + 21\|\pm4.5\rangle + 21\|\pm2.5\rangle + 10\|\pm6.5\rangle$	123.9	$31\|\pm4.5\rangle + 24\|\pm5.5\rangle + 16\|\pm3.5\rangle + 11\|\pm1.5\rangle$
4	226.6	$19\|\pm2.5\rangle + 16\|\pm3.5\rangle + 16\|\pm6.5\rangle + 14\|\pm5.5\rangle + 13\|\pm4.5\rangle + 12\|\pm0.5\rangle$	193.2	$36\|\pm2.5\rangle + 25\|\pm3.5\rangle + 11\|\pm0.5\rangle$
5	255.7	$32\|\pm3.5\rangle + 26\|\pm2.5\rangle + 11\|\pm5.5\rangle$	229.9	$22\|\pm2.5\rangle + 19\|\pm3.5\rangle + 18\|\pm0.5\rangle + 15\|\pm1.5\rangle + 12\|\pm4.5\rangle$
6	342.0	$50\|\pm6.5\rangle + 19\|\pm7.5\rangle + 13\|\pm5.5\rangle$	315.6	$39\|\pm6.5\rangle + 30\|\pm7.5\rangle + 10\|\pm5.5\rangle$
7	429.1	$33\|\pm4.5\rangle + 28\|\pm3.5\rangle + 14\|\pm5.5\rangle$	420.8	$25\|\pm4.5\rangle + 21\|\pm5.5\rangle + 18\|\pm6.5\rangle + 18\|\pm7.5\rangle + 15\|\pm3.5\rangle$
8	607.4	$67\|\pm7.5\rangle + 15\|\pm5.5\rangle + 10\|\pm4.5\rangle$	565.1	$49\|\pm7.5\rangle + 21\|\pm6.5\rangle + 19\|\pm5.5\rangle$

Supplementary Table 11. *Ab-intio derived (SINGLE_ANISO) crystal field parameters in Stevens Notation B(q,k)(cm^{-1}) for Ln-ions in the* {LnY}$^{-}$ *molecules.**

index		{TbY}$^{-}$, Tb1	{YTb^{-}}, Tb2	{HoY}$^{-}$,Ho1	{YHo}$^{-}$,Ho2	{ErY}$^{-}$,Er1	{YEr}$^{-}$,Er2
k	q	B(q,k)	B(q,k)	B(q,k)	B(q,k)	B(q,k)	B(q,k)
2	-2	-1.09E+00	2.15E-01	-2.33E-01	-1.16E-01	-4.84E-02	-3.03E-02
2	-1	4.00E-01	9.18E-02	-5.64E-01	2.16E-01	-6.37E-01	-1.03E+00
2	0	-9.19E+00	-9.15E+00	-1.80E+00	-1.76E+00	2.03E+00	1.98E+00
2	1	-3.69E-02	-4.18E-01	-1.32E+00	1.54E+00	-1.95E+00	-2.12E+00
2	2	3.95E-01	1.42E+00	-2.89E-01	-4.40E-01	3.00E-01	3.40E-01
4	-4	-1.66E-03	1.22E-03	-3.62E-04	-9.31E-05	1.77E-04	2.97E-04
4	-3	-1.70E-03	-4.37E-03	-5.50E-04	9.20E-04	-9.20E-04	-2.38E-03
4	-2	8.09E-03	-2.09E-03	-7.93E-04	-6.91E-05	-3.21E-04	-8.29E-04
4	-1	1.62E-03	1.53E-03	2.12E-03	-1.27E-03	1.76E-04	-8.43E-04
4	0	3.34E-03	3.20E-03	1.83E-04	2.75E-04	-4.71E-04	-5.13E-04
4	1	2.23E-04	7.48E-04	3.44E-03	-4.40E-03	4.14E-03	3.81E-03
4	2	-6.21E-03	-9.07E-03	-5.94E-04	-1.10E-03	2.68E-03	1.86E-03
4	3	-9.05E-03	-1.82E-03	-1.54E-03	2.70E-03	-7.19E-04	-3.00E-03
4	4	2.02E-04	1.67E-03	-2.16E-04	-8.68E-04	6.18E-05	3.38E-04
6	-6	5.93E-05	-2.27E-04	-6.50E-06	1.68E-04	-2.55E-05	-1.19E-07
6	-5	-1.09E-03	1.05E-03	-8.30E-04	-2.43E-05	1.11E-03	1.27E-03
6	-4	-1.26E-04	1.40E-05	2.07E-04	-9.97E-06	2.09E-04	2.51E-04
6	-3	1.37E-04	-1.65E-04	2.26E-05	-1.33E-04	9.58E-04	6.99E-04
6	-2	-4.55E-06	-1.05E-05	3.29E-04	1.55E-04	-5.34E-05	-2.43E-04
6	-1	-3.11E-04	-1.86E-04	2.27E-04	-2.21E-04	1.57E-04	2.54E-04
6	0	4.44E-06	2.34E-05	-3.04E-05	8.33E-07	5.25E-05	9.62E-06
6	1	-4.37E-05	1.47E-04	2.79E-04	-5.41E-04	5.29E-04	8.33E-04
6	2	-5.65E-05	1.06E-04	2.77E-04	1.81E-04	-5.71E-04	-2.19E-04
6	3	-2.01E-04	1.65E-05	-4.79E-04	4.21E-04	-1.15E-04	-4.16E-04
6	4	-1.13E-04	1.24E-04	9.10E-05	1.67E-04	-1.80E-04	-4.18E-05
6	5	4.26E-04	-3.22E-07	-2.24E-04	8.63E-04	-6.78E-04	-5.25E-04
6	6	1.09E-04	-1.37E-04	-5.48E-05	5.59E-05	-3.96E-05	-1.90E-04

*Parameters are given in the local frame for each ion (i.e. *z*-axis coincides with the single-ion quantization axis). When constricting the global Hamiltonian Eq. 6, the local frames should be rotated appropriately.

***Supplementary Table 12**. Stevens factors for Ln^{3+} ions from Ref. 11. α_J, β_J and γ_J, respectively, are the second, fourth and the sixth order Stevens factors.*

Stevens factor	Tb^{3+}	Dy^{3+}	Ho^{3+}	Er^{3+}
α_J	-1.01×10^{-2}	-0.63×10^{-2}	-0.22×10^{-2}	$+0.25 \times 10^{-2}$
β_J	$+1.22 \times 10^{-4}$	-0.59×10^{-4}	-0.33×10^{-4}	$+0.44 \times 10^{-4}$
γ_J	-1.12×10^{-6}	$+1.04 \times 10^{-6}$	-1.29×10^{-6}	$+2.07 \times 10^{-6}$
$10^2 \times \beta_J/\alpha_J$	-1.21	$+0.94$	-1.50	$+1.76$
$10^4 \times \gamma_J/\alpha_J$	$+1.11$	-1.65	$+5.86$	$+8.28$

Supplementary Table 13. The lowest energy spin states of {Tb_2} computed for K^{eff} of 55 cm^{-1}

***E*, cm^{-1}**	**State composition, % $\lvert J_{z,\mathrm{Tb1}}, J_{z,\mathrm{Tb2}}, S_z\rangle$**	**g**
0.0	$100\lvert 6,6,0.5\rangle$	0.0000, 0.0000, 37.9956
248.5	$54\lvert 6,5,0.5\rangle + 40\lvert 5,6,0.5\rangle$	0.0000, 0.0000, 34.9357
316.9	$57\lvert 5,6,0.5\rangle + 43\lvert 6,5,0.5\rangle$	0.0000, 0.0000, 34.9917
492.0	$42\lvert 5,5,0.5\rangle + 25\lvert 6,4,0.5\rangle + 22\lvert 4,6,0.5\rangle$	0.0000, 0.0000, 31.8760
551.8	$47\lvert 4,6,0.5\rangle + 46\lvert 6,4,0.5\rangle$	0.0016, 0.0016, 31.9144
**555.8**	$\mathbf{57\lvert 6,-6,0.5\rangle + 17\lvert 6,-5,0.5\rangle + 13\lvert -6,6,-0.5\rangle}$	**0.0017, 0.0036, 3.6895**
**558.5**	$\mathbf{19\lvert 6,-6,0.5\rangle + 14\lvert -5,6,-0.5\rangle + 51\lvert -6,6,-0.5\rangle}$	**0.0020, 0.0025, 3.7545**
626.2	$51\lvert 5,5,0.5\rangle + 25\lvert 4,6,0.5\rangle + 23\lvert 6,4,0.5\rangle$	0.0000, 0.0000, 31.9758

Supplementary Table 14. The lowest energy spin states of {$\mathbf{Ho_2}$} computed for K^{eff} of 40 cm^{-1}

***E*, cm^{-1}**	**g-tensor**	**cos(α)***
0.0	0.0000, 0.0001, 37.7316	1.00
175.0	0.0106, 0.0207, 31.4631	0.98
227.9	0.0125, 0.0401, 32.6911	1.00
**259.6**	**0.2877, 1.2843, 27.3253**	**0.07**
267.4	0.1288, 0.6484, 31.9671	0.99
295.0	0.1298, 0.2532, 32.3879	0.98
324.7	0.5773, 1.0813, 27.8297	1.00
353.9	2.2027, 5.7440, 18.7264	0.96

* α is the angle between the main direction of ground state and a given higher energy states. Note the strong rotation for the 4th state, which therefore has small μ_z component

Supplementary Table 15. Electrochemical potentials of {**Ln_2**}[a]

{Ln_2}	*E*(+/0)	*E*(0/−)	*E*(−/−2)	*E*(−2/−3)	gap_{EC}[b]
**{Gd_2}**	+0.52	-0.86	-1.45	-1.78	1.38
**{Tb_2}**	+0.51	-0.79	-1.36	-1.71	1.30
**{TbY}**	+0.51	-0.66	-1.33	-1.62	1.11
**{Dy_2}**	+0.52	-0.60	-1.28	-1.58	1.12
**{Ho_2}**	+0.51	-0.54	-1.33	-1.63	1.05
**{Y_2}**	+0.52	-0.52	-1.29	-1.60	1.04
**{Er_2}**	+0.50	-0.42	-1.34	-1.61	0.92

[a] The values are referred *versus* $Fe(Cp)_2^{+/0}$ redox couple. The first reduction and the first oxidation steps are reversible for all {Ln_2} compounds. For many of them reversibility is also found for the second reduction step, but at the third reduction step additional features appear on the reverse scan, which points to the low chemical stability of the $\{Ln_2\}^{3-}$ trianions in *o*-DCB.

[b] Electrochemical gap is defined as the difference between the first oxidation and the first reduction potentials, $gap_{EC} = E(+/0) - E(0/-)$

Supplementary Note 1. Variable temperature X-ray crystallographic analysis of $Dy_2@C_{80}(CH_2Ph)$

The structure measured at 100K showed a fully ordered fullerene cage as well as the attached benzyl group. However, the encapsulated Dy_2 presented as disordered, two positions with fractional occupancies of ca. 0.7 and 0.3 were obtained. To unravel the dynamics of the Dy_2 in the fullerene cage, variable temperature X-ray diffraction from 100 K to 290 K were conducted. As shown in Supplementary Figs. 22 and 23, the fullerene cage and the benzyl group are fully ordered even when the temperature risen to 290 K. On the other hand, temperature-dependent movement of Dy_2 was observed clearly. The Dy_2 site with the highest occupancy showed lower occupancy when the temperature rises, from ca. 0.7 at 100 K to ca. 0.35 at 290 K, simultaneously, new Dy_2 sites appeared and the site occupancies increased with the rising of the temperature. See Supplementary Table 2 and Supplementary Figs. 22 and 23 for details.

Supplementary Note 2. Axiality of the ligand field in {Ln_2} molecules

As follows from *ab initio* calculations (Supplementary Tables 8-11), single-ion magnetic anisotropy in {Ln_2} is rather high. For instance, the total LF splitting for Tb ions is ca 1000 cm^{-1}, and the energy of the first excited KD state is ca 260 cm^{-1}. Ligand field is indeed highly axial, resulting in high-spin ground states of Ising type.

The axiality of the ligand field in {Ln_2} may have several reasons. First, metal atoms transfer their valence electrons to the fullerene cage, resulting in accumulation of the negative charge on carbon atoms coordinated to the endohedral lanthanide ion. Note that this interaction also has considerable covalent contribution via overlap of π-electron density of the fullerene with vacant d-orbital of the lanthanide. Next, covalent Ln-Ln bonding results in a concentration of the electron density between two Ln ions. In Ref. [1] we used a simple point-charge model to show that even relatively small negative charge located between two lanthanide ions may induce rather high axial magnetic anisotropy. Thus, metal-metal bond is important not only for exchange interactions, but also to support the axial ligand field. Finally, lanthanide ions in EMFs have no “equatorial” ligands – the situation which also facilitates imposing of the axial ligand field.

Supplementary Note 3. Magnetic anisotropy and ligand-field states of different lanthanide ions in similar ligand-field environments

The similarity of {Tb_2} and {Dy_2} and the different behavior of {Ho_2} and {Er_2} can be understood by analysis of the LF acting on the individual Ln ions,

$$\hat{H}_{LF_i} = \alpha_J \sum_{m=-2}^{2} A_{2m}^{(i)} < r_i^2 > \hat{O}_{2m} + \beta_J \sum_{m=-4}^{4} A_{4m}^{(i)} < r_i^4 > \hat{O}_{4m} + \gamma_J \sum_{m=-6}^{6} A_{6m}^{(i)} < r_i^6 > \hat{O}_{6m}$$

Here, the Stevens factors α_J, β_J and γ_J are rational numbers depending on S, L, and J and describe the angular shape of the *4f* charge distribution, $< r_i^l >$ are the expectation values of r^l taken with the radial *4f* wave function, $A_{lm}^{(i)}$ are the CF coefficients describing the charge distribution around the Ln^{3+} ion at site *i*, and $\hat{O}_{lm}$ are the standard Stevens operators expressed in polynomials of (J, J_x, J_y, J_z).[14] For the axial parameters, $m = 0$, the parameters *B(l,0)* given in Table S8 are equal to the product of the related Stevens factor, the coefficient $A_{lm}^{(i)}$ and $< r_i^l >$. For other values of *m*, another prefactor must be included as detailed in Ref. 9.

By chemical similarity of the Ln ions, similar charge distributions are expected for all {Ln_2}. Thus, the values of $A_{lm} < r_i^l >$, which are expectation values of the radial *4f* wave function with the *lm*-component of the molecular potential, do not essentially depend on the chosen Ln, except for a minor, smooth reduction with growing atomic number due to decreasing extension of the *4f* radial wave functions (lanthanide contraction).[15] Indeed, the lowest–order axial LF term $A_{20} < r^2 >$ is found from the *B(2,0)* of Supplementary Table 11 and Ref. [1] to obey the expectation. This term decays like 9 (Tb) : 8 (Dy) : 7 (Ho) : 6 (Er). In particular, it has the same, positive sign in all cases.

Supplementary Table 8 shows that states with different J_z hardly mix for Tb ions (and similar was observed for Dy in Ref. [1]). Thus, the LF Hamiltonian is almost diagonal for the Tb and the Dy ions, i.e., only the axial LF parameters are relevant. We evaluated the LF levels of the Tb and Dy ions using only the axial LF parameters and assuming the non-axial parameters to be zero. The resulting levels (curves denoted as axial 2, 4, 6) are compared with the levels obtained by our *ab-initio* calculations (crosses) in Supplementary Fig. 43. As expected for the case of small mixing, the diagonal LF terms describe the *ab-initio* levels very well.

In order to estimate the relative importance of the individual axial terms (*l* = 2, 4, 6), we repeated the calculation with fourth-order term set to zero (curves denoted as axial 2, 6) and finally with both fourth- and sixth-order terms set to zero (curves denoted as axial 2). For the Tb ions we find that the quantum chemical data are very well described by considering only the second-order axial LF term; the fourth order provides a minor correction and the sixth order has no influence that would be visible at the scale of the plot. For the Dy ions as well, the second order alone describes the levels reasonably well. However, a minor correction is provided by the fourth-order term and a somewhat larger correction by the sixth order.

The relatively larger influence of the sixth-order term in Dy as compared to Tb can be understood from a combination of two factors. First, the sixth-order term gains a larger importance in Dy due to the larger *J*-value of its ground state multiplet, $= 15/2$, compared to $J = 6$ for Tb. This value enters the expectation values of the Stevens operators in the power of *l*, providing an enhancement by a factor of 2.4 for the

importance of order six vs. order two. Second, a factor of modulus 1.5 in favor of the Dy sixth-order contributions is gained by the ratio of the Stevens factors γ_J/α_J, see Supplementary Table 12. In a very similar manner one can understand that the fourth-order term has more or less the same influence on the Tb and the Dy ions. Here, a factor of 1.6 due to the different *J*-values is partly compensated by a factor of 0.8 due to the ratio of the Stevens factors β_J/α_J , see Supplementary Table 12.

Summarizing the discussion for the Tb and the Dy ions, the whole LF spectrum and the LF states of Tb^{3+} in {Tb_2} can be described by a **single** parameter *B(2,0)* and the same holds, with a grain of salt, for Dy^{3+} in {Dy_2}. The sign of this parameter determines, whether the magnetic anisotropy is of uniaxial type, i.e., the ground state is $|J_z| = J$, or if it is of easy-plane type with a ground state $|J_z| = 0$ or ½. For the two discussed ions, *B(2,0)* is negative, implying a uniaxial ground state. The negative sign of *B(2,0)* for both cases comes about by the same, positive sign of A_{20} for both systems combined with the same, negative sign of the related Stevens factors.

Turning to the case of the Ho^{3+} ions, it can be noted that the second-order Stevens factor α_J is negative as in Tb^{3+} and Dy^{3+}. Thus, given the same sign of A_{20} as in the other cases, also *B(2,0)* is negative and Ho has a high-spin ground state like the previous cases. However, the effect of the sixth-order axial crystal field term is much stronger than in Tb or Dy, since the ratio γ_J/α_J provides a factor of 5.3 (as compared with Tb) and the larger $J = 8$ provides a factor of 3.2. Calculation of the LF levels from the axial terms alone (not shown) shows that the high-spin ground state is (just) not spoilt by the higher-order terms. However, by virtue of the larger pre-factors, the non-axial sixth-order LF parameters now produce a strong mixing of different J_z states, see Supplementary Table 9. This mixing is present even in the ground state. It is responsible for an important contribution to transitions between (quasi) degenerate states. The importance of fourth-order terms is slightly enhanced, if compared to Tb and Dy, but their impact is much smaller than that of other terms.

Finally, the second-order Stevens factor of Er^{3+} is positive, opposite to all the other cases. Thus, an easy-plane ground state is realized in Er which is not compatible with SMM behavior. The influence of sixth-order contributions to the LF Hamiltonian of Er^{3+} in {**Er_2**} is similar to the case of Ho, since the ratio of the Stevens factors γ_J/α_J is somewhat larger in Er^{3+} than in Ho^{3+}, while the value of $J = 15/2$ is smaller than in Ho. Fourth-order terms have the same, minor importance as in the other systems.

Summarizing all the considered cases, the LF ground states for {Ln_2} are solely determined by the sign of the related second-order Stevens factors, encoding the different shapes of the Ln-*4f* shells. **Moreover, it is possible, in a decent approximation, to describe all LF spectra by one single parameter A_{20},** which is determined by the quadrupolar potential at the Ln sites, and appropriately scaled by the radial expectation value of the specific Ln-4f wave function. A refined description, which is needed to account for mixing of the pure J_z states in the cases of Ho and Er, however, has to include all sixth-order terms.

The dominance of the axial second-order LF term can be attributed to the large, almost axially distributed charge on the Ln dimers, including the single-electron σ-bond. The less important, though still significant for the Ho and Er systems, sixth-order contributions (axial and non-axial) can be attributed to the charge distribution on the carbon cage. This means that a transfer of the Ln dimers to other kinds of carbon cages will influence mainly the less important sixth-order terms and keep the present results qualitatively unaffected.

Supplementary Note 4. Spectra of Model Hamiltonians

Although the simple *Ising*-type Hamiltonian (Supplementary Equation 1) gives insights mostly in the ground state, the formal analysis of the complete Hamiltonian (Supplementary Equation 1) spectra can be made, and transition probability between different states *i* and f can be estimated as (see code PHI[10] for details):

$$T_{if} = \frac{1}{3}\sum_{\alpha=x,y,z}\left|\langle i\middle|\hat{H}_Z(B_\alpha)\middle|f\rangle\right|^2$$

{Tb_2} and {Ho_2} molecules, with [Ln^{3+}–*e*–Ln^{3+}] spin distribution, are Kramers systems. Thus, the ground state tunneling is protected in both cases (Supplementary Figs. 47, 57) and shows infinitesimal tunneling probability either in case of {Tb_2} or {Ho_2}. On a whole the {Tb_2} molecule represents a similar Hamiltonian spectrum once reported for {Dy_2}[1]. Transitions probabilities are very low up to 6-7 KD-states and even after this point they are not very high. Assuming the K^{eff} coupling in (Supplementary Equation 1) of 55 cm^{-1} one can estimate the barrier for the effective Orbach process, through the semi-degenerate exchange-excited doublets to be of 555.8 and 558.5 cm^{-1} (~800 K). For the non-collinear system, the Hamiltonian S1 is not expected to be fully adequate, but still, the formal spectrum and transition probabilities can be computed and the effective Orbach process barrier can be estimated. For the K^{eff} value of 40 cm^{-1}, there is a state with the energy of 259.6 cm^{-1} (374 K), which has an almost perpendicular orientation of the main anisotropy axis with respect to the ground state. Experimentally, an Orbach barrier of ~330 K is found for {Ho_2}.

Supplementary Methods

Magnetic properties

Magnetic susceptibility in the further discussion is defined as $\chi = M/H$ both in experiment and in theory. Note that in high field the *M/H* ratio is significantly different from differential susceptibility defined as a derivative of magnetization with respect to the external field.

Simulations of magnetic properties are based on the spin Hamiltonian (all calculated curves are powder-averaged):

$$\hat{H}_{\text{spin}}(\{\text{Ln}_2\}) = \hat{H}_{LF_1} + \hat{H}_{LF_2} - 2K^{\text{eff}}\hat{s}\left(\hat{J}_{\text{Ln}_1} + \hat{J}_{\text{Ln}_2}\right) \quad (1)$$

where ligand field parameters in $\hat{H}_{LF_i}$ are obtained from *ab initio* calculations, and exchange constant K^{eff} is the only unknown parameter. The value of K^{eff} is varied to find the best fit to the experimental data.

For $\{Gd_2\}$, the spin Hamiltonian is reduced to the following form:

$$\hat{H}_{\text{spin}}(\{\text{Gd}_2\}) = -2K^{\text{eff}}\hat{s}\left(\hat{S}_{\text{Gd}_1} + \hat{S}_{\text{Gd}_2}\right) \quad (2)$$

Thus leaving only one unknown parameter, effective exchange constant K^{eff}.

For {TbY}, the spin Hamiltonian is reduced to the following form:

$$\hat{H}_{\text{spin}}(\{\text{TbY}\}) = \hat{H}_{LF_{\text{Tb}}} - 2K^{\text{eff}}\hat{s} \cdot \hat{J}_{\text{Tb}} \quad (3)$$

where ligand-field parameters in $\hat{H}_{LF_{\text{Tb}}}$ are obtained from *ab initio* calculations.

In the fitting of the magnetic data we used the followed strategy: χT curve measured for a given $\{Ln_2\}$ compound was fitted for only one value of the magnetic field, 1 T, to determine K^{eff}. This K^{eff} value was then used to calculate χT curves for other values of the magnetic field (3, 5, and 7 T) as well as to calculate magnetization curves at different temperatures. The agreement of the model and experiment is considered to be good, when a single K^{eff} value can give a good agreement for the whole set of experimental data.

Due to the small mass of the sample, reliable estimations of the absolute magnetization values are possible only for $\{Dy_2\}$ (ref. [1]), $\{Er_2\}$ and $\{Ho_2\}$, which show the highest yield in the synthesis. For other sample uncertainties in the mass determination introduced during the sample preparation are too large to allow accurate determination of the absolute magnetization values. We therefore use arbitrary units for these $\{Ln_2\}$ molecules

DFT calculations

We used the VASP code, version 5.0, to optimize the molecular structures at the PBE-D level using PAW pseudopotentials with standard cutoffs as recommended.[3, 4, 5, 6] The 4f shells of the lanthanide elements do not contribute to chemical bonding. Thus, we included the 4f shell in the core potential, i.e., we used the so-called open-core approximation (here, implemented as unpolarized potential). The SCF calculations accounted for spin polarization of the valence states. This procedure is expected to provide realistic results for structures involving Ln ions.[15] The pseudopotential configuration $5p^6 6s^2 5d^1$ was used for all Ln atoms. All molecular structures were optimized such that the residual forces for all atoms were below 10^{-4} *eV/Å*.

The broadening parameter was chosen to be 0.2 *eV*. The size of the cubic periodic calculation box was 25 $Å^3$.

Ab initio calculations

Ab initio energies and wave functions of CF multiplets for the {LnY}⁻ molecule and model systems have been calculated using the quantum chemistry package MOLCAS 8.0.[7] Single point complete active space self-consistent field with spin-orbit interactions calculations (CASSCF/SO-RASSI) were done to derive ab initio ligand field parameters (note that we use the term "ligand field" instead of "crystal field" to avoid possible confusion with the intermolecular interactions in crystals).[8] Mixed atomic natural extended relativistic basis set (ANO-RCC) was employed with the minimal basis option for C and H atoms, and VDZ-quality of Y, Ln metals in the cluster. The use of {LnY}⁻ anions instead of neutral {LnY} molecule allows one to limit active space only to the 4*f* shell. Calculations for the neutral {LnY} molecule would require inclusion of the unpaired valence electron and corresponding valence orbital into the active space, which makes *ab initio* calculations less tractable. Besides, the crystal field parameters extracted from such calculations would then lose their clear physical meaning.

Depending on f-shell populations of the Ln-ions there are three ground state multiplets $^7F_6$, $^5I_8$ and $^4I_{15/2}$ for Tb (with f^8), Ho (with f^{10}) and Er(f^{11}) respectively. Being non-Kramers, with an even number of electrons, ions Tb and Ho are represented by energetically close singlets, which can be grouped into several pseudo-KD. At the same time the Er-system, as a Kramers-system, has a full set of eight low-lying Kramers doublets.

Supplementary Tables 7 summarizes the CAS compositions, RASSI-SO setting and ANISO pseudo-spin mapping options for the different {LnY}⁻ molecule. For example, the active space of the CASSCF calculations includes eleven active electrons and the seven active orbitals (e.g. CAS (11,7)) was considered for {ErY}⁻molecule. All 35 quartets and 112 doublets were used in the state-averaged CASSCF runs and when all 147 states were mixed in the state interaction problem with spin-orbit interaction included. The single ion magnetic properties and CF-parameters were calculated with use of SINGLE_ANISO module with mapping on eight low-lying Kramers doublets of Er^{+3}. The results and properties for KD and pseudo-KD systems are shown in Supplementary Tables 8-10.

Supplementary References

1. Liu, F. *et al.* Single molecule magnet with an unpaired electron trapped between two lanthanide ions inside a fullerene. *Nat. Commun.* **8**, 16098 (2017).

2. Krylov, D. *et al.* Magnetization relaxation in the single-ion magnet $DySc_2N@C_{80}$: quantum tunneling, magnetic dilution, and unconventional temperature dependence. *Phys. Chem. Chem. Phys.* **20**, 11656-11672 (2018).

3. Kresse, G. & Hafner, J. Ab initio molecular dynamics for liquid metals. *Phys. Rev. B* **47**, 558-561 (1993).

4. Hafner, J. Ab-initio simulations of materials using VASP: Density-functional theory and beyond. *J. Comput. Chem.* **29**, 2044-2078 (2008).

5. Perdew, J. P., Burke, K. & Ernzerhof, M. Generalized gradient approximation made simple. *Phys. Rev. Lett.* **77**, 3865-3868 (1996).

6. Grimme, S. Density functional theory with London dispersion corrections. *WIREs Comput. Mol. Sci.* **1**, 211-228 (2011).

7. Aquilante, F. *et al.* Molcas 8: New capabilities for multiconfigurational quantum chemical calculations across the periodic table. *J. Comput. Chem.* **37**, 506-541 (2016).

8. Ungur, L. & Chibotaru, L. F. Ab Initio Crystal Field for Lanthanides. *Chem.-Eur. J.* **23**, 3708-3718 (2017).

9. Chibotaru, L. F. & Ungur, L. Ab initio calculation of anisotropic magnetic properties of complexes. I. Unique definition of pseudospin Hamiltonians and their derivation. *J. Chem. Phys.* **137**, 064112 (2012).

10. Chilton, N. F., Anderson, R. P., Turner, L. D., Soncini, A. & Murray, K. S. PHI: A powerful new program for the analysis of anisotropic monomeric and exchange-coupled polynuclear d- and f-block complexes. *J. Comput. Chem.* **34**, 1164-1175 (2013).

11. Stevens, K. W. H., Matrix Elements and Operator Equivalents Connected with the Magnetic Properties of Rare Earth Ions. *Proceedings of the Physical Society A* **65**, 209 (1952).
